# SSH Analysis of Endosperm Transcripts and Characterization of Heat Stress Regulated Expressed Sequence Tags in Bread Wheat

**DOI:** 10.3389/fpls.2016.01230

**Published:** 2016-08-17

**Authors:** Suneha Goswami, Ranjeet R. Kumar, Kavita Dubey, Jyoti P. Singh, Sachidanand Tiwari, Ashok Kumar, Shuchi Smita, Dwijesh C. Mishra, Sanjeev Kumar, Monendra Grover, Jasdeep C. Padaria, Yugal K. Kala, Gyanendra P. Singh, Himanshu Pathak, Viswanathan Chinnusamy, Anil Rai, Shelly Praveen, Raj D. Rai

**Affiliations:** ^1^Division of Biochemistry, Indian Agricultural Research InstituteNew Delhi, India; ^2^Centre for Agricultural Bio-Informatics, Indian Agricultural Statistics Research InstituteNew Delhi, India; ^3^National Research Center on Plant BiotechnologyNew Delhi, India; ^4^Division of Genetics, Indian Agricultural Research InstituteNew Delhi, India; ^5^Centre for Environment Science and Climate Resilient Agriculture, Indian Agricultural Research InstituteNew Delhi, India; ^6^Division of Plant Physiology, Indian agricultural Research InstituteNew Delhi, India

**Keywords:** abiotic stress, differential expression, SSH library, terminal heat stress, *Triticum aestivum*, SAGs, DEGs, differentially expressed proteins (DEPs)

## Abstract

Heat stress is one of the major problems in agriculturally important cereal crops, especially wheat. Here, we have constructed a subtracted cDNA library from the endosperm of HS-treated (42°C for 2 h) wheat *cv*. HD2985 by suppression subtractive hybridization (SSH). We identified ~550 recombinant clones ranging from 200 to 500 bp with an average size of 300 bp. Sanger's sequencing was performed with 205 positive clones to generate the differentially expressed sequence tags (ESTs). Most of the ESTs were observed to be localized on the long arm of chromosome 2A and associated with heat stress tolerance and metabolic pathways. Identified ESTs were BLAST search using Ensemble, TriFLD, and TIGR databases and the predicted CDS were translated and aligned with the protein sequences available in pfam and InterProScan 5 databases to predict the differentially expressed proteins (DEPs). We observed eight different types of post-translational modifications (PTMs) in the DEPs corresponds to the cloned ESTs-147 sites with phosphorylation, 21 sites with sumoylation, 237 with palmitoylation, 96 sites with S-nitrosylation, 3066 calpain cleavage sites, and 103 tyrosine nitration sites, predicted to sense the heat stress and regulate the expression of stress genes. Twelve DEPs were observed to have transmembrane helixes (TMH) in their structure, predicted to play the role of sensors of HS. Quantitative Real-Time PCR of randomly selected ESTs showed very high relative expression of *HSP17* under HS; up-regulation was observed more in wheat *cv*. HD2985 (thermotolerant), as compared to HD2329 (thermosusceptible) during grain-filling. The abundance of transcripts was further validated through northern blot analysis. The ESTs and their corresponding DEPs can be used as molecular marker for screening or targeted precision breeding program. PTMs identified in the DEPs can be used to elucidate the thermotolerance mechanism of wheat—a novel step toward the development of “climate-smart” wheat.

## Introduction

Wheat is the staple food grain crop of half of the world providing 12% of the carbohydrate and 40% of calories in the diet. Of the abiotic stresses that affect the agriculture, high temperature is the most important stress adversely affecting the wheat growth and yield (Kumar et al., 2013). About 7 million hectare areas is affected by heat stress (HS), while terminal heat is a problem in about 40% of the irrigated wheat growing areas of the world (Joshi et al., 2007). Wheat (*Triticum aestivum* L.) is highly sensitive to terminal HS during the reproductive stages i.e., fertilization, grain-filling, and maturation stages. Heat stress causes drying of stigmatic surface, pollen sterility, sterilization, pseudo-seed setting, fragmented starch granules, empty pockets in endosperm, which ultimately decrease the quality of the grain (Barakat et al., 2007). Several thermotolerant genotypes of wheat are known and in most cases, the tolerance is associated with the altered expression of stress-associated genes (SAGs) (Sanghera et al., 2011; Kumar et al., 2013). Stress associated genes/proteins (SAGs/SAPs) expressed under different abiotic/biotic stresses play very significant roles in protecting the cell and its components from denaturation or aggregation under the HS (Kumar et al., 2012, 2013). Some of the SAGs/SAPs identified in wheat under HS are heat-shock proteins (HSPs), antioxidant enzymes, heat-responsive transcription factors (TFs), and signaling molecules (MAPKs, CDPKs).

Suppression subtractive hybridization (SSH; Diatchenko et al., 1996) is an efficient and productive approach for the identification and cloning of differentially expressed genes (DEGs). This technique identifies the abundant DEGs as well as rare transcripts in order to facilitate the identification of novel genes (Boominathan et al., 2004; Clement et al., 2008; Liu et al., 2008). SSH has been widely used in the past to identify differential expression of genes in maize (*Zea mays* L.; Zhang et al., 2004; Nguyen et al., 2009), cucumber (*Cucumis sativus*; Terefe and Tatlioglu, 2005), mustard (*Brassica napus*; Wu et al., 2007), wheat (*T. aestivum*; Li et al., 2008), Mulberry (Gulyani and Khurana, 2011) and rice (*Oryza sativa*; Gorantla et al., 2007; Jiang et al., 2009). SSH is preferred in cases where genome sequence information is not available. The present investigation aimed at identifying the differentially expressed ESTs from the endospermic tissue of thermotolerant wheat *cv*. HD2985, their expressional analysis through quantitative real-time PCR (qRT-PCR) and northern blotting, identification of post-translation modifications (PTMs), and correlating the findings with the heat stress-tolerance mechanism of wheat.

## Materials and methods

### Plant material and stress treatment

Seeds of wheat *cvs*. HD2985 (thermotolerant) and HD2329 (thermosensitive) were procured from the Division of Genetics, Indian Agricultural Research Institute, New Delhi. Pre-treated seeds (Bavistin @ 0.5% for 30 min) were sown in six pots (in group of two labeled as control and HS-treated), inside the regulated chamber (optimum temperature regime of 26/22°C, humidity 60%, photoperiod 16 h, and light intensity of 350 μmol/m^2^/s) in the National Phytotron Facility, IARI, New Delhi. Plants (3 pots) were exposed to HS during the grain-filling stage (Feekes scale—11.2) inside the microprocessor regulated heating chamber in sinusoidal mode; temperature was increased from 25° to 42°C with an increment of 1°C per 5 min, till it reached the desired HS temperature. The temperature was maintained at 42°C for 2 h and further decreased in the similar fashion. The control and HS-treated endospermic tissues were collected in triplicate and immediately frozen in liquid nitrogen for further downstream analysis.

### Isolation and purification of poly (A)^+^ RNA

Total RNA was extracted from the collected samples in triplicates using the Reflex™ total RNA isolation kit (Genei, India). The quality and concentration of the isolated total RNA was estimated using Qubit™ 2.0 Fluorometer (Invitrogen, UK). RNA integrity was also verified by electrophoretic separation on 1.2% agarose gel. Total RNA having OD 260/280 ratio of more than 2.0 was used for the mRNA isolation. Poly (A) RNA (mRNA) was isolated and purified from the total RNA according to the protocol of NucleoTrap mRNA midi kit (Macherey-Nagel, Clonetech, Takara, Germany). The purified mRNA was further lyophilized and dissolved in low volume of RNase-free water to increase the concentration.

### Construction of cDNA library by suppression subtractive hybridization (SSH)

Subtracted cDNA library of wheat *cv*. HD2985 was constructed using Clontech PCR-Select cDNA subtraction kit (Clontech Laboratories, USA), following the manufacturers protocol. In brief, tester (HS-treated) and driver (control) double stranded cDNAs were prepared from 2 μg of mRNA. Tester and driver cDNAs were separately digested with *Rsa*I to obtain shorter blunt ended molecules. Two tester populations were created by ligating two aliquots of diluted tester cDNA with two different adaptors (adaptors 1 and 2R) as given in the kit, separately. First step hybridization was performed as per the instructions given by the manufacturers. In brief, each tester population was mixed with an excess of digested driver cDNA and the samples were heat-denatured and then allowed to anneal at 68°C for 8 h. Two samples from the first hybridization reaction were mixed together, and denatured driver cDNA was added for overnight hybridization in order to enrich the differentially expressed sequences. Differentially expressed cDNAs with different adaptor sequences at two ends, were selectively amplified by PCR, and a second PCR was done with the nested primers to further reduce the background. The secondary PCR products of SSH were inserted into pGEM-T easy vector (Promega, Madison, WI, USA). However, prior to the insertion, the forward subtracted PCR cDNA mix was incubated at 72°C for an extra 1 h, with additional dATP and Taq DNA polymerase (Invitrogen, Calsbad, USA) to ensure that most of the cDNA fragments contained 3'A overhangs; the recombinants were transformed into NEB 5-alpha competent *E. coli* cells (NEB, UK). The transformed bacteria were plated onto Luria Agar plates with 100 μg/ml ampicillin, 100 μM IPTG and 50 μg/ml X-Gal, and incubated at 37°C for 18 h, until colonies were visible; the plates were transferred to the refrigerator (4°C) in order to visualize the blue/white staining. The white colonies, the hypothetical *E. coli* cells including recombinant clones, were selected for further analysis through plasmid isolation, PCR, restriction by *Eco*RI, and Sanger's sequencing.

### Screening of differentially expressed cDNA clones from the subtracted library

A total of 550 hypothetical recombinant clones were streaked onto plates containing LA medium with 100 μg/ml ampicillin, and incubated overnight at 37°C. Colony PCR was done for the screening of the target inserts in 20 μl reactions containing 2 μl of 10 × PCR reaction buffer, 0.6 μl of nested primer 1, 0.6 μl of nested primer 2R, and 0.4 μl of 10 μM dNTP mix (Clontech, USA) under the following cycle condition: 94°C for 30 s, 23 cycles of 94°C for 30 s, 68°C for 3 min. Some of the clones were screened by restriction digestion (*Eco*RI) as release of ~200–500 bp was observed. After amplification and restriction digestion analysis, 205 positive clones were randomly selected for the Sanger's sequencing.

### Sequencing and expressed sequence tags (ESTs) analysis

The randomly selected 205 clones were subjected to sequencing with the modified M13 reverse primer (5′-AGCGGATAACAA TTTCACACAGG-3′) using Sanger's di-deoxy method. Each sequence was, however, screened for overall base quality, and the contaminating vector; mitochondrial, ribosomal, and *E. coli* sequences were removed. The sequences of the cDNA inserts were compared with the GenBank non-redundant translated query-protein databases (BLASTx), after stripping out the vector and primer sequences (Altschul et al., 1997).

### Chromosomal localization and *in silico* characterization of cloned ESTs

All the EST sequences identified in present investigation were mapped on the genome of *Triticum* downloaded from Ensemble genome databases (http://www.ensembl.org, IWGSC2; 2014-11, International Wheat Genome Sequencing Consortium). Ensemble plant is an important resource for *Triticeae* (Bolser et al., 2015), which is very popular for retrieving the chromosomal location, position (start-end), transcript name, and gene ontologies. Further, the clones were annotated using Triticeae Full-Length CDS Database (TriFLDB) (http://trifldb.psc.riken.jp/) and TIGR (http://jcvi.org/wheat/wheat_gaad.shtml) databases.

For retrieval of significant differentially expressed proteins (DEPs), CDS of all the ESTs were first translated into protein sequences by using Expasy tool (http://web.expasy.org/), and the longest frame having a single open reading frame without gap was selected. The DEP search was performed using the pfam database (http://pfam.xfam.org/; Finn et al., 2008) and InterProScan 5 database (http://www.ebi.ac.uk/interpro/; Mitchell et al., 2014), with expectation cut off (*E*-value) 1.0 as the threshold, and only significant proteins were considered as valid.

Subcellular localizations of DEPs (predicted based on the CDS) were observed by the CELLO v.2.5: Subcellular Localization Prediction of Eukaryotic Proteins (http://cello.life.nctu.edu.tw/; eukaryote; Yu et al., 2006), and Plant-mPLoc (http://www.csbio.sjtu.edu.cn/bioinf/plant-multi; Chou and Shen, 2008). The gene ontology enrichment analysis was performed by AgriGO: the Singular Enrichment Analysis (SEA) (http://bioinfo.cau.edu.cn/agriGO/) and the GOEAST (http://omicslab.genetics.ac.cn/GOEAST/), with *P*-value threshold-0.05. The pathway enrichment analysis was performed by the PathExpress (http://g6g-softwaredirectory.com/apps/bio/cross-omics/pathway-analysis-grns/ListingsByAppCOPathwayAnalys.php), using Affymetrix Wheat Genome Array (*T. aestivum* L.) as the background genome Array. The Genevestigator Expression Database (Hruz et al., 2008) was used for the Meta-Analysis of the cloned ESTs under diverse stress conditions, tissue-wise, and others. TMHMM trans-membrane motifs were detected by the TMHMM Server (http://www.cbs.dtu.dk/services/TMHMM) with the default settings of the software. Different packages of online server of GPS-polo 1.0 (Group-based Prediction System; http://polo.biocuckoo.org/) were used for the prediction of post-translational modification sites (Blom et al., 1999).

### Expression profiling of selected ESTs using quantitative real-time PCR (qRT-PCR)

We randomly selected nine genes for the expression analysis by qRT-PCR in contrasting wheat *cvs*. HD2985 and HD2329 under the HS. First strand cDNA was synthesized from the isolated total RNA, using oligo dT primers and the Superscript II reverse transcriptase (Invitrogen, UK) according to the manufacturer's instructions. The templates were diluted to a final concentration of 20 ng/μl. Primers were designed from the deduced EST sequences—heat shock proteins (*HSP17, HSP70, DnaJ*), heat-responsive transcription factors (seed-specific HSF), antioxidant enzymes (superoxide dismutase, catalase), genes involved in signaling (calcium dependent protein kinase), stress-associated hypothetical protein, and peptidyl propyl isomerase gene (*PPIase)*—using Prime 3 primers designing software (Premier Biosoft, USA; Table S1). Expression analysis was performed using three biological and three technical replicates. Quantitative RT-PCR was performed in 20 μl reactions consisting of 0.4 μl of 10 mM gene specific primers (forward and reverse), 1 μl (20 ng/μl) of cDNA as template, and the SYBRGreenER qPCR SuperMix Universal (Invitrogen, UK). The CFX96 Real-Time PCR platform (Bio Rad, UK) was used for the expression profiling. The thermal profile for qPCR was: 3 min at 95°C, 39 cycles each of 95°C for 15 s, 60°C for 30 s, and 72°C for 15 s, followed by plate reads. The expression of wheat β*-actin* gene (accession no. AB181991.1) was used as internal standards for normalizing the Ct-values. The Comparative Ct (2^−ΔΔCt^) method was used to calculate the changes in gene transcript as a relative fold difference between experimental and calibrator sample (Pfaffl, 2001). Primer specificity and formation of primer-dimers were monitored by dissociation curve analysis, and agarose gel (3%) electrophoresis.

### Northern blot analysis

Total RNA (2 μg) was isolated from the control and HS-treated samples of wheat *cvs*. HD2985 and HD2329. A lane of driver (control) and one of tester (heat stress) were used for the electrophoresis on 1.0% formaldehyde agarose gel; the samples were then transferred onto nylon membrane using iBlotter (Invitrogen, UK). Differentially expressed candidate cDNAs (*HSP70, HSP17, HSF, DnaJ, PPIase, MIPS*, LRR like protein, putative zinc finger protein, and hypothetical protein) were first amplified by PCR as described above, and then labeled with 50 μCi [α-^32^p] dCTP using DecaLabel DNA Labeling Kit (Thermo Fisher Scientific, Fermentas, UK); labeled probes were used for the northern blot hybridization as per the protocol in Sambrook et al. (1989).

## Results

### Construction of the suppression subtractive hybridization (SSH) libraries

In heat stress-responsive forward SSH (FSH) libraries of wheat endosperm tissue, we identified about 550 individual recombinants. From 350 individual white bacterial colonies, cDNA inserts were analyzed, and we found inserts of 250–500 bp in 205 clones with an average size of 300 bp (sequences are shown in the Supplementary File).

### Gene ontology (GO) and pathway-enrichment analysis

**Figure 1:**
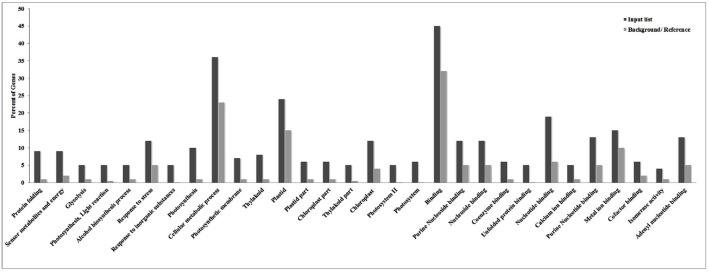
**Gene ontology (GO) annotation analysis of cloned Expressed Sequence Tags (ESTs) revealed cellular metabolic processes and plastid-associated genes to be most altered under the HS**.

ESTs identified in the present investigation were subjected to GO annotation analysis and we observed binding followed by cellular metabolic processes and plastid-associated genes to be most altered under HS (Figure 1). Similarly, genes associated with processes like nucleotide binding, stress response, and photosynthesis were observed to share significant variations among other identified DEGs under HS. ReviGO prediction (*p* < 0.05) showed most of the identified DEGs to be associated with photosynthesis followed by protein folding.

**Figure 2:**
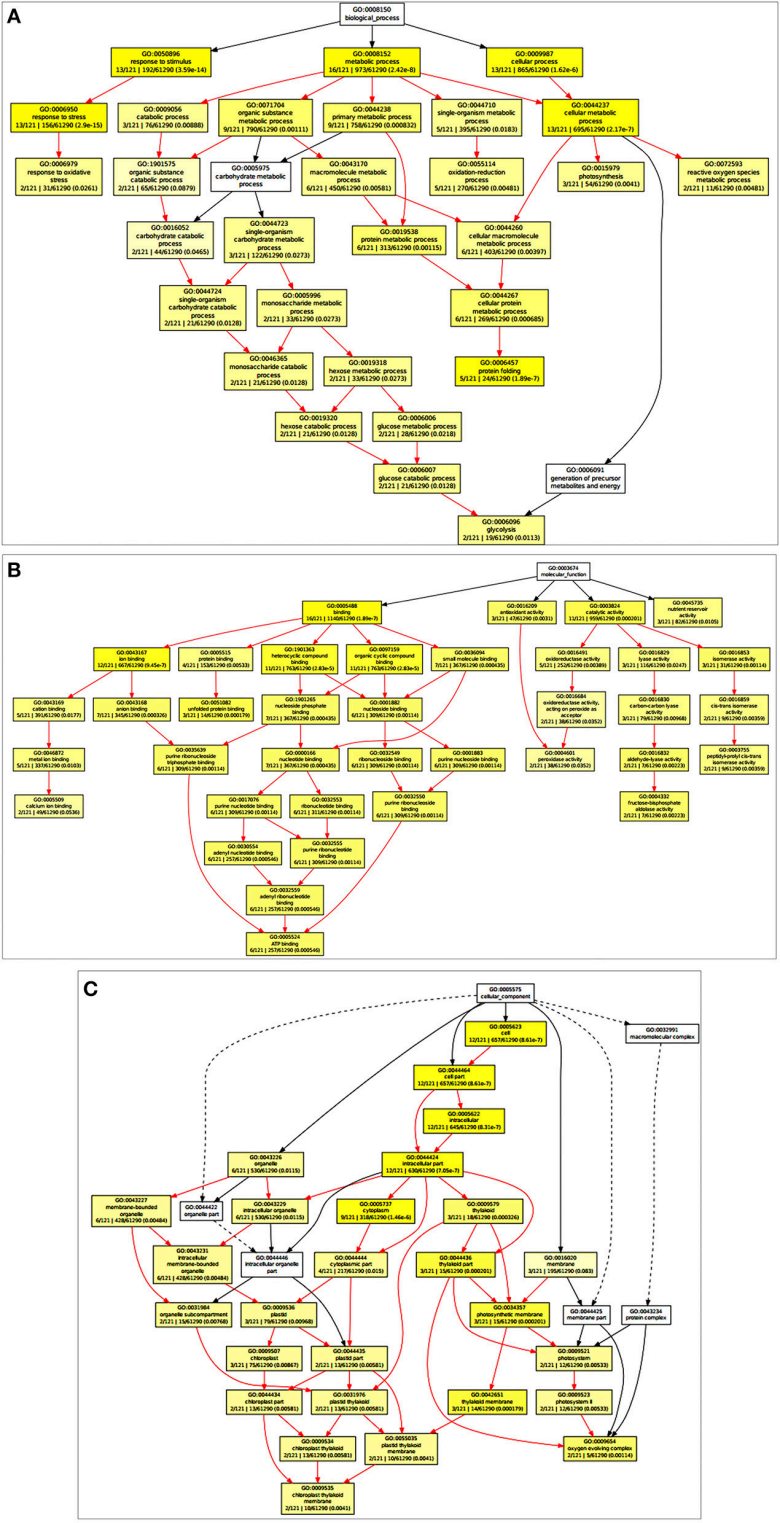
**Gene Ontology Enrichment Analysis Software Toolkit (GOEAST) analysis of the cloned Expressed Sequence Tags (ESTs) to generated separate graph for each of the three GO categories, i.e., biological process, molecular function and cellular component. (A)** ESTs associated with biological process category with significant *p*-value, **(B)** ESTs associated with molecular function, **(C)** ESTs associated with cellular component with high enrichment.

Based on the Gene Ontology Enrichment Analysis Software Toolkit (GOEAST) using Singular Enrichment Analysis (SEA) tool, we generated in a separate graph for each of the three GO categories, i.e., biological process, molecular function, and cellular component (Figures 2A–C). Under the biological process category, we observed ESTs associated with photosynthesis (GO: 0015979), metabolic processes (GO: 0008152), glucose metabolism (GO: 0006006), response to the oxidative stress (GO: 0006979), and transcription regulation (GO: 0006355) with significant *p*-value (Figure 2A; Table 1). Based on the molecular function, ESTs were associated with transcription factors (GO: 0003700), ATP binding (GO: 0005524), metal ion binding (GO: 0046872), and kinase activity (GO: 0004672; Figure 2B). Under cellular component, we observed photosynthetic membrane (GO: 0034357), photosystem (GO: 0009521), light-harvesting complex (GO: 0030076), and oxygen-evolving complex (GO: 0009654) with high enrichment (Figure 2C).

**Table 1:** **Enriched biological processes related Gene Ontology terms**.

**GO term**	**Ontology**	**Description**	***p*-value**	**FDR**
GO:0015979	P	Photosynthesis	2.20E-10	1.40E-08
GO:0006457	P	Protein folding	4.00E-09	1.20E-07
GO:0019684	P	Photosynthesis, light reaction	1.20E-05	0.00019
GO:0010035	P	Response to inorganic substance	9.50E-06	0.00019
GO:0006091	P	Generation of precursor metabolites and energy	0.0001	0.0012
GO:0044237	P	Cellular metabolic process	0.00076	0.0079
GO:0006950	P	Response to stress	0.00091	0.0081
GO:0006096	P	Glycolysis	0.0012	0.0086
GO:0046165	P	Alcohol biosynthetic process	0.0012	0.0086
GO:0019320	P	Hexose catabolic process	0.003	0.016
GO:0034641	P	Cellular nitrogen compound metabolic process	0.0035	0.016
GO:0044275	P	Cellular carbohydrate catabolic process	0.0037	0.016
GO:0006007	P	Glucose catabolic process	0.003	0.016
GO:0016051	P	Carbohydrate biosynthetic process	0.0032	0.016
GO:0046365	P	Monosaccharide catabolic process	0.0045	0.017
GO:0044260	P	Cellular macromolecule metabolic process	0.0053	0.017
GO:0046164	P	Alcohol catabolic process	0.0047	0.017
GO:0050896	P	Response to stimulus	0.005	0.017
GO:0006006	P	Glucose metabolic process	0.0052	0.017
GO:0034637	P	Cellular carbohydrate biosynthetic process	0.0062	0.019
GO:0008152	P	Metabolic process	0.0093	0.027
GO:0016052	P	Carbohydrate catabolic process	0.01	0.028
GO:0044262	P	Cellular carbohydrate metabolic process	0.015	0.04
GO:0006066	P	Alcohol metabolic process	0.016	0.041
GO:0019318	P	Hexose metabolic process	0.017	0.043
GO:0043170	P	Macromolecule metabolic process	0.023	0.05
GO:0044267	P	Cellular protein metabolic process	0.022	0.05
GO:0009987	P	Cellular process	0.022	0.05

Putative conserved domain (CD) and protein search using the Pfam and InterProScan 5 databases showed the presence of domains like Chlorophyll A-B binding protein, AAA+ ATPase, Photosynthetic reaction center, L and M subunits, ATP-binding region, Photosystem II manganese-stabilizing protein PsbO, Transcription factor, and MADS-box (Table S2).

### Chromosomal localization and mapping of identified ESTs

**Figure 3:**
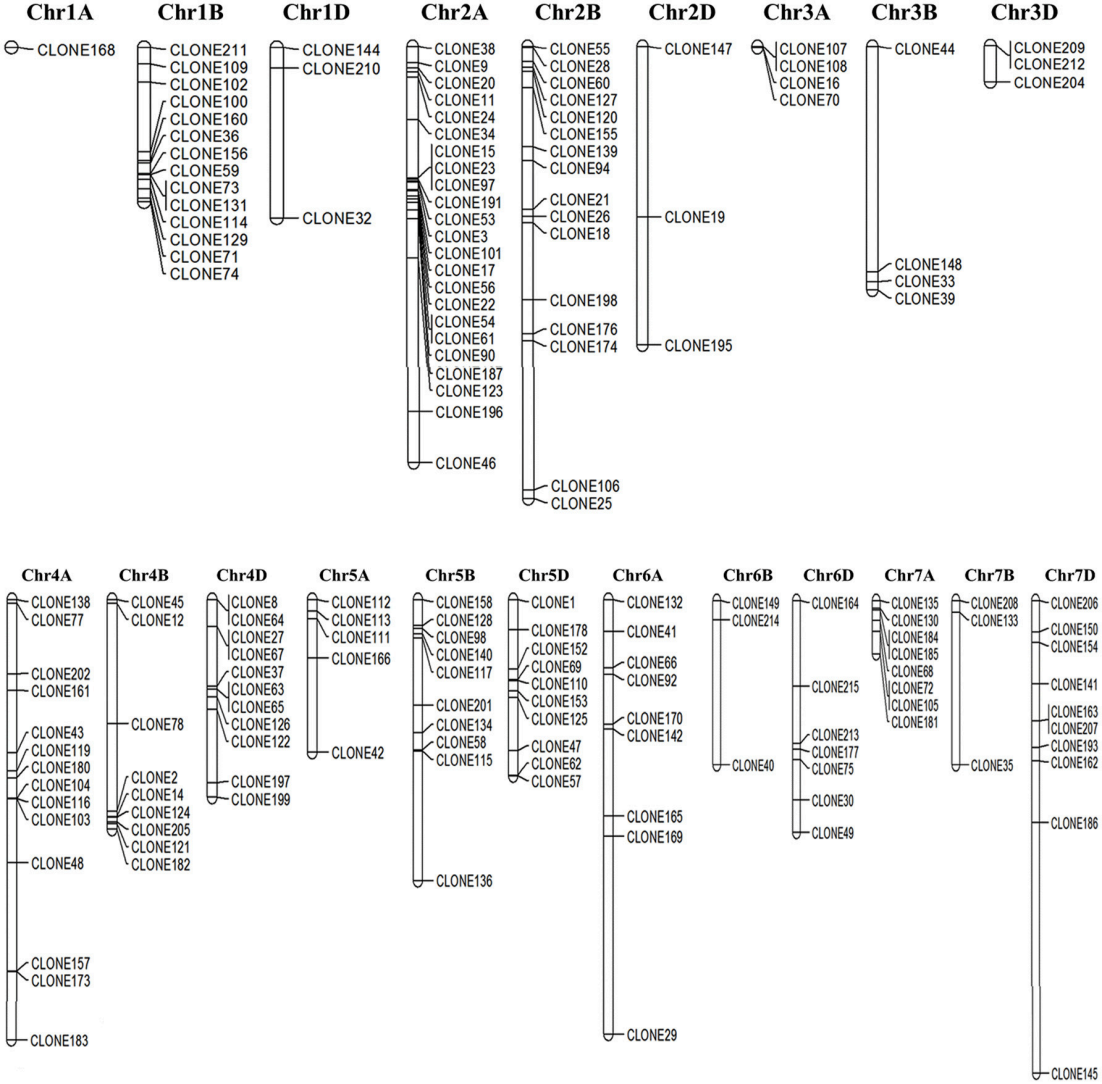
**Chromosomal localization of the cloned Expressed Sequence Tags (ESTs) mapped based on the draft genome sequence of *Triticum aestivum*; Genome sequence was downloaded from the Ensembl Plants database (http://plants.ensembl.org/)**.

Out of 205 cloned ESTs, 170 were successfully mapped on to the genome sequence of *Triticum* (Figure 3) Most of the DEGs were observed to be localized on the long arm of chromosome 2A followed by chr 2B, whereas very few were observed on chr 1A (Figure 3).

Some of the identified ESTs were closely linked and localized in the same locus on the chromosome. Domain based characterization showed the respective clones to code for different genes, for example clones 73 and 131 lie in the same locus on chr 1B ans they code for HSP70 (acc. no. tplb0015g23) and HSP90 (tplb0015g23; Table S2). Similarly, chr 2A harbor clones 15, 23, and 97 in the same locus, which domain-based characterization showed to representATPase-like (acc. no. tplb0017c09), HSP90 (acc. no. tplb0017c09), and HSP20 (acc. no. X64618_1). These genes showed complete linkage between each other preventing recombination, and hence presenting as desirable marker genes for screening wheat for thermotolerance.

### Post-translational modifications (PTMs) identified in the cloned ESTs

**Figure 4:**
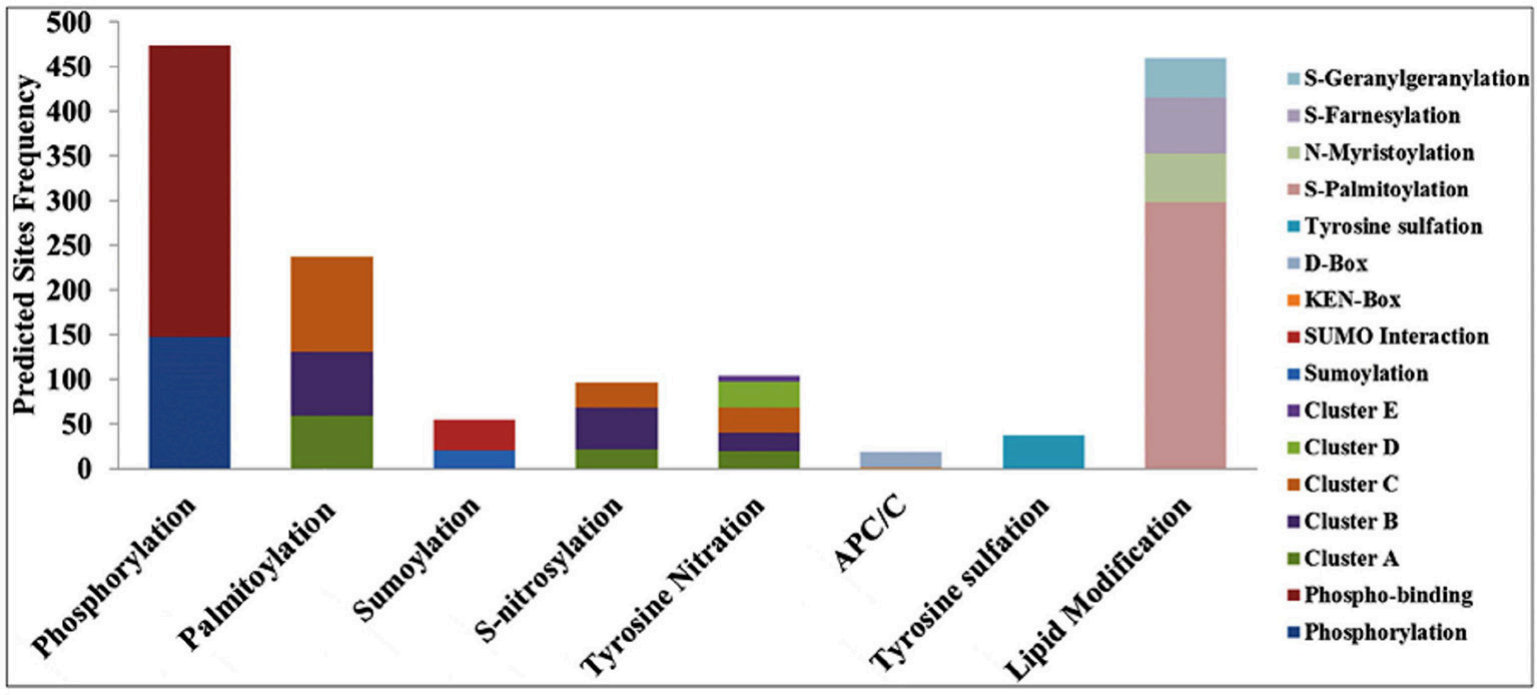
**Post-Translational Modifications (PTMs) predicted in the differentially expressed proteins (DEPs) corresponds to the cloned Expressed Sequence Tags (ESTs) from wheat *cv*. HD2985 under the HS**.

The DEPs predicted from the CDS (corresponds to the cloned ESTs) were used for the post-translational modifications (PTMs) characterization. Eight different types of PTMs were found in the DEP sequences—phosphorylation, palmitoylation, sumoylation, S-nitrosylation, tyrosine nitration, APC/C (anaphase-promoting complex or cyclosome), tyrosine sulfation, and lipid modifications (Figure 4). Predicted DEPs from ESTs contained a large number of potential sites for phosphorylation, an important attribute with highest frequency, and 147 phosphorylations, and 327 phosphobinding sites were predicted in 134 DEPs. Further, site-specific phosphorylation showed the presence of serine (129), threonine (46), and tyrosine (06) with scores >0.9.

We observed 21 sites with sumoylation (≤ 36.625), and 34 sites with sumoylation interaction (cut off 59.29) in 55 EST clones. Similarly, 237 palmitoylation (S-acylation) sites in 100 DEPs were observed, clustered in three groups: group A (59 sites), group B (71 sites), and group C (107 sites), on the basis of cut-off score. Ninety six S-nitrosylation sites were predicted in 71 DEPs, and sub-grouped into three clusters. Interestingly, we found a large number (3066) of calpain cleavage sites in 192 DEPs. In addition, 103 tyrosine nitration sites with five clusters were predicted in 70 DEPs, 19 APC/C (anaphase-promoting complex or cyclosome) sites were predicted in 17 DEPs and 37 tyrosine sulfation sites were predicted in 28 DEPs. PTM data for the lipid modification showed 460 sites in 152 DEPs.

### Prediction of transmembrane (TM) motifs

Sixteen transmembrane helixes (TMH) have been predicted in 12 DEPs (Table 2); most of the TMHs were localized at 40–60 residues positions in the predicted DEPs, with the length 18, 19 and most of them were of 22 amino acids. Protein corresponds to clone 9 had the most number (4) of helix domains.

**Table 2:** **Transmembrane helixes (TMH) identified in the cloned expressed sequence tags**.

**ESTs**	**Number of predicted TMHs**	**TMH_position**	**Length of the TMH**
CLONE_9	4	12–34, 54–76, 96–118, 122–144	22
CLONE_29	2	12–34, 39–61	22
CLONE_68	1	10, 29	19
CLONE_41	1	30–52	22
CLONE_205	1	102, 124	22
CLONE_204	1	10, 28	18
CLONE_169	1	35, 57	22
CLONE_152	1	43, 62	19
CLONE_151	1	78, 100	22
CLONE_150	1	28, 47	19
CLONE_13	1	12, 34	22
CLONE_111	1	26, 48	22

### Digital expression (DE) analysis of cloned ESTs

**Figure 5:**
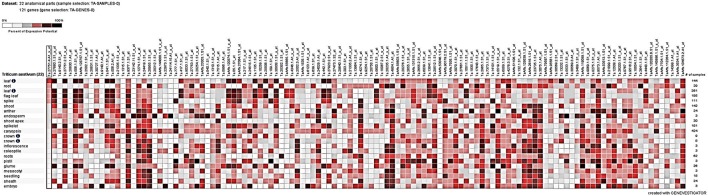
**Tissue-specific digital expression analysis of the cloned differentially expressed genes (DEGs) identified from the wheat *cv*. HD2985 by SSH library screening; Genevisible tool was used for the expression analysis**.

Tissue-specific expression analysis under different perturbations from Genevisible showed marked differential regulation of some of the target genes (Figure 5). Expressions of most of the DEGs were found relatively high in flag leaf, root, spikelet, glume, coleoptiles, and caryopsis tissues. Among the perturbations, more than 3.5-fold upregulation/downregulation was in response to abiotic stress (such as drought, cold, imbibition) and biotic stress (such as infection with *Blumeria graminis* sp., *Fusarium graminearum, Mayetiola destructor*). TC391278, TC381185, TC449352, and TC382164, in particular showed more than 4-fold up-regulation in response to cold and drought stresses, respectively; TC401047 and TC368608 were found down-regulated under cold and TC389965 in response to drought stress. We observed up-regulation of TC389965, TC382164, and TC449352 under HS.

Significant variations in the digital expression of 121 cloned genes (in present investigation) were observed in response to 45 different perturbations.

### Expression analysis of DEGs in wheat under heat stress

**Figure 6:**
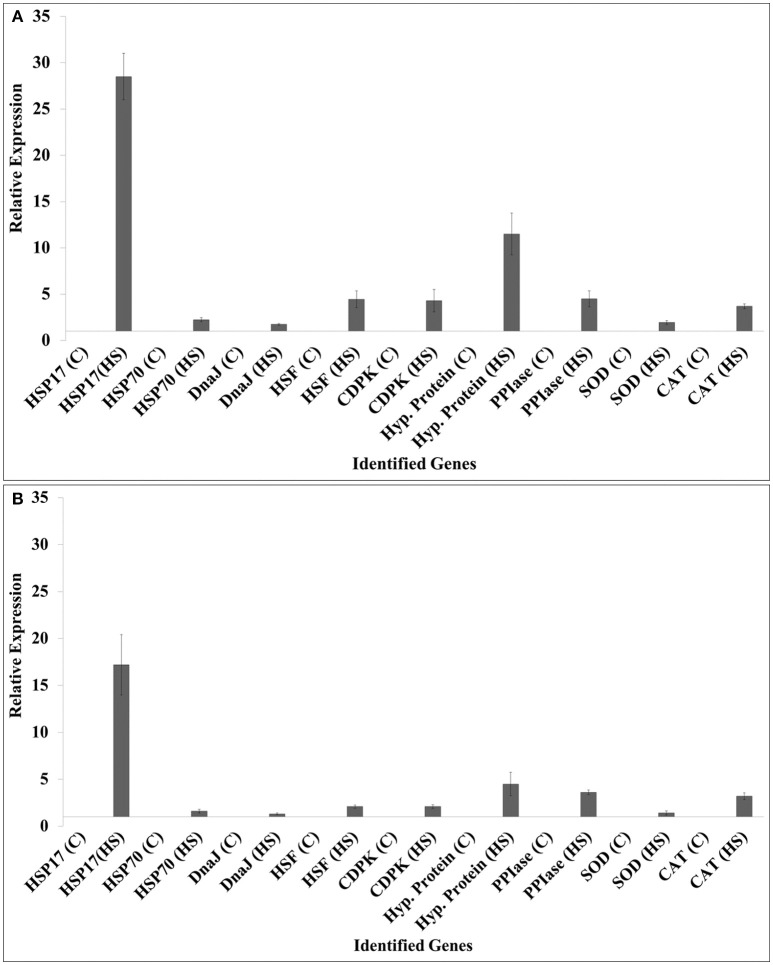
**Validation of randomly selected differentially expressed genes (DEGs) identified from the screening of FSH library using quantitative real-time PCR; contrasting wheat *cvs***. HD2985 and HD2329 were used for the expression analysis at the grain-filling stage; 9 randomly selected genes were used for the validation; ß-actin gene (accession no AB181991.1) was used as endogenous control gene for normalizing the Ct-value; Relative expression was calculated using the method of Pfaffl (2001).

We selected HSPs (*HSP17, HSP70*), *DnaJ, HSF*, calcium dependent protein kinase (*CDPK*), hypothetical protein, peptidyl propyl isomerase (*PPIase*), *SOD*, and catalase (*CAT*) for the expression analysis (Figure 6).

The expression of *HSP17* showed relatively high fold up-regulation, as compared with other selected genes in response to HS; HD2985 showed more transcripts compared to HD2329. Similarly, hypothetical protein gene showed 10-fold increase in the expression in HD2985, as compared to < 5-fold in HD2329 under HS. We observed significant up-regulation of other selected genes such as *HSP70, DnaJ, HSF, CDPK, PPIase, SOD*, and *CAT* in response to HS, and transcripts were more abundant in the thermotolerant (HD2985) than in the thermosusceptible (HD2329) cultivar.

**Figure 7:**
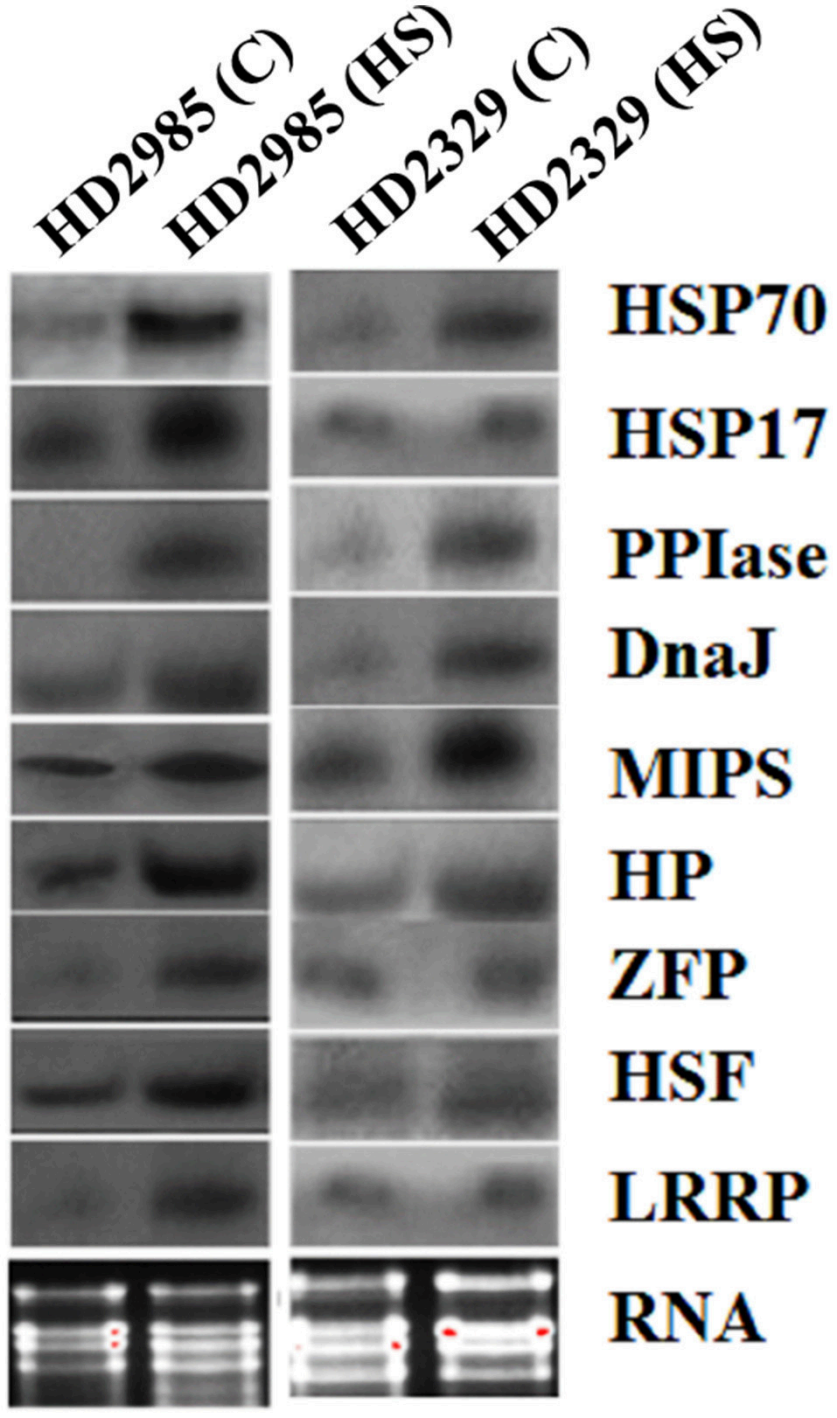
**Northern blot analysis of randomly selected differentially expressed genes (DEGs) identified from the screening of FSH library generated from the wheat endosperm; expression analysis was carried out in contrasting wheat cultivars (HD2985 and HD2329) under the heat stress; *HSP17*, *HSP70*, *DnaJ*, *HSF*, *CDPK*, hypothetical protein (HP), Zinc finger protein (ZFP), peptidyl propyl isomerase (*PPIase*), LRR like protein (LRRP) were used for the northern blotting**.

Northern blot analysis showed increase in the expression of the selected genes as visualized by the appearance of the prominent blot in case of *HSP17, HSP70, HSF, MIPS*, Hypothetical protein in HD2985, as compared to feeble expression in HD2329 (Figure 7). The results of northern blot analysis validate the findings of qRT-PCR. The transcripts of the selected genes were observed more in thermotolerant cultivar, as compared to thermosusceptible under HS.

## Discussion

Here, we constructed HS-responsive FSH libraries with the endospermic tissues of wheat *cv*. HD2985 with an objective to identify heat-responsive genes and proteins. While most of the ESTs were associated with stress responses (Kumar et al., 2014), some of them are predicted to play important role in signaling and metabolic pathways. Starch biosynthesis pathway is most affected under elevated temperature in wheat, as evident from the shriveled grains and low yield. The tolerance mechanism operating in wheat during grain-filling has yet not been elucidated (Kumar et al., 2013). ESTs and their corresponding DEPs identified in present investigation showed a large association with the tolerance of the plant under HS. The findings are in conformity with the observation of Kumar et al. (2014). The identified ESTs/DEPs can be used as desirable markers for screening wheat for thermotolerance.

PTM analysis of DEPs showed large number of phosphorylation sites. Phosphorylation sites undergo alternative splicing and contribute to the functional amendments (Zhang and Mount, 2009). Phosphorylation is known to influence RNA binding, splicing activity, subcellular localization, and protein-protein interactions (Zhang and Mount, 2009). The direct evidence of the functional role of phosphorylation in regulation of HS-response is provided by the studies of Evrard et al. (2013) on Arabidopsis; he reported activation of mitogen-activated protein kinase (MPK6) under HS which in turn target the heat stress transcription factor HsfA2.

Similarly, we observed sumoylation sites in the identified DEPs. Recently, sumoylation (Saracco et al., 2007) and phosphorylation sites (*In silico* identification of MAPK3/6 substrates in WRKY, bZIP, MYB, MYB-related, NAC and AP-2 transcription factor family) have been explored in *Arabidopsis*. Resident-protein post-translational modifications (PTM), such as sumoylation may be constituents of “quick reaction force” (QRF) effects; it can change the rate of activity, function or location of the modified protein. Sumoylation of proteins may act in the stress-signal transduction or in adaptive response to stress; modification of large number of proteins associated with HS-tolerance has been reported in Arabidopsis, Soybean [*Glycine max* (L.) Merr], Sorghum (*Sorghum bicolor*), Rice, Wheat, Maize (Raorane et al., 2013; Singh et al., 2014).

We also observed significant numbers of palmitoylation (S-acylation), S-nitrosylation, calpain cleavage, and tyrosine nitration sites in the predicted DEPs. Palmitoylation is reversible post-translational addition of acyl lipids to cysteine residues and is reported to be involved in the development and salt-tolerance mechanism of Arabidopsis (Hemsley et al., 2008). S-nitrosylation processes have been reported to regulate the energy transduction system in the chloroplast thylakoids (Lindermayr et al., 2005) and in the abiotic stresses (Romero-Puertas et al., 2013). Calpains constitute an important family of the Ca^2+^-dependent cysteine proteases, which contain a nucleophilic cysteine in the catalytically active site, highly conserved and with demonstrated role in the development of the aleuronic layer during the seed development in maize and *Arabidopsis thaliana* (Johnson et al., 2008).

Tyrosine nitration is mediated by reactive nitrogen species (RNS) that is linked to nitro-oxidative damage in plant cells (Corpas et al., 2013), as well as during the development and senescence. Similarly, anaphase-promoting complex or cyclosome sites were observed in the identified DEPs, predicted to be associated with cell-cycle-regulated ubiquitin-protein ligase (Capron et al., 2003). Tyrosine sulfation of the plant peptide PSY1 and phytosulfokine (PSK) have been implicated in growth and cell proliferation (Mahmood et al., 2014). Here, we identified lipid modifying sites in the predicted DEPs which are reported to be involved in plant disease resistance (Shah, 2005) and the developmental processes.To sum up, these PTM sites are predicted to be critical for stress-tolerance, developmental processes, and resistance to diseases; the sites located in the upstream, in particular of the PTM may be predicted to play important role in tolerance. Further, evaluation and validation is, however, required in this area.

The identified DEGs were randomly selected for the validation in wheat under HS through qRT-PCR. We observed maximum relative expression of *HSP17* in response to HS, as compared to other selected genes which is in conformity with the observation of DeRocher and Vierling (1994). Cytosolic small HSPs function as molecular chaperone, preventing the thermal aggregation of proteins, and facilitate their reactivation after the stress (Lee et al., 1997; Klueva et al., 2001). We identified heat-responsive TFs predicted to regulate the expression of SAGs. The finding is in conformity with the report of Scarpeci et al. (2008) who characterized HSFs involved in regulating the expression of genes associated with stress response in *Arabidopsis*. Heat shock elements (HSEs) located in the promoter region of HSPs is recognized by HSF, under the stress condition, and regulates the expression of HSPs at the transcriptional level (Krishna, 2004). Similarly, CDPKs are important calcium sensors in calcium-mediated signal transduction pathways. Relative fold expression of CDPK was higher in HD2985, as compared to HD2329 under HS which is in accordance with the report of Wang and Song (2014) who observed that overexpression of *ZmCK3* improved plant survival rate under the heat and drought stresses in the transgenic *Arabidopsis* plants. Alteration in the Ca^2+^ efflux trigger the activities of kinases and peroxidases, more in thermotolerant compared to thermosusceptible *cvs*., which in turn regulate the accumulation of ROS inside the cell, and attenuate the lipid peroxidation process (Goswami et al., 2015). Various reports have shown that in plants, the transcripts and protein levels of many ROS scavenging enzymes are elevated by HS (Secenji et al., 2010). The abundance of transcript of antioxidant enzymes such as SOD, CAT, and APX under HS during grain-filling stage suggests their potential role in modulating the defense system against the HS.

We identified very specific domains in the sequence of DEGs, including CS, AAA, PsbP, and CP12. The presence of CS domain suggest involvement of the respective gene in recruiting HSPs to multi-protein assembly (Lee et al., 2004). The AAA domain has a chaperone-like function (Koonin et al., 2004). The PsbP domain is present in the oxygen-evolving system of photosystem II and increases the affinity of water oxidation site for Cl^−^ and provide the conditions required for the high affinity binding of calcium ion (Kochhar et al., 1996). The CP12 domain, having three conserved cysteine and a histidine, seems to be zinc-finger domain, and the LSD1-like zinc-finger domains monitor a superoxide-dependent signaling and negatively regulate the plant cell death pathway (He et al., 2011). The LRR motif protein has a role as a signaling molecule involved in the HS-response (Padaria et al., 2013) and an EF hand-having helix-loop-helix structural domain is found in the calcium binding protein. Mosser et al. (1990) have reported that changes in calcium ion concentration also affect the binding activity of heat stress transcription factors (HSFs) to the heat shock elements (HSE). Saidi et al. (2009) found that a calcium permeable channel in the plasma membrane activated by HS initiates synthesis of HS-related proteins, especially HSPs. PPIase expression was significantly high in wheat under HS; FKBP and FK506 binding proteins have peptidyl-prolyl cis-trans isomerase (PPIase) activity, and function as protein-folding chaperones, for the proteins expressed under the stress; and it contains proline residues (Göthel et al., 1996). Similarly, in plants, CyPA (cyclophilin A) is involved in signal transduction mechanism of the regulation in response to various abiotic stresses, *via* phosphoprotein cascade, Ca^2+^ and other secondary signaling molecules (Xiong et al., 2002). Expression analysis of peptidyl propyl isomerase studied here shows its higher expression under HS, in conformity with the findings of Kurek et al. (1999). PPIase are known to protect plants from the abiotic stresses, and a heat-inducible FKBP77 (encoding for PPIase) shown to be expressed in wheat roots (Geisler and Bailly, 2007).

We discovered transmembrane helices in twelve of the DEPs. The length of TMHs can regulate the partitioning of single TM proteins in different lipid sub-domains of endoplasmic reticulum (ER) and their export to the Golgi apparatus (Ronchi et al., 2008). The IRE 1 protein, containing several transmembrane helices, has been associated with HS sensing by other researchers in the Arabidopsis. IRE 1 is a transmembrane serine/threonine-protein kinase/endoribonuclease that transmits the UPR (unfolded protein response) signal across the endoplasmic reticulum or nuclear membranes (Mittler et al., 2012).

## Conclusion

We constructed HS-responsive FSH libraries from the endosperm tissue of wheat and identified more than 500 ESTs; 205 of them were cloned and characterized to be associated with photosynthesis and protein folding. Some of the identified ESTs, such as ATPase, HSP90 and HSP20 lying on chr 2A showed complete linkage between each other, and hence predicted to be desirable marker for screening wheat for thermotolerance. Eight different post-translation modifications were observed in the predicted DEPs. PTMs were observed to regulate the signal transduction, tolerance network, and metabolic associated pathways through changes in the expression of DEPs. Most of the identified DEGs were abundantly observed in the flag leaf, root, spikelet, glume, coleoptiles, and caryopsis tissues of wheat. Expression analysis showed greater abundance of transcripts in the thermotolerant than in the thermosusceptible cultivar under HS. Information regarding the PTMs can be used to manipulate the expression of DEGs/ DEPs associated with thermotolerance as well as pathway elucidation; it will accelerate the pace of breeding program for the development of “climate-smart” wheat.

## Author contributions

SG and RK conceived and designed the experiments. KD, JS, ST, YK, and AK performed the heat stress treatment, sample collection, library preparation, real time PCR, and Northern blotting. SS, DM, SK, MG, and AR performed the data analysis. JP, GS, HP, VC, AR contributed reagents/materials/analysis tools. SG, RK, SP, VC, AR, and RR wrote the manuscript; all authors contributed to the discussion and approved the final manuscript.

### Conflict of interest statement

The authors declare that the research was conducted in the absence of any commercial or financial relationships that could be construed as a potential conflict of interest.

## Supplementary material

Table S1 **List of primers used for the validation of randomly selected genes in contrasting wheat *cvs*. by quantitative Real-Time PCR**.

Table S2 **Nucleotide sequence of identified and cloned ESTs from wheat *cv*. HD2985 by screening SSH library; Sanger's di-deoxy method was used for the sequencing**.

Supplementary File **Gene Ontology (GO) enrichment analysis of the cloned Expressed Sequence Tags (ESTs) identified from the screening of FSH libraries generated from the wheat *cv*. HD2985; Singular Enrichment Analysis (SEA) tool was used; putative conserved domain were also predicted using the Pfam and InterProScan 5 databases**.
