# Supplementary material for: SSH Analysis of Endosperm Transcripts and Characterization of Heat Stress Regulated Expressed Sequence Tags in Bread Wheat
Source: Front Plant Sci. 2016 Aug 17;7:1230. doi: 10.3389/fpls.2016.01230 (PMC4988357; doi:10.3389/fpls.2016.01230)
Supplement: Table S1 — List of primers used for the validation of randomly selected genes in contrasting wheat cvs. by quantitative Real-Time PCR. [file Table1.docx]

**Supplementary Table S1. List of primer sequences for qRT-PCR**

| **Gene Name** | **Primer Sequence** | **Tm (°C)** |
| --- | --- | --- |
| HSP70 (F) | CTTCGTCCAGGAGTTCAAGC | 60.5 |
| HSP70 (R) | GTCGATCTCGATGGTGGTTT | 58.4 |
| HSP17 (F) | AGTGGGTAGCGAGTTTCCTGTGAT | 57.4 |
| HSP17 (R) | CAAACAACCACCAGTACGCACGAA | 57.4 |
| DnaJ (F) | CAGGTGGTGCAGTTGGTGTAATTTCAG | 59.7 |
| DnaJ (R) | GCAGAGCAACGGGCACATGGCA | 60.4 |
| Seed Specific HSF (F) | ATCCAGCAACAAGATAAACTGA | 56.4 |
| Seed Specific HSF (R) | ATTCCCTGTCCTCAAATATGAA | 56.4 |
| CDPK (F) | GCGGCTGATACAGACACTGATGTAAC | 59.5 |
| CDPK (R) | CTGTGATATAGCCACTGCCATCTTTGTC | 59.9 |
| Hypothetical Protein (F) | CTAGGGCGTGAAGTGACATCCTCA | 59.1 |
| Hypothetical Protein (R) | ATGCTGCCGGCAGCTAGCACAGAT | 60.8 |
| PPIase (F) | GATCCTGAAAACAGGGATGTGA | 60.1 |
| PPIase (R) | CTGGCCATAGGCTGCGCTTCCT | 60.4 |
| SOD (F) | TCCTTTGACTGGCCCTAATG | 58.4 |
| SOD (R) | CTTCCACCAGCATTTCCAGT | 58.4 |
| CAT (F) | CAAGAGCGATTCATCAACAGAT | 58.4 |
| CAT (R) | AGACCAGTAGGAGAGCCAGATG | 56.7 |
| β-Act (F) | GCGGTCGAACAACTGGTATT | 54.4 |
| β-Act (R) | GGTCCAAACGAAGGATAGCA | 54.8 |
