# Supplementary material for: SSH Analysis of Endosperm Transcripts and Characterization of Heat Stress Regulated Expressed Sequence Tags in Bread Wheat
Source: Front Plant Sci. 2016 Aug 17;7:1230. doi: 10.3389/fpls.2016.01230 (PMC4988357; doi:10.3389/fpls.2016.01230)
Supplement: Table S2 — Nucleotide sequence of identified and cloned ESTs from wheat cv. HD2985 by screening SSH library; Sanger's di-deoxy method was used for the sequencing. [file Table2.docx]

**Supplementary Table S2. Nucleotide sequence of identified and cloned expressed sequence tags from wheat *cv.* HD2985 by screening SSH library**

>CLONE_1

CGGCGTCAAGTTCGGCGAGGCCGTGTGGTTCAAGGCCGGCTCCCAGATCTTCAGCGAGGGCGGCCTCGACTACCTCGGCAACCCCAGCCTGGTGCACGCGCAGAGCATCCTCGCCATCTGGGCCTGCCAGGTCGTGCTCATGGGCGCCGTCGAGGGCTACCGCGTCGCCGGCGGCCCGCTCGGCGAGATCGTCGACCCGCTCTACCCCGGCGGCAGCTTCGACCCCCTGGTCCTCGCCGACGACCCCGAGGCCTTCGCGGAGCTCAAGGTGAAGGAGATCAAGAACGGCCGCCTCGCCATGATCTCCATGTTCGGCTTCTTCGTGCAGGCCATC

>CLONE_2

CATCTTGGGTATCTGGGGAGGCAAGGGTCAAGGAAAATCCTTCCAGTGTGAGCTTGTCTTCGCCAAGATGGGCATCAACCCAATCATGATGAGTGCCGGAGAGCTGGAGAGTGGCAACGCCGGAGAGCCAGCCAAGCTCATCAGGCAGCGGTACCGTGAGGCTGCAGACATGATCAAGAAGGGTAAGATGTGCTGCCTCTTCATCAACGATCTTGACGCCGGTGCGGGTCGGATGGGCGGGACCACACAGTACACCGTCAACAACCAGATGGTGAACGCCACCCTCATGAACATCGCCGATGCCCCCACCAACGTGCAGCTCCCAGGCATGTACAACAAGGAGGAGAACCCTCGTGTGCCCATCGTCGTCACTGGTAACGATTTCTCGACGT

>CLONE_3

CGGAGGGCGAGCGGCTCGGGCGCGGCACCAAGATCACGCTCTTCCTCAAGGACGACCAGCTCGAGTACCTGGAGGAGCGCCGCCTCAAGGACCTCGTCAAGAAGCACTCGGAGTTCATCAGCTACCCCATCTACCTCTGGACCGAGAAGACCACCGAGAAGGAGATCAGCGACGACGAAGACGAAGACGCTTCCGAGGAGAAGAAGGAAGGCGAGGTCGAGGAGGTCGACGATGACAAGGACAAGGACGAGAGCAAGAAGAAGACGAAGAAGGTGAAGGAGGTGAGCCACGAGTGGGCGCAGATCAACAAGCAGAAGCCCATCTGGCTGCGCAAGCCGGAGGAGATCAGCAAGGAGGAGTACGCGTCCTTCTACAAGAGCATCACCAACGACTGGGAGGATCACCTGGCCGTGAAGCACTTCTCGGTGGAGGGGCAGCTGGAGTTCAAGGCGGTGCTCTTCGT

>CLONE_4

ATGGCCGCGTCGGCGCTGCACCAGACGACCAGCTTCCTCGGCACCGCCCCGCGGCGTGATGACCTCGTCCGCAGCGTCGGCGACTTCGGCGGCCGCATCACCATGCGCCGGACCGTCAAGAGCGCGCCCCAGAGCATCTGGTACGGCCCTGACCGTCCCAAGTACCTGGGCCCGTTCTCCGAGCAGACTCCATCGTACCTGACCGGCGAGTTCCCCGGCGACTACGGCTACCTCACCGGCGAGTTCCCCGGCGACTACGGCTGGGACACCGCGGGGCTCTCGGCCGACCCCGAGACCTTCGCCAAGAACCGGGAGCTGGAGGTGATCCACTGCCGGTGGGCCATGCTGGGCGCGCTCGGCTGCGTCTTCCCGGAGCTGCTCGCCCGCAA

>CLONE_5

GAACTTCATGACCCTGCCCAACATCAAGATCCCACTCATCTTGGGTATCTGGGGAGGCAAGGGTCAAGGAAAATCCTTCCAGTGTGAGCTTGTCTTCGCCAAGATGGGCATCAACCCAATCATGATGAGTGCCGGAGAGCTGGAGAGTGGCAACGCCGGAGAGCCAGCCAAGCTCATCAGGCAGCGGTACCGTGAGGCTGCAGACATGATCAAGAAGGGTAAGATGTGCTGCCTCTTCATCAACGATCTTGACGCCGGTGCGGGTCGGATGGGCGGGACCACACAGTACACCGTCAACAACCAGATGGTGAACGCCACCCTCATGAACATCGCCGATGCCCCCACCAACGTGCAGCTCCCAGGCATGTACAA

>CLONE_6

ATGTCGATCGTGCGGCGGAGCAACGTGTTCGACCCCTTCGCCGACCTCTGGGCGGACCCCTTCGACACCTTCCGCTCCATCGTCCCGGCGATCTCAGGCGGCAGCAATGAGACAGCTGCGTTCGCGAACGCCCGGATGGACTGGAAGGAGACGCCTGAGGCACACGTCTTCAAGGCCGACCTCCCCGGCGTGAAGAAGGAGGAGGTCAAGGTGGAGGTGGATGACGGCAACGTGCTCGTCGTCAGCGGCGAGCGCACAAAGGAGAAGGAGGACAAGAACGATGATCTTCCAGGTGTGAAAAAGGAGGAGGTCAAGGTGG

>CLONE_7

TGTACGCCCCTCTGATCCGTGATGGTCGTATGGAGAGGGTATCTTCCAGACCGAACAATGTCAGCGACGAGTCCGTCGTCAAGATCGTCGACACCTTCCCAGGACAATCCATCGACTTTTTCGGTGCTCTGCGTGCTCGGGTGTACGACGACGAGTCGAGAAGCTGCCTAGAGTACGGGCACATGCTGGTCCAGGAGCAGGACAATGTCAAGCGTGTGCAGCTTGCTGACACCTACATGAGCCAGGCAGCTCTGGGTGATGCTAACCAGGATGCGATGAAGACTGGTTCCTTCTACGGAGGAGGTAAAGGGGCACAGCAAGGTACTTTGCCTGTGCCGGCAGGATGCACCGACCAGACTGCCAAGAACTTCGACCCAACGGCGAGGAGTGACGACGGCAGCTGCCTTTACACCTTTTAA

>CLONE_8

ATGGCGTCTGCTGCTCTCCTCAAATCGTCTTTCCTTCCCAAGAAGGCCGAATGGGGTGCCACGCGCCAGGCCGCCGCCGCCAAGCCGGTGACCGTCTCCATGGTTGTCCGTGCCAGCGCGTACGCCGATGAGCTTGTCAAAACCGCGAAAACCATCGCATCGCCAGGCAGGGGTATCCTTGCCATGGATGAGTCGAATGCCACCTGTGGCAAGAGACTCGCCTCGATTGGCCTTGAGAACACCGAGGCTAACCGCCAGGCTTACCGGACCCTTCTTGTCACTCCACCAGGATTGGGAAACTACATCTCTGGTGCTATCCTCTTTGAGGAGACCCTCTACCAGTCGACTGTTGATGGCAAGAAGATTGTT

>CLONE_9

ATGACTGCAATTTTAGAGAGACGCGAAAGTACAAGCCTGTGGGGTCGCTTCTGCAACTGGATAACTAGCACTGAAAATCGTCTTTACATCGGATGGTTCGGTGTTTTGATGATCCCTACCTTATTGACCGCAACTTCTGTATTTATTATCGCCTTCATCGCTGCCCCTCCAGTAGATATTGATGGTATTCGTGAGCCTGTTTCTGGTTCTTTACTTTATGGAAACAATATTATCTCTGGTGCTATTATCCCTACTTCTGCGGCGATCGGATTGCACTTTTACCCAATTTGGGAAGCTGCATCTGTTGATGAGTGGTTATACAATGGTGGTCCTTATGAGCTAATTGTTCTACACTTCTTACTTGGTGTAGCTTGTTATATGGGTCGTGAGTGGGAACTTAGTTTCCGTCTGGGTATGCGTCCTTGGATTGCTGTTGCATATTCAGCTCCTGTTGCAG

>CLONE_10

ATGGCTGCTGCCTTCTCCTCCACCGTCGGTGCCCCGGCTTCTACGCCGACCAACTTCCTCGGGAAGAAGCTCAAGAAGCAGGTGACCTCGGCCGTGAACTACCATGGCATGAGCTCCAAGGCCAACAGGTTCACAGTCATGGCAGCGGAAAACATCGACGAGAAGAGGAACACGGACAAGTGGAAGGGTCTTGCGTACGATATCTCCGATGACCAGCAGGACATCACCAGAGGGAAGGGCATCGTGGACTCCCTCTTCCAGGCGCCCACGGGCGACGGCACCCAGGAGGCCGTCCTCAGCTCCTACGAGTACGTCAGCCAGGGACTCAAGAAGTACGACTTCGACAACACCATGGGAGGCTTCTACATCGCTCCTGCTTTCATGGACAAGCTTGTTGTCCATCTCTCCAAGAACTTCATGACCCTGCCCAACATCAAGATCCCACT

>CLONE_11

CTGCTACTGCTGTTTTCTTGATTTACCCTATTGGTCAAGGAAGCTTTTCTGATGGTATGCCTTTAGGAATCTCTGGTACTTTCAACTTTATGATTGTATTCCAGGCAGAGCACAACATCCTTATGCATCCATTCCACATGTTAGGTGTAGCTGGTGTATTCGGCGGTTCCCTATTCAGTGCTATGCATGGTTCCTTGGTAACCTCTAGTTTGATCAGGGAAACTACTGAAAATGAATCTGCTAATGAGGGTTACAAATTTGGTCAAGAGGAAGAAACTTATAATATTGTGGCTGCTCATGGTTATTTTGGCCGATTAATCTTCCAATATGCTAG

>CLONE_12

ATGAGCTCCAAGGCCAACAGGTTCACAGTCATGGCAGCGGAAAACATCGACGAGAAGAGGAACACGGACAAGTGGAAGGGTCTTGCGTACGATATCTCCGATGACCAGCAGGACATCACCAGAGGGAAGGGCATCGTGGACTCCCTCTTCCAGGCGCCCACGGGCGACGGCACCCAGGAGGCCGTCCTCAGCTCCTACGAGTACGTCAGCCAGGGACTCAAGAAGTACGACTTCGACAACACCATGGGAGGCTTCTACATCGCTCCTGCTTTCATGGACAAGCTTGTTGTCCATCTCTCCAA

>CLONE_13

TTTCAACAACTCTCGTTCTTTACACTTCTTCTTGGCTGCTTGGCCTGTAGTAGGAATCTGGTTCACTGCTTTAGGTATTAGTACTATGGCTTTCAACCTAAATGGTTTCAATTTCAACCAATCTGTAGTTGATAGTCAAGGTCGCGTTATTAATACTTGGGCTGATATCATCAACCGTGCTAACCTTGGTATGGAAGTAATGCACGAACGTAATGCTCACAACTTCCCTCTAGACTTAGCTGCTGTTGAAGTTCCATCTATTAATGGATAA

>CLONE_14

CAAGGAGGAGAACCCTCGTGTGCCCATCGTCGTCACTGGTAACGATTTCTCGACGTTGTACGCCCCTCTGATCCGTGATGGTCGTATGGAGAGGGTATCTTCCAGACCGAACAATGTCAGCGACGAGTCCGTCGTCAAGATCGTCGACACCTTCCCAGGACAATCCATCGACTTTTTCGGTGCTCTGCGTGCTCGGGTGTACGACGACGAGTCGAGAAGCTGCCTAGAGTACGGGCACATGCTGGTCCAGGAGCAGGACAATGTCAAGCGTGTGCAGCTTGCTGACACCTACATGAGCCAGGCAGC

>CLONE_15

GAGATCTTCCTCCGCGAGCTCATCTCCAACTCCTCAGATGCGCTTGACAAGATCCGGTTCGAGAGCCTCACCGACAAGAGCAAGCTGGACGCGCAGCCGGAGCTCTTCATCCGCCTCGTCCCCGACAAGGCCACCAAGACGCTCTCCATCATCGACAGCGGCGTCGGCATGACAAAGTCAGACCTTGTGAACAACCTGGGCACCATCGCGCGCTCCGGCACCAAGGAGTTCATGGAGGCGCTTCAGGCGGGCGCGGACGTGAGCATGATCGGCCAGTTCGGGGTCGGCTTCTACTCGGCCTACCTCGTCGCCGACAAGGTGGTGGTCACCACCAAGCACAACGACGACGAGCAGTACGTGTGGGAGTCGCAGGCCGGCGGCTCCTTCACCGTCACCCTCGACA

>CLONE_16

AGGTGGAGGACGGCAATGTGCTCGTCGTCAGCAGCGAGCGCACAAAGGAGAAGGAGGACAAGAACGACAAGTGGCACCGCGTTGAGCGCAGCAGCGGCAAGTTCGTCAGGCGCTTCCGCCTGCCGGAGGACGCCAAGGTGGAGGAGGTGAAGGCCAGGCTGGAGAACGGGGTGCTCACCGTCACCGTGCCCAAGGCCCACGTCAAGAAGCCCGAGGTGAAGGCCATCGAGATATCCGGCTGA

>CLONE_17

GCCCCGCCGCGCGCCCTTCGACCTCTTCGACACCCGCAAGAAGATGAACAACATCAAGCTCTACGTGCGCCGCGTCTTCATCATGGACAACTGCGAGGAGCTCATCCCGGAGTGGCTGGGCTTCGTCAAGGGCGTCGTCGACTCGGACGACCTCCCCCTCAACATCTCCCGCGAGACGCTGCAGCAGAACAAGATCCTCAAGGTCATCCGCAAGAACCTCGTCAAGAAGTGCATCGAGCTCTTCTTCGAGATCGCCGAGAACAAGGAGGACTACGCCAAGTTCTACGAGGCATTCTCCAAGAACCTCAAGCTGGGCGTGCACGAGGACTCCCAGAACCGAGCCAAGCTCGCCGACCTGCTCCGGTATCACTCGACCAAGAGCGGCGACGAGCTCACCAGCCTCAAGGACTACGTCAC

>CLONE_18

GAGCTGGACCCGGAGAGGAGGCTCTACCCCGGTGGCTCCTACTTCGACCCGCTGGGTCTCGCGGCGGACCCGGAGAAGAAGGAGACTCTGCAGCTGGCGGAGATCAAGCACGCCCGCCTCGCCATGGTCGCCTTCCTCGGCTTCGCCGTGCAGGCCGCCGCCACCGGCAAGGGGCCACTCAACAACTGGGCCACCCACCCCAGCGACCCGCTGCACACCACCATCTTCGACACCTTGGGTTCCTCTTAA

>CLONE_19

CGGATCATGAAGGCGCAGGCGCTGAGGGACAGCAGCATGGGCGCATACATGTCCTCCAAAAAGACCATGGAGATCAACCCGGAGAACGGCATCATGGAGGAGCTCCGCAAGCGCGCCGACGCCGACAAGAACGACAAGTCCGTCAAGGACCTCGTGATGCTGCTCTTCGAGACGGCGCTGCTCACCTCCGGGTTCAGCCTCGACGACCCCAACACCTTCGCCGCCAGGATCCACCGCATGCTCAAGCTCGGGCTCAACATCGATGATCAGGCCGATGCGGAGGAGGAGGATGCTGACATGCCCGCGCTGGAGGAGGAGGGCGCCGAGGAGAGCAAGATGGAGGAGGTCGACTGA

>CLONE_20

GAAGGCCCGCTCGAGGTCGGCGCCGACGGCACCCTCAAGTTCGAGGAGAAGGACGGCATCGACTACGCGGCCGTCACCGTGCAGCTCCCCGGAGGCGAGCGCGTGCCCTTCCTCTTCACCGTCAAGCAGCTCGTCGCCACCGGCAAGCCCGAGAGCTTCAGCGGGCCCTTCCTTGTGCCCAGCTACAGGGGTTCGTCTTTCCTCGACCCAAAGGGCCGTGGTGGCTCTACTGGCTACGACAACGCCGTGGCGCTACCCGCCGGAGGCAGAGGAGACGAGGAGGAGTTGGCCAAGGAGAACGTGAAGAACGCGTCCTCCTCCACGGGCAACATCACGCTCAGCGTCACCAAGAGCAAGCCGGAGACCGGCGAGGTGATCGGCGTCTTCGAGAGCGTGCAGCCGTCAGACACCGACCTCGGCGCCAAGGCGCCCAAGGATGTCAAGATCCAGGGTGTGTGGTACGCGCAGCTCGAGTCTAACTAG

>CLONE_21

ATGGCTTCGTCGGTGGCCGCCGCGGCGAGCGCGTTCCTCGGGACCCGGCTGGCGGACCCGGCGCCGCAGAACGGGCGCATCGTGGCGCGGTTCGGCTTCGGCAAGAAGAAGGCCCCGCCGAAGAAGGCCAAGGCGCCCCCGACCACCGACCGGCCGCTGTGGTTCCCCGGCGCGCAGGCGCCCGAGTACCTCGACGGCACGCTCGTCGGCGACTACGGCTTCGACCCCTTCGGCCTCGGGAAGCCCGCGGAGTACCTGCAGTACGACGTGGACTCCCTGGACCAGAACCTGGCCCAGAACCTGGCGGGCGAGATCATCGGCACCCGGTTCGAGGACGCCGACGTCAAGTCCACCC

>CLONE_22

CCGGATGAAGGAGGGCCAGAAGGACATCTACTACATCACCGGCGAGAGCCGCAAGGCCGTGGAGAACTCGCCCTTCCTGGAGCGCCTCAAGAAGCGCGGCTACGAGGTGCTCTTCATGGTGGACGCCATCGACGAGTACGCCGTGGGGCAGCTCAAGGAGTACGACGGCAAGAAGCTCGTCTCCGCCACCAAGGAGGGCCTCAAGCTCGACGAGGAGACCGAGGAGGAGAAGAAGCGCAAGGAAGAAAAAAAGGCGGCGTTCGAGGGCCTCTGCAAGATCATCAAGGACATCCTGGGCGACAAGGTGGAGAAGGTGGTCGTATCCGACCGCATCGTGGACTCGCCGTGCTGCCTGGTGACCGGCGAGTACGGGTGGACGGCCAACATGGAG

>CLONE_23

GAGATCTTCCTCCGCGAGCTCATCTCCAACTCCTCAGATGCGCTTGACAAGATCCGGTTCGAGAGCCTCACCGACAAGAGCAAGCTGGACGCGCAGCCGGAGCTCTTCATCCGCCTCGTCCCCGACAAGGCCACCAAGACGCTCTCCATCATCGACAGCGGCGTCGGCATGACAAAGTCAGACCTTGTGAACAACCTGGGCACCATCGCGCGCTCCGGCACCAAGGAGTTCATGGAGGCGCTTCAGGCGGGCGCGGACGTGAGCATGATCGGCCAGTTCGGGGTCGGCTTCTACTCGGCCTACCTCGTCGCCGACAAGGTGGTGGTCACCACCAAGCACAACGACGACGAGCAGT

>CLONE_24

ATGGCCGGCTTCGCCCTCGCCACCTCTGCCCTCCTCGTCTCCGGCGCGAGCGCGGAGGGGGCGCCCAAGAGGCTGACCTTCGACGAGATCCAGAGCAAGACCTACATGGAGGTGAAGGGTACCGGCACCGCGAACCAGTGCCCGACCATCGACGGCGGCGTCGACTCCTTCCCCTTCAAGGCCGGCAAGTACGAGATGAAGAAGTTCTGCCTGGAGCCCACCTCCTTCACCGTCAAGGCCGAGGGCATCCAGAAGAACGAGCCGCCGGCCTTCCAGAAGACCAAGCTCATGACCCGTCTCACCTACACCCTTGACGAGATG

>CLONE_25

CAAGCAGAAGCCCATCTGGCTGCGCAAGCCGGAGGAGATCAGCAAGGAGGAGTACGCGTCCTTCTACAAGAGCATCACCAACGACTGGGAGGATCACCTGGCCGTGAAGCACTTCTCGGTGGAGGGGCAGCTGGAGTTCAAGGCGGTGCTCTTCGTGCCCCGCCGCGCGCCCTTCGACCTCTTCGACACCCGCAAGAAGATGAACAACATCAAGCTCTACGTGCGCCGCGTCTTCATCATGGACAACTGCGAGGAGCTCATCCCGGAGTGGCTGGGCTTCGTCAAGGGCGTCGTCGACTCGGACGACCTCCCCCTCAACATCTCCCGCGAGACGCTGCAGCAGAACAAGATCCTCAAGGTCATCCGCAAGAACCTCGTCAAGAAGTGCATCGAGCTCTTCTTCGAGATCGCCGAGAACAAGGAGGA

>CLONE_26

CGTTCCAGCCCTACGCCGAGGTGTTCGGCCTCCAGAGGTTCCGCGAGTGCGAGCTCATCCACGGCCGCTGGGCCATGCTCGCCACCCTCGGCGCCCTCACCGTCGAGTGGCTCACCGGCGTCACCTGGCAGGACGCCGGCAAGGTGGAGCTGGTGGACGGGTCGTCGTACCTGGGGCAGCCGCTGCCGTTCACGCTCACCACGCTGATCTGGATCGAGGTGCTGGTGATCGGGTACATCGAGTTCCAGCGCAACGCG

>CLONE_27

ATGGTGACCCCTGGTGCTGAGTGCAAGGACAGGGCCACCCCTGAGGAAGTAGCCAGCTACACCCTCAAGCTTCTCCAGAGAAGGATCCCCCCTTCCGTCCCCGGCATCATGTTCTTGTCTGGCGGTCAGTCGGAGGTGGAGGCGACGCTGAACCTGAACGCGATGAACCAGGCGCCGAACCCATGGCACGTGTCCTTCTCCTACGCGCGGGCGCTGCAGAACACCTGCCTCAAGACGTGGGGCGGGCGGCCGGAGAACGTGGCCGCGGCGCAGGAGGCGCTGCTGCTGCGCGCCAAGGCCAACTCCCTGGCGCAGCTCGGCAAGTACACCAGCGACGGCGAGGCCGCGGAAGCCAGCGAGAACATGTTCGTCAAGAACTACAGCTACTGA

>CLONE_28

ACATCCTGGGCGACAAGGTGGAGAAGGTGGTCGTATCCGACCGCATCGTGGACTCGCCGTGCTGCCTGGTGACCGGCGAGTACGGGTGGACGGCCAACATGGAGCGGATCATGAAGGCGCAGGCGCTGAGGGACAGCAGCATGGGCGCATACATGTCCTCCAAAAAGACCATGGAGATCAACCCGGAGAACGGCATCATGGAGGAGCTCCGCAAGCGCGCCGACGCCGACAAGAACGACAAGTCCGTCAAGGACCTCGTGATGCTGCTCTTCGAGACGGCGCTGCTCACCTCCGGGTTCAGCCTCGACGACCCCAACACCTTCGCCGCCAGGATCCACCGCATGCTCAAGCTCGGGCTCAACATCGACGATTCCGCCGATGGCGCGGAGGAGGAGGACGCCGACATGGCGGCGCTGGAGGAGGAGGGTGCCGAGGAGAGCAAGATGGAGGAGGTGGACTGA

>CLONE_29

GCCAAGGTATGCCTCAGCAGTCTCCTTCATCTTTCCAAGAATCATAGCACTGATCTCCTCAGGGCTGA

AGACCTTGGTCTCTCCATCCTTGATCTTGACCTGAATGTAAGGCTTCCCCTCCTTGTTCACAATCTTGTAGGGGACAAGTTTCATGTCCCTCTGGACCTCCTTGTCCTCAAACTTTCTCCCAATGAGACGCTTGACGTC

AAAGACGGTCCTCTCAGGGTTGACAGCCGCCTGGTTCTTGGCAGCCTCACCGATGAGCCTCTCCCCATC

GGTGAACCCAACCCATGAGGGCGTGATACGGTTACCCT

>CLONE_30

ATGGCGGTCGAGTATGGTGCAAGGAGCCATAACAGTGGTCCTTGGAGTTGGTGCGATCCGGCGACGGGCTACAAGGTGAGCGCACTCACGGGCTGCCGGGCAATGGTGAAGCTCCAGTGTGTGGGCAGTCAGGTGCCCGAGGCTGTCCTAAGAGATTGCTGCCAGCAGCTGGCCGGGGACCTCAGCAGCATGTTGCGTAGTGTTTATCAGGAGCTCGGCGTGCGTGAGGGGAAGGAAGTGCTCCCAGGTTGCCGGAAGGAGGTGATGAAGCTCACGGCGGCGAGCGTGCCTGAGGTCTGCAAGGTGCCCATTCCCAACCCGTCGGGAGACAGAGCAGGTGTCTGCTACTGGGCCGCGTATCCGGACGTCTAGTCAAGCGAATCTGCATCTTAATTGGTGCGGTGCGTGCGCACAAGATAAATAAAATTAAGTAATGGTGCGGTGCGTGCGCACAAGATAAATAAAATTAAGTAATGGTGTATGTGCCTCT

> CLONE_31

TGAAGCAGCTTTTCCAAGAATGGAGCGGTCTTTGCACTCTGAAGACTATCTGTGGCAATATAATAGATAGCCTTTTGGGTCTCGGGCATATTCTCCACATACTGATCGAGACTTATCAAATCCGTTTCATTTTTAGAGGAATAAAACCGCAGCAAAGGAGCAAGGCGCTTCTGATTTCCTGAGTCCTCAATGCAACCAAGTTTCATAAATTTGCCAAAGCTCTCCCAAAATTTCTTGTAGTCCTCCTTGTTATCCTTGTCAGCAATATCCTGAATCATATCAAAAGTCTTCCTAACAAGTCTCTTGCGCATGATCCTGACAATACGACTTTCTTGAAGAATCTCACGAGAAACATTCAGAGGGAGATCATTCGAGTCAA

> CLONE_32

TGCCTTTTATTTTCCAAGTTTGAACTAGTGCCAGTCTAGTCCACACATGATAATGCATGGCACTTTATTT

TTTACCGCTACATGGATTGGACCATTGACTAAACAATGCTGGTCCGTTTTTTGGATACTAAACCTTTTA

TAGCCGCCCATGCCGGTAGTGACGTCAATGGATGGTGATATGGTGGTGTAGCAGTATGGTGGAACATGCACGTCGCACATTGCCGGAATGGTCTGAATCACCCATGTTCCTATCACCGCAATCCAACCCTGATTCAGTTGTGTTTGTTGTTGTGGCTGGCTAGAACCCTGACCTTGTTGTTGTTGACCTTGT

> CLONE_33

CATCAAGTTTCATGATGTTAAAGGCAAGAACTATAGGTGTGCCATCCCAAAACTAAACAAAGAAATAGTTCCCGAGAAATGCAAGATTGTGGTCAAGCCTACGAAGATCGTTATCACCTTGTGGAAAGCTTCTTCCGGCAATTGGTTGGACCTACACTACAAGGAAGACAAGCTTAAGCCAAGCATGGACAAAGAGAAGGATCCAATGTCAGGAATCATGGATTTAATGAAGGGCATGTATGAGGAAGGTGATGAAGATATGAAGCGCACAATAGCCAAGGCGTGGTCTGACGCCAGATCTGGGAAGACGGCTGATCCAATGAGGGGACTGCCTTGAGCTGGTTGTAGTTTTGG

> CLONE_34

CTACAACAAGGCGCTCGAGCAGCTCGACGCCGCCGTCAGCAAGGAGGACGCGTCCGCCGTCGTCCACCTCCAGAGCGCCATCAAGTTCAACGGCGGCGGTCATGTTAACCATTCAATCTTCTGGAAGAACCTCAAGCCCATCAGCGAGGGTGGTGGTGAGGCACCTCATGGCAAACTTGGCTGGGCCATTGATGAGGATTTT

GGTTCTATTGAGAAACTTATAAAGAAGATGAATGCAGAGGGTGCTGCTTTACAAGGATCTGGATGGGTGTGGCTAGCTTTGGATAAAGAGGCCAAAAGGCTTTCAGTTGAAACTACTCCTAATCAGGACCCTCTTGTGACCAAAGGGTCAAACCTGCATCCTTTGTTGGGAATTGATGTCTGGGA

> CLONE_35

TGGAAAACTTCAAGATGGGACAGTATTCTTGAAGAAGGGGCATGATGAACAAGAGCCATTTGAA

TTTAAGACTGATGAAGAGGCGGTCATTGAGGGTCTTGATCGTGCTGTGCTAAACATGAAGAAGGGCGAGGTTGCTCTTGTCACTATTCCTCCTGAATATGCTTATGGCTCAACGGAGTCGAAGCAGGATGCTATAGTTCCACCTAACAGTACTGTGATTTACGAAGTGGAGCTTGTATCATTTGTAAAGGATAAGGAGTCATGGGACTTGAACAACTCAGAGAAGATTGAGGCCGCTGGAACCAAGAAAGAAGAGGGGAATGCCTTGTTCAAATCGGGCAAATATGCCAGAGCTTCTAAGCGCTATGAGAAGGCTGCAAAGTTCATTGACTATGACACTTCGTTCAGTGAGGATGAGAAAAAGCAGTCCAAGCAACTGAAGATCACCTGCAACTTAAACAATGCTGCCTGC

# > CLONE_36

ATCAACTTGAACCTAGGCATAAGGAATTTTTGTTGGCCGGCGACTATAGGAGTTCGCAACTTCCCTCTAGAAAAAACATATTCAGTGGTTTTGATGTTAGGTTGCTTGGTGAGGCCTTGGCTACAAGTGGAAAGATAGCGCAAAGTCTTCAAAATCAAAATGATGACATAATTCATTTGAATCATACCCTTAAGTTTCTGAAGCCTATTTTAACACAAGAGCAAGAGCAAGAATTCTACCCACACACTGAGGAACGGCAATCTCAGGCAAAGCACTCTCGGGAAGAGCAACCTCAAATGGGGCAGTCTCAGGGAAAGCAACCTCAAACGGGTTGTCAGGCAAAGCACACTCTGGGAGAGCAACCTCAAACGAGGCAGTCTCAGGTAAAGCACTCTCAGGGAGACCAGCCTGAAGAAGGTGTAGGGAGGGCGATCTCAAGAAGAACAATCGCAGGCAAGGCCATATCTGGGATGTCAACCTCATCCA

> CLONE_37

AGATTTTGGAATTTCCCAAAGCAGCCCCAATAACATTCCATAAGAACCTAGCTAGTGAGCAAGAAAAATAATAAGTGATCAATGCTCTCTTCATTGTCACAAAATTCACATTTCCTACTTTCTGTATCATATAGAATTCCTAAGTGATTAGTGCCTCGCCGGGGATTCAAATTGTCAGAGCAAACTAAGCTAGACTACCAAGCCCGAGATTACAGAAAATGTTCAGACGATACTATACTGAAGTTCGCAGTAAGCAAACTTAAAGTGTTTTGAGTTCTTAACCCCCCAAAATGAAGTGATTTAGGCTTCAGTACGATTGGGAGCAACTGAATAATTCATGGTGAAAGAAAGACACAAAGAACTTGGTTATTCTTTAAGTGCTAATAATGATAGAGGAGATTAAGAATAA

TTCAGGACAAACATATACTACCATGCATA

> CLONE_38

ATGTGATTTCTGCCCAGTGCTCTGAATGTCAAAGTGAAGAAATTCAACCAAGCGCGGGTAAACGGCGGGAGTAACTATGACTCTCTTAAGGTAGCCAAATGCCTCGTCATCTAATTAGTGACGCGCATGAATGGATTAACGAGATTCCCACTGTCCCTGTCTACTATCCAGCGAAACCACAGCCAAGGGAACGGGCTTGGCGGGATCAGCGGGGAAAGAAGACCCTGTTGAGCTTGACTCTAGTCCGACTTTGTGAAATGACTTGAGAGGTGTAGGATAAGTGGGAGCCCTCACGGGCGCAAGTGAAATACCACTACTTTTAACGTTATTTTACTTATTCCGTGGGTCGGAAGCGGGGCGTGTCCCCTCCTTTTGGCTCCAAGGCCCGGTCTTACCGGGCCGATCCGGGCGGAAGACATTGTCNAGGTGGGNGAGTTTNGGCTGGGGCNGGCACATCTGTTAAAAGATAACG

> CLONE_39

CGGCGAGGCCGGCGGACCCGGCAATGCCGCCGGCGAGAAGCAGGGCCCGACGCGCGCCTACGTC

CGCGACGCGCGCGCCATGGCGGCCACCCCGGCCGACGTGAAGGAGCTGCCGGGCGCGTACGCGTTCGTGGTGGACATGCCGGGGCTGGGGTCCGGCGACATCAAGGTGCAGGTGGAGGACGAGCGCGTGTTGGTGATCAGCGGCGAGCGGCGGAGGGAGGAGAAGGAGGACGCCAAGTACCTGCGGATGGAGCGCCGCATGGGCAAGATGATGCGCAAGTTCGTGCTCCCCGAGAACGCCGACATGGAGAAGATCTCCGCCGTGTGCCGCGACGGCGTGCTCACCGTCTCCTTGGAGAAGCT

> CLONE_40

CATCCATAATGTTGCTCATGCTATTATTATGCATCAACAACAACAACAACAACAAGAACAACAACAACAGTTGCAACAACAACAACAGCAGCAACTGCAACAACAACGACAACAACCGTCGAGCCAGGTCTCCTTCCAACAGCCTCAGCAGCAATATCCATCAAGCCAGGTCTCCTTCCAGCCATCTCAGCTAAACCCACAGGCTCAGGGCTCCGTCCAACCTCAACAACTGCCCCAGTTCGCGGAAATAAGGAACCTAGCGCTACAGACGCTACCTGCA

> CLONE_41

ACAGTCCATAGCAGCGGAATGATTAGGGTGCTGCTGCTGCTGCTGCTGCATGTGATATGCGGCGGCGCCTTCATCCACGACGGTGCCGGCAGCCCAGGTGATCTGCTGCATGGCCCTGTAGTTGTTGCTGTTGCTGCCTTCAGCGTCGAGCTTGTGCTTGAGTCGCCTGTTGATGTCTCCCAGCTGACGCTCCTTCCTCCGAAGTTCCTCCACCTGCTCCATCATAAGTTGTGTCTTTCGCTGTCTGGCCAGTGACAGAGAACATTCTAGCTGTTTCTCCAGCTGCTGCAGTTCTTTCACGCTGAGCGGTCCTAGGTCCTCCCCAAGCAAGTGTCTCTGAGTTCGCTGCAAAGCTTCGAATTTTGCCTTTA

> CLONE_42

CGCAGTAATAATGCCCCTAATATTTTCAAAGATGCAAGAATCATATATCATCAAAAATAGGCTAATAAGCTTGGCTAAAGCATACGACAGGCTATTGCAAAAAGTTCACCATCCATTTGGTGATTTGCGGTGACCTACCTTTCTGTAAGCTTAAACTGAAGATACAGACATGGCTTTATCTCTCAACAGTTTTCCACCATGACACTGCGTGCAGAATGAGCAGAGTTCCTGCAGTAACAACTCCAACTGTCACTTTGTCGCTGCAATTTGCATCAAAATTGCCTATCGCACAAGCACCAAACCAACCAAGCCTGATTCAGTTATCATCTCCTACTGTTTGCTGGCTCGGCCTTGTTGAGGTTTGCAAGCTCTTCGATCAGCTTTCTCTCATCGCTGCTTAGACGCTTTGGAATCTCAACC

> CLONE_43

TACCTGCGGAACCTTAAAATCCTCACGCTTGACAGAAATAGGATCACTGTCTTGCCTGAAGAATTGGGTTCTCTATCAAATCTTCAGCAGCTTACAGTTTCTCAGAATTCTTTGCTGTGCCTACCTAAAAGTGTTGGAGATTTGTGCAATATGCTGCTACTCAATGTATCTGATAACAAACTAAATGCCCTTCCCGAATCAATAGGAGGCTGCAAGTCCCTCGAAGAATTGCAGGCTAATGGAAATACAATCGAAGATGTGCCCTCATCAATTTGCAACCTTGCTTGCCTCAAATCTATATCATTGAATGGAAACAAAATTCGTCAGCTTCCCCCAAACTTGCTGAAGGATTGCAAGGCTCTTCAGAACTTGTCACTGCACAGCAACCCAATCTCGATGGA

> CLONE_44

TGCTGAGATGCTTGTCAACCTCGGCGTTCCCTGGGTCATTCTTGGACACTCTGAAAGGAGGAGCTTGATGGGAGAATCAAGTGAGTTTGTTGGAGAGAAGGTTGCATATGCACTCGCTCAGGGCTTGAAGGTCATTGCATGTGTTGGTGAGACTCTTGAGCAGCGAGAAGCTGGATCAACCATGGAAGTTGTCGCTGAACAGACAAAAGCAATTACTGGCAAGATCAAGGACTGGACTAACGTAAGTCCATGCCAATCTGAGGGACTGGCTCAAGACCAATGTGAGCCCTGAGGTTGCTGCATCCACACGGATCNNATCTATGGAGGATCTGTAACTGGCGCAAGCTGGCAAGGAGTTGGCCGGGCAGCCCGACGTCGATGGTTTCCCTCGTTTGGTGGAGCTTCTTTGAAGCCTGAGTT

> CLONE_45

GTTCCCCGGGCTCGTAGAGGATAGCATTGCTCGAGACCATGTCATCCACCACGTTACTCTTGGAGATCTCCTTGGAACGGAATGTCTGCGGCGCAGATAGGTTCATCCCATCGTTGTTCCCCAGATGGTTGTAGCTGACAATTGAGGTGGGCTTGATTCCAGCACCAACAAGGAAATCCACCAAGACAGATTTCATCTTGGTCTGGCCACTCTTGAAATCATCGCCACCAATCAGGCAGTTGTTCTTAATAGCAAGATCGATCAGCCCAGGCACAAAGGTGTTCTGAGGGCTCCCGTTGATGAATGGCACACCCTCCATGACGCAGGCGATGGCA

> CLONE_46

TCTCCACGCAATCAATCAGCACCTGCCGCATGACAGGGTCCGTCAGGATGTTCTGGATTTCAGGGTCCTGCATAGCCTTGCCCTGTCTCTCCTTCTAGTCCTCCGGGGTAAGTTCGCCTCTGTTCGCCTTGTTGATCTGCTGAATACATCTCTTTACACCATCAGGAAGCTCCTGGTTACTCGGATCATGCTTTAGGCCTTCCTGATGAGTTTCCATTGCTTTGTCGAATTCTTTCAGGAGAGAGTGGATTGCGACCTTCGTGGTTGACCCTTGGAGAATGTTGGTGTGGCTCGATGCACTTAGCGGCGTCCTTGTTAGCTTTTGGCGTGGCCACCTGCTTGGTGTTGCAGG

> CLONE_47

ACACTGCTATTGCGCAATATGCGGAAGTTCCCAGCCCAGCTGCGCAAGCTCCCACCGCGGATGGTTCTGGGGAATGGGTTGCGATAGCACCTAGTGCGAGTGGTTCTGAGAATTGCGAGGAAGAGCAGCCAAAGCTAGACTCTTGCAGCGATTATGTTATGGATCGGTGTGTGACGAAGGATATGCCGCTCTCTTGGGTCTTTCCTCAGACTTGGGGGAAGAGAAGCTGTGAGGAGGTCCAAAACCAGTGTTGTCAGCAATTGAGGCAAACGACGCCGCGTTGCCGCTGCAAGGCTATATGGACATCAATCCAAGGCGATCTAAGTGGCTTCAAGGGCCTTCAACAAGGTCTGGAAGCCAAAACGGTGCAGACGGCCAAGAGCCTTCCCTCCAAGTGCAACATTGATCCAAAATACTGCA

> CLONE_48

TCGCCAACAAAGAACTGAAGGTCCTTGAGCTTGCTAAGAAGATACTTTGTGGCGGACTCAACATTCTTCTTGAAAGCATCTAGGTCATCCCCTTCAAGCTTGGCAGAGAGGTTCTTGATGTAGCGCTTCATGTGAGAGATAAACTGCTTCTTGTCAAAAGCAGGTTGCTCCTGAAGACGGAAGGTGTCAACAATGTCAACCACCTTCACGGCCTGGTCATCAACACCCTCATCATCACCACCACCCTCAGCAGAGGGATTGGCTCCAATGTCCACATCAACTGCTCCTTGAACGACCCAATGGCCATCGACTTCCCAGAGCACGCCGTTCTCCAGCTCCCTGTAAGGGAA

> CLONE_49

GACCCACGGAATAAGTAAAATAACGTTAAAAGTAGTGGTATTTCACTTGCGCCCGTGAGGGCTCCCACTTATCCTACACCTCTCAAGTCATTTCACAAAGTCGGACTAGAGTCAAGCTCAACAGGGTCTTCTTTCCCCGCTGATTCCGCCAAGCCCGTTCCCTTGGCTGTGGTTTCGCTGGATAGTAGACAGGGACAGTGGGAATCTCGTTAATCCATTCATGCGCGTCACTAATTAGATGACGAGGCATTTGGCTACCTTAAGAGAGTCATAGTTACTCCCGCCGTTTACCCGCGCTTGGTTGAATTTCTTCACTTTGACATTCAGAGCACTGGGCAGAAATCACATTGCGT

> CLONE_50

TACATGCCTATCACCAAGGCGGCGCGGGAGATGAACGTCGGTCTCACCGTGCTCAAGAAGCGCTGCC

GTGAGCTCGGTGTCGCCCGTTGGCCTCACCGGAAGATGAAGAGCCTCAGGTCTCTCATCCTTAACATCCAGGTACGTAGGTGCACACATATCGGTACAACACCACATTTGAAATGTCGCACACCGCATGTTAGCTATTCCCTCCATTCCAATTTGAACTAGAATCACCACAAGAATTATGGAACAACGGGTAATAGAAAATTGCGCGCAGGACATGGGGAAGGGCGCCACGTCGCCGGCGGCGGTGCAAGGGGAGCTGGAGGCGCTTGAGAGGTATTGTGCCATAATGGAGGAGAA

> CLONE_51

CGCTCTATTTGTGGGGGGCCTAAAGCCCGCTCCCTTGCTTTTTTTCCCTCCCTTTCTTTCCCGAATTGGGGGGTTTTCTCCCGAGAATTAAATAGGGGGGGGCCTCTTGGGGGCAAATAATTGTTGTTTGTGGCGCCCTCCCCCAAAAAAAATTTTTGGGGGGGGGTGTGCTCCAGGGGGGCGCCCCCCCCAAGAAAGAGTTTTTCCCCTTCTGTGGGGGGGGACCCCCTTTTTATAAAAGGGGACTGTGTTCGAAAGAGGGAGAAAACCACACCCCTTTGGGGGGTTTTTTTTGTTTATTAAAGAGGTTTTCCAACTATCCCCCTAGTGT

> CLONE_52

TGTGCTGACGATGTGAGGGTGCACGAAGTCCGTGTTCTTCTTCGCCTTGCCGAACACGTTGGCTGCTTCTCCCCCGCCGCCGCCGCCTACGGAGAAGCAGCCAACGTGTTCGGCAAGGCGAAGAAGAACACGGACTTCGTGGCGTACAGCGGCGAGGGGTTCAAACTGATGATCCCGGCCAAGTGGAACCCCAGCAAGGAGCGTGAGTTCCCTGGGCAGGTGCTCCGCTACGAGGACAACTTCGACGCCACCAGCAACCTCTCCGTTATAATCAACCCGACCAC

>CLONE_53

ACGTGTGGGAGTCGCAGGCCGGCGGCTCCTTCACCGTCACCCTCGACACGGAGGGCGAGCGGCTCGGGCGCGGCACCAAGATCACGCTCTTCCTCAAGGACGACCAGCTCGAGTACCTGGAGGAGCGCCGCCTCAAGGACCTCGTCAAGAAGCACTCGGAGTTCATCAGCTACCCCATCTACCTCTGGACCGAGAAGACCACCGAGAAGGAGATCAGCGACGACGAAGACGAAGACGCTTCCGAGGAGAAGAAGGAAGGCGAGGTCGAGGAGGTCGACGATGACAAGGACAAGGACGAGAGCAAGAAGAAGACGAAGAAGGTGAAGGAGGTGAGCCACGAGTGGGCGCAGATCAA

>CLONE_54

CAAGAAGACCATCACCGACTACGGTTCCCCTGAGGAGTTCCTCTCCCAGGTCGGCTTCCTCCTCGGCCAGCAGTCCTACGGTGGCAAGACCGACTCCGAGGGTGGGTTCGAGTCAGACGCTGTGGCGACGGCGAACGTGCTGGAGAGCTCGGCGCCGGTGGTGGACGGTAAGCAGTACTACAGCATAACGGTGCTCACGAGGACCGCGGACGGAGACGAGGGAGGGAAGCACCAGCTCATCACCGCCACCGTCGCCGACGGCAAGCTCTACGTCTGCAAGGCGCAAGCCGGAGACAAGAGGTGGTTCAAGGGCGCCAAGAAGTTCGTCGAGAACGCCGCAGGATCCTTCAGCGGCGCATAA

>CLONE_55

ATGGCCGCCCATGCAGCTCTCGCCGCCACCCGCATCCCCACCAGCGCGCGTCTGCACAGCAAGGCCGCCTCCAGGCAGAGGGTCGACTTCGCCGACTTCTCCGGGCTGAGGCCGGGGTCGTGCTCCGTCAGCGCCGCCGCCAGGGAGGCGTCCTTCTCCGATGTCCTCGGCGCGCAGCTCGTCGCCAGGGCTTCCGGCGAGAACGCCGTGAGGGCGCCGGCGGAGGCGAAGCTGAAGGTGGCCATCAACGGGTTCGGCCGCATCGGGCGCAACTTCCTCCGGTGCTGGCACGGCCGCGAGAACTCGCCGCTGGAGGTCATCGTCATCAACGACAGCGGAGGCGTCAGGAACGCGTCTCACCTGCTCAAGTACGACTCGATGCTGGGCAC

>CLONE_56

CTACGCCAAGTTCTACGAGGCATTCTCCAAGAACCTCAAGCTGGGCGTGCACGAGGACTCCCAGAACCGAGCCAAGCTCGCCGACCTGCTCCGGTATCACTCGACCAAGAGCGGCGACGAGCTCACCAGCCTCAAGGACTACGTCACCCGGATGAAGGAGGGCCAGAAGGACATCTACTACATCACCGGCGAGAGCCGCAAGGCCGTGGAGAACTCGCCCTTCCTGGAGCGCCTCAAGAAGCGCGGCTACGAGGTGCTCTTCATGGTGGACGCCATCGACGAGTACGCCGTGGGGCAGCTCAAGGAGTACGACGGCAAGAAGCTCGTCTCCGCCACCAAGGAGGGCCTCAAGCTCGACGAGGAGACCGAGGAGGAGAAGAAGCGCAAGGAAGAAAAAAAGGCGGCGTTCGAGGGCCTCTGCAAGATCATCAAGG

>CLONE_57

AGGGAATCGTTCCCGGTATCAAGGTTGACAAGGGTCTTGTGCCACTTGTTGGTTCCAACGATGAGTCATGGTGCCAAGGTCTAGATGGCCTTGCCTCCCGTGAAGCAGCATACTACCAGCAAGGCGCCCGCTTCGCCAAGTGGCGCACTGTTGTCAGCATTCCTAACGGCCCATCTGAGCTTGCTGTCAAGGAAGCTGCCTGGGGTCTTGCCCGTTACGCGGCCATCTCACAGGACAATGGGCTGGTGCCGATTGTGGAGCCTGAGATCATGCTCGACGGTGAGCACGGCATCGAGAGGACCTTCGAGGTCGCACAGAAGGTGTGGGCGGAGACCTTCTACTATATGGCCCAGAACAACGTCATGTTTGAGGGCATCCTCTTGAAGCCAAGC

>CLONE_58

CTGACCGCGTCGTCGACTCGCCGTGCTGCCTGGTGACCGGCGAGTATGGCTGGACTGCCAACATGGAGAGGATCATGAAGGCCCAGGCCCTGAGGGACACGAGCATGGGCGGCTACATGTCGAGCAAGAAGACGATGGAGATCAACCCGGAGAACGCCATCATGGAGGAGCTGCGCAAGCGCGCCGACGCGGACAAGAACGACAAGTCTGTCAAGGACCTGGTGATGCTGCTGTTCGAGACCTCCCTGCTCACCTCTGGCTTCAGCCTCGACGACCCCAACACCTTCGGCACCAGGATCCACCGCATGCTCAAGCTGGGCCTGAGCATCGACGAGGACGAGGAGGCTGCTGAGGCTGACACGGACATGCCGCCGCTGGAGGAGGACGCCGGCGAGAGCAAGATGGAGGAGGTCGACTAG

>CLONE_59

ATGAACCCCACCAACACCGTTTTTGGTAAGCCCCGCCTGCCCCGGTCTCTTATCTTATACAGTAGAATCCATGCTCGTGCAGTGAGAGTACGTGCTCTGCCTTTTGCTCTGTTTTACGCTAATGAGCAGTCACTTACCACTCTGCTCTGCCATGCAGATGCCAAGCGGCTCATCGGGCGGCGCTTCTCGGACGCATCCGTGCAGTCGGACATGAAGATGTGGCCGTTCAAGGTGGTCCCCGGCGCCGGCGACAAACCGATGATCGTGGTCAGCTACAAGGGGGAGGAGAAGACCTTCTCCGCCGAGGAGATATCCTCCATGGTGCTCACCAAGATGAGGGAGATCGCCGAGGCCTTCCTCAGCACGACCATCAACAACGCCGTCGTCACCGTCCCGGCGTACTTCAACGACTCCCAGCGCCAGGCCACCAAGGACGCCGGCGTGATCGCGGGTCTCAACGT

>CLONE_60

GAACATCGTGCCGACGAGCACGGGCGCCGCCAAGGCCGTCTCCCTGGTGCTGCCGCAGCTCAAGGGCAAGCTCAACGGCATCGCGCTCCGCGTGCCCACGCCCAACGTCTCCGTGGTGGACCTCGTCATCAACACCGTCAAGACCGGCATCACCGCCGACGACGTGAACGCGGCGTTCCGCAAGGCCGCCGACGGGCCGCTCAAGGGCATCCTCGACGTCTGCGACGAGCCGCTCGTGTCCGTCGACTTCCGCTGCTCCGACGTCTCCACCAGCATCGACGCCTCCCTCACCATGGTCATGGGCGACGACATGGTCAAGGTCGTCGCCTGGTACGACAACGAGTGGGGCTACAGCCAGCGCGTGGTTGATCTGGCGCACCTGGTGGCGGCCAAGTGGCCGGGCGCCGGGACCGGCGGCAGCGGCGACCCGCTGGAGGACTACTGCAAGACCGACCCCAACGCCGTGGAGTGCAAGGTGTTCGACGAGTGA

>CLONE_61

CGGCTTCCTCCTCGGCCAGCAGTCCTACGGTGGCAAGACCGACTCCGAGGGTGGGTTCGAGTCAGACGCTGTGGCGACGGCGAACGTGCTGGAGAGCTCGGCGCCGGTGGTGGACGGTAAGCAGTACTACAGCATAACGGTGCTCACGAGGACCGCGGACGGAGACGAGGGAGGGAAGCACCAGCTCATCACCGCCACCGTCGGCGACGGCAAGCTCTACGTCTGCAAGGCGCAAGCCGGAGACAAGAGGTGGTTCAAGGGCGCCAAGAAGTTCGTCGAGAACGCCGCAGGATCCTTCAGCGGCGCATAA

>CLONE_62

GACATCCTTGTCGAGCAGGGAATCGTTCCCGGTATCAAGGTTGACAAGGGTCTTGTGCCACTTGTTGGTTCCAACGATGAGTCATGGTGCCAAGGTCTAGATGGCCTTGCCTCCCGTGAAGCAGCATACTACCAGCAAGGCGCCCGCTTCGCCAAGTGGCGCACTGTTGTCAGCATTCCTAACGGCCCATCTGAGCTTGCTGTCAAGGAAGCTGCCTGGGGTCTTGCCCGTTACGCGGCCATCTCACAGGACAATGGGCTGGTGCCGATTGTGGAGCCTGAGATCATGCTCGACGGTGAGCACGGCATCGAGAGGACCTTCGAGGTCGCACAGAAGGTGTGGGCGGAGACCTTCTACTATATGGCCCAGAACAACGTCATGTTTGAGGGCATCCTCTTGAAGCCAAGCATGGTGACCCCTGGTGCTGAGTGCA

>CLONE_63

CATGCGCATAATCAACGAGCCCACCGCGGCGGCCATCGCTTACGGCCTCGACAAGAAGGCCACCAGCACCGGGGAGAAGAACGTGCTCATCTTCGACCTCGGCGGCGGCACCTTCGATGTGTCCATCCTCACCATCGAGGAAGGCATATTCGAGGTCAAGTCCACCGCCGGCGACACCCACCTGGGAGGCGAGGACTTCGACAACCGGATGGTGAACCACTTCGTGCAGGAATTCAAGAGGAAGAACAAGAAGGACATCAGCGGCAACCCGAGGGCGCTCCGGCGGCTCAGGACAGCGTGCGAGAGGGCGAAGAGGACACTCTCCTCCACCGCCCAGACCACCATCGAGATCGATTCCCTCTTCGAGGGCATCGACTTCTACACTACCATCACCCGCGCCCGGTTCGAGGAGCTCAACATGGACCTCTTCCGCAAGTGCATGGAGCCCGTTGAGAAGTGCCTC

>CLONE_64

ATGGCGTCTGCTGCTCTCCTCAAATCGTCTTTCCTTCCCAAGAAGGCCGAATGGGGTGCCACGCGCCAGGCCGCCGCCGCCAAGCCGGTGACCGTCTCCATGGTTGTCCGTGCCAGCGCGTACGCCGATGAGCTTGTCAAAACCGCGAAAACCATCGCATCGCCAGGCAGGGGTATCCTTGCCATGGATGAGTCGAATGCCACCTGTGGCAAGAGACTCGCCTCGATTGGCCTTGAGAACACCGAGGCTAACCGCCAGGCTTACCGGACCCTTCTTGTCACTCCACCAGGATTGGGAAACTACATCTCTGGTGCTATCCTCTTTGAGGAGACCCTCTACCAGTCGACTGTTGATGGCAAGAAGATTGTTGACATCCTTGTCGAGC

>CLONE_65

TCCACCAGCGTCGGCGAGAAGAACGTGCTCATCTTTGATCTCGGCGGCGGTACCTTCGATGTCTCCCTCCTCACCATTGAGGAGGGTATCTTCGAGGTCAAGGCCACTGCCGGAGACACCCACCTGGGAGGCGAGGACTTCGACAACCGGATGGTCAACCACTTCGTGCAGGAATTCAAGAGGAAGAACAAGAAGGACATCAGCGGCAACCCGAGGGCGCTCCGGCGGCTCAGGACAGCGTGCGAGAGGGCGAAGAGGACACTCTCCTCCACCGCCCAGACCACCATCGAGATCGATTCCCTCTTCGAGGGCATCGACTTCTACACTACCATCACCCGCGCCCGGTTCGAGGAGCTCAACATGGACCTCTTCCGCAAGTGCATGGAGCCCGTTGAGAAGTGCCTC

>CLONE_66

CGGGACGCCAAGATGGACAAGAGCACCGTGCACGACGTTGTCCTCGTCGGAGGATCCACACGTATCCCCCGTGTGCAGCAGCTCCTCCAGGACTTCTTTAACGGGAAGGAGCTCTGCAAGAGCATCAACCCTGACGAGGCCGTCGCGTACGGAGCTGCCGTTCAGGCTGCCATCCTCACTGGCGAGGGCAACGAGAAGGTGCAGGACTTGCTCCTGCTTGACGTCACACCGCTCTCGCAGGGCCTGGAGACGGCCGGAGGTGTCATGACCGTGCTGATCCCAAGGAACACGACAATCCCCACCAAGAAGGAGCAGGTCTTCTCCACCTACTCTGACAACCAGCCCGGCGTGCTCATCCAGGTGTATGAGGGCGAGAGGGCGAGGACCAAGGACAACAACCTGCTCGGCAAGTTCGAGCTCTCCGGGATCCCACCGGCGCCCAGGGGTGTCCCCCAGATCACC

>CLONE_67

AGGACAGGGCCACCCCTGAGGAAGTAGCCAGCTACACCCTCAAGCTTCTCCAGAGAAGGATCCCCCCTTCCGTCCCCGGCATCATGTTCTTGTCTGGCGGTCAGTCGGAGGTGGAGGCGACGCTGAACCTGAACGCGATGAACCAGGCGCCGAACCCATGGCACGTGTCCTTCTCCTACGCGCGGGCGCTGCAGAACACCTGCCTCAAGACGTGGGGCGGGCGGCCGGAGAACGTGGCCGCGGCGCAGGAGGCGCTGCTGCTGCGCGCCAAGGCCAACTCCCTGGCGCAGCTCGGCAAGTACACCAGCGACGGCGAGGCCGCGGAAGCCAGCGAGAACATGTTCGTCAAGAACTACAGCTACTGA

>CLONE_68

CCTCCACCTCCACCTTGACCTCCTCCTTTTTCACACCTGGAAGATCATCGTTCTTGTCCTCCTTCTCCTTTGTGCGCTCGCCGCTGACGACGAGCACGTTGCCGTCATCCACCTCCGCCAAGACCGTCTCGCTGTGCGTCAAGCGGCTCGTCTACACCAACGACGCCGGGGAGGTCGTCAAGGGCGTCTGCTCCAATTTCCTGTGCGACCTGAAGCCCGGTGCCGATGTCAACATAACAGGGCCGGTAGGCAAAGAAATGCTCATGCCTAAAGACCCAAATGCTACTATCATCATGCTCGCGACAGGGACCGGCATCGCGCCGTTCCGGTCATTCTTGTGGAAGATGTTCTTCGAGAAGTACGAAGACTACAAGTTCAATGGCCTGGC

>CLONE_69

ATGAAGGAGGGCCAGAACGACATCTACTACATCACCGGTGAGAGCAAGAAGGCTGTGGAGAACTCTCCCTTCCTTGAGAAGCTGAAGAAGAAGGGCTATGAGGTGCTGTACATGGTTGACGCCATTGATGAGTACTCCATTGGCCAGCTCAAGGAGTTTGAGGGAAAGAAGCTGGTCTCTGCCACTAAGGAGGGCCTGAAGCTTGATGACAGCGAGGAAGAGAAGAAGAGGAAGGAGGAGCTCAAGGAGAAGTTTGAGGGGCTCTGCAAGGTGATCAAGGAGGTGCTGGGCGACAGGGTCGAGAAGGTGATTGTCT

>CLONE_70

GTACACGCAAGCAAGCCCAGCCCAAACTACCTTCCGGCTGCAAAACAAGGCCCCAGCCCGGCCTGAATGGCATGCCCGACCCAGCCGTCAGGCAAGGCTGCCCATGTGCACAGGTTTAATTCAGGGTATTGATCATTGTAGACCCACGATTGATAGAGGTTCAAGCTACCCCAACGAATCTTTCCAGGTACTTGAGAAATCGAAAAGAGCGATTAATTATCGGAAACGAGGCAATGCCGTTTTCCTCTTTATCCTGCTCATCCCAACTAAAAGCAAAACAAGGCGGAAGCGCCTGACGAACTTGCCGCTGCTGCGCTCAACGCGGTGCCACTTGTCGTTCTTGTCCTCCTTCTCCTTTGTGCGCTCGCTGCTGACGACGAGCACATTGCCGT

>CLONE_71

GTGTGCTTTGACATTGATGCCAACGGTATCCTGAATGTCTCGGCGGAGGACAAGACCACCGGGCAGAAGAACAAGATCACAATTACCAACGACAAGGGGCGGCTGAGCAAGGAGGACATCGAGAAGATGGTGCAGGAGGCGGAGAAGTACAAGGCTGAGGACGAGGAGCACAAGAAGAAGGTGGACTCCAAGAACGCCCTGGAGAACTACGCTTACAACATGCGTAACACCATCAAGGATGACAAGATCGCCTCCAAGCTCCCAGAGGCCGACAAGAAGAAGATCGAGGATGCTATTGATGGTGCCATCACCTGGCTCGACAACAACCAGCTTGCCGAGGCTGAAGAGTTTGATGACAAGATGAAAGAGCTGGAGGGCA

>CLONE_72

CTGGCTCTTCTTGGGAGTCCCCACCAGCAGCTCTCTCCTCTACCCGGAGGAGTTTGGGAAGATGAAGGCGAAGGCGCCGGACAACTTCCGGGTGGACTACGCGATCAGCAGGGAGGAGACCAACGCGGCGGGGGAGAAGATGTACATCCAGACCAGGATGGCAGAGTACAAGGATGAGCTGTGGGAGCTGCTGAAGAAGGACAACACCTATGTGTACATGTGTGGACTGAAGGGCATGGAGAAGGGTATTGATGAGATCATGATCCCATTGGCTTCAAAAGAAGGGATCGACTGGATAGACTACAGGAAGCAACTGAAGAAGTCGGAGCAATGGAACGTCGAAGTCTACTGA

>CLONE_73

ATGAACCCGATCAACACCGTCTTCGATGCGAAGCGGCTCATCGGCCGGCGTTTCTCGGACTCGTCGGTCCAGAGCGACGCCAAGCTGTGGCCGTTCAAGGTGATCCCCGGTCCCGCTGACAAGCCGATGATCGGGGTACAGTACAGAGGCGAGGACAAGCAGTTCTCGGCCGAGGAGATCTCCTCCATGGTGCTCAACAAGATGAAGGAGACGGCCGAGGCCTACCTCGGCACAACCATCAAGAACGCAGTCGTCACCGTCCCCGCCTACTTCAACGACTCCCAGCGCCAGGCCACCAAGGATGCAGGCGTCATCTCTGGCCTCAACGTCATGCGGATCATCAACGAGCCTACCGCCGCCGCCATCGCCTACGGTCTTGACAAGAAG

>CLONE_74

GCAACGCGCTGGAGAACTACGCGTACAACATGCGCAACACGGTGCGGGACGAGAAGATCGCGTCCAAGCTCCCCGCCGAAGACAAGAAGAAGATGGAGATCGAGGACTCGATCGAGGACGCCATCAAGTGGCTCGACGGCAACCAGCTGGCCGAGGCCGACGAGTTCGAGGACAAGATGAAGGAGCTGGAGAGCATCTGCAACCCCATCATCTCCAAGATGTACCAGGGCGCCGGCCCGGGCGGCGCGGCCGGCATGGACGAGGACATGCCCGGCGGCGGCGCGGGCACCGGCGGTGGGAGCGGTGCCGGGCCCAAGATCGAAGAAGTGGACTGA

>CLONE_75

CGGGACGCCAAGATGGACAAGAGCACCGTGCACGATGTTGTCCTCGTCGGAGGATCCACACGTATCCCCCGTGTGCAGCAGCTCCTCCAGGACTTCTTTAACGGGAAGGAGCTCTGCAAGAGCATCAACCCTGACGAGGCCGTCGCGTACGGAGCTGCCGTTCAGGCTGCCATCCTCACTGGCGAGGGCAACGAGAAGGTGCAGGACTTGCTCCTGCTTGACGTCACACCGCTCTCGCAGGGCCTGGAGACGGCCGGAGGTGTCATGACCGTGCTGATCCCAAGGAACACGACAATCCCCACCAAGAAGGAGCAGGTCTTCTCCACCTACTCTGACAACCAGCCCGGCGTGCTCATCCAGGTGTATGAGGGCGAGAGGGCGAGGACCAAG

>CLONE_76

CTTCAAGGCGGACGTGAAGATCGTGGACAACGAGACCATCAGCGTCGACGGCAAGAACATCCAGGTCGTCTCCAACAGGGACCCCCTCAAGCTGCCATGGGCCGAGCTCGGCATCGACATCGTCATCGAGGGTACTGGAGTGTTCGTGGACGGCCCCGGCGCCGGGAAGCATCTCCAGGCCGGCGCCAAGAAGGTCATCATCACCGCTCCGGCCAAGGGCGCCGACATCCCCACCTACGTCGTCGGCGTCAACGAGGGCGACTACGACCATGACGTCGCCAACATCATCAGCAACGCTTCTTGCACCACCAACTGCCTCGCGCCATTCGCCAAGGTCCTGGACGAGGAGTTCGGAATCGTGAAGGGGACCATGACCACGACGCACTCGTACACGGGCGACCAGAGGCTGCTGGACGCGTCCCACCGCGACCTGCGGAGGGCGAGGGCAGCGGCGCT

>CLONE_77

AACGACAACTCCACCTACTCGGACAATCAGCCGGGCGTGCTGATCCAGGTGTTCGAGGGCGAGCGCGCGCGGACCAAGGACAACAACCTGCTGGGCAAGTTCGAGCTGTCCGGCATCCCCCCGGCGCCCCGCGGCGTGCCCCAGATCACCGTCTGCTTCGACATCGACGCCAACGGCATCCTCAACGTGTCGGCCGAGGACAAGACCGCCGGCGTGAAGAACAAGATCACCATCACCAACGACAAGGGGCGGCTGAGCACGGAGGACATCGAGCGCATGGTGCAGGAGGCGGAGAAGTACAAGTCGGAGGACGAGCAGGTGCGGCACAAGGTGGAGGCCC

>CLONE_78

TGACAAGATCGCCTCCAAGCTCCCAGAGGCCGACAAGAAGAAGATCGAGGATGCTATTGATGGTGCCATCACCTGGCTCGACAACAACCAGCTTGCCGAGGCTGAAGAGTTTGATGACAAGATGAAAGAGCTGGAGGGCATTTGCAACCCCATCATCGCCAAGATGTACCAGGGTGCCGGCGCTGAAATGCCTGGTGGCATGGATGAGGATGCTCCGGCCAGTGCCGCAGGCGGCAGCAGCGGCCCAGGGCCCAAGATTGAGGAGGTCGACTAA

>CLONE_79

CATCAGTGATGTATGGCTACTTCCTCAAGAGAGTTGATAAGAGGTTTCAGCTCGAGAAGTCGATGAAGAGCCTCCCTTGGGGATCAGAAGACGACGCGCTCAATCAAGTTATGACGACCGACTCACAGCCCTCAGATCAGACCTACAGTTCCCATCCAGAGGTGGAGTCGTGGACTTCCCCCGACCTCAGCGCAGGAGGTCTCGGCCAGTCTGTCAAGCCTTCCCGTCTTCGATCGTACGTCATGTCGTTTGATTCAGACACGTTACAAACTTACGCGACAATCCGGTCGAAGGTGGCATTTGGCATCATC

>CLONE_80

ATGAATCTGGAGGTGGCCGACGCCATGTTGGTGCTTGCCATCGGGACGGCAGCAAGGGCTCAAAAGTATGTAAGTCACGAAGTCCGTGTTCTTCTTCGCCTTGCCGAACACGTTGGCTGCTTCTCCCCCGCCGCCGCCGCCTACGGAGAAGCAGCCAACGTGTTCGGCAAGGCGAAGAAGAACACGGACTTCGTGGCGTACAGCGGCGAGGGGTTCAAACTGATGATCCCGGCCAAGTGGAACCCCAGCAAGGAGCGTGAGTTCCCTGGGCAGGTGCTCCGCTACGAGGACAACTTCGACGCCACCAGCAACCTCTCCGTTATAATCAACCCGACCACCAAGAAGACCATCACCGACTACGGTTCCCCTGAGGAGTTCCTCTCCCAGGT

>CLONE_81

ATGGAGGCAGCAGCAGCAGCAGCCGTGGCGCTCAGGTCGCCCGCCGCCGCCGGAGCCGCGCCCTCGCGCCGGGCCGCCGCCGCCAGCTCGCTCCCCTTCGAGCGGAGGCGCAGCTTCGCCCTCGGCTCCATCAAGGGCCTCGGGCGGCGGCAGCTCACTTCCAGAAGGCTGCGCAGCGTGGTCAGGGCATCCTCCTCGTCCCCGTCGGAGTCTCTGCCATCTTCTTCGCCGATCGCGCCGCTGCAAATGGAGTCGCCGATAGGGCAGTTCCTCTCACAGATTTTGGTCACGCACCCGCACCTGCTCCCGGCCGCCGCCGAGCAGCAGCTCGAGCAGCTCCA

>CLONE_82

AAGGACATCAGCGGCAACCCTCGCGCGCTGCGCCGGCTGCGCACGGCCTGTGAGCGCGCCAAGCGCACCCTGTCGTCCACCGCCCAGACCACCATCGAGATCGACTCGCTGTACGAGGGCGTCGACTTCTACTCGACCATCACCAGGGCCCGGTTCGAGGAGCTCAACATGGACCTCTTCCGCAAGTGCATGGAGCCCGTGGAGAAGTGCCTGCGCGACGCCAAGATGGACAAGAGCACCGTGCACGACGTCGTGCTCGTCGGCGGCTCCACCCGCATCCCCAAGGTGCAGCAGCTGCTGTGCCTCCGTGACGCCAAGATGGACAAGAGCACGGTGCACGACGTGGTGCTGGTCGGTGGCTCCACCCGTATCCCCAAGGTGCAGCAGTTG

>CLONE_83

GACCGTCCAAGATTCTTCAGAAAAGAAGGACGCGCAGGCACCGCCGGCGGCCGGCGATATCGTGCTGTACAGGCGAATTGCCGAGGTTAAGGAGAAGGAAAGGAAAAGGACCATGGAGGAGATACTGTACGCGCTGGTTGTTCAGAAGTTTGTTGAAGCTGGCGTCTCTTTGGTCCCAGCTCTCTCTCACTCCATTGATGCTTCTTCTGGTAGAGTTGATCAGTGGGCAGAGCATGTGGAAGGGAAGCTTGAGCGTTTGCACTCACACGAGGCATATGAAATGATCGAGAACCACTTGAATCTCATATTGGGGCAGCGGCAAGCTGATGGCACTGTCGCAGCCATAAGTAAGCTCCGAGTTGGCCAGGTCTATGCCG

>CLONE_84

CTCCAGGACTTCTTCAACGGCAAGGAGCTGTGCAAGAGCATCAACCCCGACGAGGCAGTGGCGTATGGCGCCTCCGTCCAGGCTGCCATCCTGAGCGGCGAGGGCAACGAGAAGGTGCAGGACCTGCTGCTGCTCGACGTGACGCCGCTGTCCCTGGGCCTGGAGACCGCCGGCGGCGTCATGACGACGCTCATCCCGAGGAACACCCCCATCCCCACCAAGAAGGAGCAGGTCTTCTCCACCTACTCGGACAACCAGCCTGGCGTGCTGATCCAGGTGTACGAGGGCGAGAGGACGAGGACCAAGGACAACAACCTGCTGGGCAAGTTCGAGCTGTCCGGCATCCCGTCGGCGCCCAGGGGCGTACCCCAGATCACGGTGACCTTCGACATCGACGCGAACGGCATCCTGAACGTGTCGGCGGAGGACAAGACGACCGGGCAGAAGAACAAGATCACCATCACC

>CLONE_85

ATGGTGGACGCCATCGACGAGTACGCCGTCGGGCAGCTCAAGGAGTACGACGGCAAGAAGCTCGTCTCCGCCACCAAGGAGGGGCTCAAGCTCGACGAAGAAACTGAAGAAGAAAAGAAGCGCAAGGAAGAGAAGAAGGCCGCCTTCGAGGGACTCTGCAAGACCATCAAGGACATCCTCGGCGACAAGGTGGAGAAAGTCGTCGTCTCGGACCGCATCGTGGACTCGCCGTGCTGCCTGGTGACCGGCGAGTACGGGTGGACGGCCAACATGGAGCGGATCATGAAGGCGCAGGCGCTGAGGGACAGCAGCATGGGCGCATACATGTCCTCCAAAAAGACCATGGAGATCAACCCGGAGAACGGCATCATGGAGGAGCTCCGCAAGCGCGCCGACGCCGACAAGAACGACAAGAGCGTCAAGGACCTCGTGA

>CLONE_86

GCTCATCAAGATGAAGGAGATCGGCGAGGCCTACCTCGGCACCACCATCAAGAACGCCGTCGTCACGGTGCCGGCCTACTTCAACGACTCGCAGCGGCAGGCCACCAAGGACGCCGGGGTCATTGCCGGCCTCAATGTGATGCGCATCATCAACGAGCCCACTGCCGCCGCCATAGCCTACGGCCTGGACAAGAAGGCCAGCAGCTCCGGCGAGAAGAACGTGCTCATCTTCGACCTTGGTGGCGGCACCTTCGATGTGTCGCTGCTCACCATCGAGGAGGGCATCTTCGAGGTGAAGGCCACCGCCGGCGACACCCACCTCGGCGGCGAGGACTTCGACAACCGCATGGTGAACCACTTCGTCCAGGAGTTCAAGCGCAAGCACAAA

>CLONE_87

ATGGCGGACGTGCAGATGGGCGGCGGCGCGGCGGAGACGGAGACGTTCGCGTTCCAGGCGGAGATCAACCAGCTGCTCTCGCTCATCATCAACACCTTCTACTCCAACAAGGAGATCTTCCTCCGCGAGCTCATCTCCAACTCCTCAGATGCGCTTGACAAGATCCGGTTCGAGAGCCTCACCGACAAGAGCAAGCTGGACGCGCAGCCGGAGCTCTTCATCCGCCTCGTCCCCGACAAGGCCACCAAGACGCTCTCCATCATCGACAGCGGCGTCGGCATGACAAAGTCAGACCTTGTGAACAACCTGGGCACCATCGCGCGCTCCGGCACCAAGGAGTTCATGGAGGCGCTTCAGGCGGGCGCGGACGTGAGCATGATCGGCCAGTTCGGGGTCGGCTTCTACTCGGCCTACCTCGTCGCCGACAAGGTGGTGGTCACCACCAAGCACAACGACGACGAGCAGTACGTGTGGGAGTCGCAGGCCGGCGGCTCCTTCACCGTCACCCTCGACACGGAGGGCGAGCGGC

>CLONE_88

GACAACAACCTGCTCGGCAAGTTCGAGCTCTCCGGGATCCCACCGGCGCCCAGGGGTGTCCCCCAGATCACCGTGTGCTTTGACATTGATGCCAACGGTATCCTGAATGTCTCGGCGGAGGACAAGACCACCGGGCAGAAGAACAAGATCACAATTACCAACGACAAGGGGCGGCTGAGCAAGGAGGACATCGAGAAGATGGTGCAGGAGGCGGAGAAGTACAAGGCTGAGGACGAGGAGCACAAGAAGAAGGTGGACTCCAAGAACGCCCTGGAGAACTACGCTTACAACATGCGTAACACCATCAAGGA

>CLONE_89

TCGGGCGCGGCACCAAGATCACGCTCTTCCTCAAGGACGACCAGCTCGAGTACCTGGAGGAGCGCCGCCTCAAGGACCTCGTCAAGAAGCACTCGGAGTTCATCAGCTACCCCATCTACCTCTGGACCGAGAAGACCACCGAGAAGGAGATCAGCGACGACGAAGACGAAGACGCTTCCGAGGAGAAGAAGGAAGGCGAGGTCGAGGAGGTCGACGATGACAAGGACAAGGACGAGAGCAAGAAGAAGACGAAGAAGGTGAAGGAGGTGAGCCACGAGTGGGCGCAGATCAACAAGCAGAAGCCCATCTGGCTGCGCAAGCCGGAGGAGATCAGCAAGGAGGAGTACGCGTCCTTCTACAAGAGCATCACCAACGACTGGGAGGATCACCTG

>CLONE_90

ATGGCCAAGGGCGAGGGGCCGGCGATCGGCATCGACCTCGGGACGACCTACTCCTGCGTCGGCGTGTGGCAGCATGACCGCGTCGAGATCATCGCCAACGACCAGGGCAACCGCACCACGCCCTCCTACGTCGCCTTCACCGACACCGAGCGCCTCATCGGCGACGCCGCCAAGAACCAGGTCGCCATGAACCCCACCAACACCGTCTTCGATGCCAAGCGGTTGATCGGAAGGCGGTTCACCGACCCGTCCGTGCAGAGCGACATGAAGCTGTGGCCGTTCAAGGTCATCCCAGGCCCTGCTGACAAGCCGATGATCGTCGTCCAGTACAAGGGCGAGGAGAAGCAGTTCGCCGCCGAGGAGATCTCCTCGATGGT

>CLONE_91

GCCGTGAAGCACTTCTCGGTGGAGGGGCAGCTGGAGTTCAAGGCGGTGCTCTTCGTGCCCCGCCGCGCGCCCTTCGACCTCTTCGACACCCGCAAGAAGATGAACAACATCAAGCTCTACGTGCGCCGCGTCTTCATCATGGACAACTGCGAGGAGCTCATCCCGGAGTGGCTGGGCTTCGTCAAGGGCGTCGTCGACTCGGACGACCTCCCCCTCAACATCTCCCGCGAGACGCTGCAGCAGAACAAGATCCTCAAGGTCATCCGCAAGAACCTCGTCAAGAAGTGCATCGAGCTCTTCTTCGAGATCGCCGAGAAC

>CLONE_92

GCCATACACCACCACAGCCCCACTCTCAGAGAGCACGGACGCCGGACACGCACGAGTCTTCTTGAAGCAATGGCGACCACCGCGATGGCGCTCTCTTCCTCGTCCTTCGCCGGGAAGGCGGTGAAGGACCTGCCGTCGTCGGCGCTCTTCGGGGAGGCGCGCGTCACCATGCGCAAGACCGCGGCCAAGGCCAAGCCGGTGTCGTCCGGCAGCCCGTGGTACGGCTCCGACCGCGTGCTCTACCTCGGCCCGCTCTCCGGCGACCCCCCGAGCTACCTCACCGGTGAGTTCCCCGGCGACTACGGGTGGGACACCGCGGG

>CLONE_93

CCCTCGTCGAGCGCGCGTTTTCCAGGAGCTTCAGGTGCCGGCGGCGCCATGGCTTCGGAGACCGAGACCTTCGCCTTCCAGGCCGAGATCAACCAGCTGCTCTCGCTCATCATCAACACCTTCTACCTCCAACAAGGAGATCTTCCTCCAATCACACTAAATTGCGTGTTTGGGTGCTCTGATGTGCAGGCGTTGGATAAGATCAGGTTTGAGAGCCTGACTGACAAGAGCAAGCTGGATGCTCAGCCAGAGCTGTTCATCCACATAATCCCTGACAAGGCCACGAACACACTCACGCTTATTGACAGTGGTATTGGTATGACCAAGTCAGACCTTGTGAACAACCTTGGTACCATTGCAAGGTCTG

>CLONE_94

CCCTCGTCGAGCGCGCGTTTTCCAGGAGCTTCAGGTGCCGGCGGCGCCATGGCTTCGGAGACCGAGACCTTCGCCTTCCAGGCCGAGATCAACCAGCTGCTCTCGCTCATCATCAACACCTTCTACCTCCAACAAGGAGATCTTCCTCCAATCACACTAAATTGCGTGTTTGGGTGCTCTGATGTGCAGGCGTTGGATAAGATCAGGTTTGAGAGCCTGACTGACAAGAGCAAGCTGGATGCTCAGCCAGAGCTGTTCATCCACATAATCCCTGACAAGGCCACGAACACACTCACGCTTATTGACAGTGGTATTGGTATGACCAAGTCAGACCTTGTGAACAACCTTGGTACCATTGCAAGGTCTGGCACCAAGGATTTCATGGAGGCAC

>CLONE_95

TGGCCTGGCTCTTCTTGGGAGTCCCCACCAGCAGCTCTCTCCTCTACCCGGAGGAGTTTGGGAAGATGAAGGCGAAGGCGCCGGACAACTTCCGGGTGGACTACGCGATCAGCAGGGAGGAGACCAACGCGGCGGGGGAGAAGATGTACATCCAGACCAGGATGGCAGAGTACAAGGATGAGCTGTGGGAGCTGCTGAAGAAGGACAACACCTATGTGTACATGTGTGGACTGAAGGGCATGGAGAAGGGTATTGATGAGATCATGATCCCATTGGCTTCAAAAGAAGGGATCGACTGGATAGACTACAGGAAGCAACTGAAGAAGTCGGAGCAATGGAACGTCGAAGTCTACTGA

>CLONE_96

TGGCTGCTGGTGCTGATGTGTCCATGATTGGTCAGTTTGGTGTCGGTTTCTACTCTGCTTACCTTGTTGCTGAGAGAGTCGTTGTGACCAGCAAGCACAACGACGACGAGCAGTATGTGTGGGAGTCCCAGGCTGGTGGTTCCTTCACTGTGACACGTGATACTACTGGAGAGCCCCTTGGAAGGGGTACTAAGATCACCCTCTACCTCAAGGATGATCAGTTGGAGTACCTTGAGGAGCGTCGCCTTAAGGATCTGGTGAAGAAGCACTCTGAGTTCATCAGCTATCCCATCTCTCTCTGGACGGAGAAGACCACTGAGAAGGAAATTTCTGACGATGAAGATGAGGATGAGAAGAAGGATACTGAGGAGGGCAAGGTTGAGG

>CLONE_97

ATGGCCTTCACCTCGGGCTTCTTGACGTGGGCCTTGGGCACGGTGACGGTGAGCACCCCGTTCTCCAGCCTGGCCTTCACCTCCTCCACCTTGGCGTCCTCCGGCAGGCGGAAGCGCCTGACGAACTTGCCGCTGCTGCGCTCAACGCGGTGCCACTTGTCGTTCTTGTCCTCCTTCTCCTTTGTGCGCTCGCTGCTGACGACGAGCACATTGCCGTCCTCCACCTCCACCTTGACCTCCTCCTTTTTCACACCTGGAAGATCATCGTTCTTGTCCTCCTTCTCCTTTGTGCGCTCGCCGCTGACGACGAGCACGTTGCCGTCATCCACCTCCGCCAAGACCGTCTCGCTGTGCGTCAAGCGGCTCGTCTACACCAACGACGCCGGGG

>CLONE_98

AGAACAAGAAGGCCGTGGAGAACTCTCCCTTCCTTGAGAAGCTGAAGAAGAAGGGCTATGAGGTGCTGTACATGGTTGACGCCATTGATGAGTACTCCATTGGCCAGCTCAAGGAGTTTGAGGGAAAGAAGCTGGTCTCCGCCACCAAGGAGGGCCTGAAGCTTGATGACAGCGAGGAAGAGAAGAAGAGGAAGGAGGAGCTCAAGGAGAAGTTCGAGGGGCTCTGCAAGGTGATCAAGGAGGTGCTGGGCGACAGGGTTGAGAAGGTGATCGTCTCTGACCGCGTCGTCGACTCACCGTGCTGCCTCGTCACCGGCGAGTACGGCTGGACCGCCAACATGGAGAGGATCATGAAGGCCCAGGCCCTGAGGGACACGAGCATGGGCGGCTACATGTCCAGCAAGAAGACGATGGAGATCAACCCGG

>CLONE_99

TCAGAGCATCCTTGCCATCTGGGCTTGCCAGGTCGTGCTCATGGGCGCTGTCGAGGGGTACCGCGTTGCTGGTGGCCCGCTCGGAGAGATCGTCGACCCACTCTACCCCGGTGGCAGCTTCGACCCCCTTGGCCTAGCCGACGACCCGGAGGCGTTTGCTGAGCTCAAGGTGAAGGAAATCAAGAACGGCCGCCTTGCAATGTTCTCCATGTTTGGATACTTTGTGCAGGCCATCGTCACTGGTAAGGGTCCGCTGGAGAACCTCGCTGACCACCTCGCCGACCCTGTCAACAACAACGCGTGGGCCTTCGCCACCAACTTTGTGCCCGGCAAGTAA

>CLONE_100

TTGAGAAGCTGAAGAAGAAGGGCTATGAGGTCATCTACATGGTTGACGCCATTGATGAGTATGCCATTGGTCAGCTCAAGGAGTTTGAGGGCAAGAAGCTTGTCTCTGCCACCAAGGAGGGTCTGAAGCTTAAGGCCGTGGAGAACTCTCCCTTCCTTGAGAAGCTGAAGAAGAAGGGCTATGAGGTGCTGTACATGGTTGACGCCATTGATGAGTACTCCATTGGCCAGCTCAAGGAGTTTGAGGGAAAGAAGCTGGTCTCCGCCACCAAGGAGGGCCTGAAGCTTGATGACAGCGAGGAAGAGAAGAAGAGGAAGGAGGAGCTCAAGGAGAAGTTCGAGGGGCTCTGCAAGGTGATCAAGGAGGTGCTGGGCGACAGGGTCGAGAAGGTGATTGTCTCTGACCGCGTCGTCGACTCGCCGTGCT

>CLONE_101

ACAAGAGCCTGACAAACGACTGGGAGGAGCACCTTGCCGTCAAGCACTTCTCGGTGGAGGGTCAGCTTGAGTTCAAGGCCGTCCTCTTCGTTCCCAAGAGGGCCCCCTTTGACCTCTTTGACACCAGGAAGAAGCTCAACAACATCAAGCTCTATGTGCGCCGTGTCTTCATCATGGACAACTGTGAGGAGCTGATCCCAGAGTGGCTGAGCTTTGTCAAGGGCATTGTTGACTCTGAGGATCTTCCCCTGAACATCTCTCGTGAGACACTCCAGCAGAACAAG

>CLONE_102

GCCTGGTCACCGGCGAGTATGGCTGGACCGCCAACATGGAGAGGATCATGAAGGCCCAGGCCCTGAGGGACACGAGCATGGGCGGCTACATGTCCAGCAAGAAGACGATGGAGATCAACCCGGAGAACGCCATCATGGAGGAGCTGCGCAAGCGCGCCGACGCGGACAAGAACGACAAGTCTGTCAAGGACCTGGTGATGCTGCTGTTCGAGACCTCCCTGCTCACCTCTGGATTCAGCCTCGACGACCCCAACACCTTCGGCACCAGGATCCACCGCATGCTCAAGCTGGGCCTGAGCATCGACGAGGACGAGGAGGCTGCTGAGGCTGACACGGACATGCCGCCGCTGGAGGAGGACGCCGGCGAGAGCAAGATGGAGGAGGTCGACTAG

>CLONE_103

ATCCTCAAGGTCATCCGGAAGAACCTTGTGAAGAAGTGCATCGAGCTCTTCTTTGAGATTGCCGAGAACAAGGAGGACTACAACAAGTTCTATGAGGCTTTCTCCAAGAACCTCAAGCTCGGCGTCCACGAGGACTCCACCAACAGGACCAAGCTTGCTGAGCTCCTGAGGTACCACTCCACCAAGAGCGGCGATGAGCTTACCAGCCTCAAGGACTATGTGACGAGGATGAAGGAGGGCCAGAGTGACATCTACTACATCACCGGTGAGAGCAAGAAGGCTGTGGAGAACTCTCCCTTCCTTGAGAAGCTGAAGAAGAAGGGCTATGAGGTCATCTACATGGTTGACGCCATTGATGAGTATGCCATTGGTC

>CLONE_104

GTCGTCACCGTCCCGGCGTACTTCAACGACTCCCAGCGCCAGGCCACCAAGGACGCCGGCGTGATCGCGGGTCTCAACGTCATGCGCATAATCAACGAGCCCACCGCGGCGGCCATCGCTTACGGCCTCGACAAGAAGGCCACCAGCACCGGGGAGAAGAACGTGCTCATCTTCGACCTCGGCGGCGGCACCTTCGATGTGTCCATCCTCACCATCGAGGAAGGCATATTCGAGGTCAAGTCCACCGCCGGCGACACCCACCTGGGAGGCGAGGACTTCGACAACCGGATGGTGAACCACTTCGTGCAGGAATTCAAGAGGAAGAACAAGAAGGACATCAGCGGCAACCCGAGGGCGCTCCGGCGGCTCAGGACAGCGTG

>CLONE_105

GGATCATGAAGGCCCAGGCCTTGAGGGACTCGAGCATGGCTGGCTACATGTCGAGCAAGAAGACCATGGAGATCAACCCTGAGAACGCCATCATGGACGAGCTCCGCAAGCGCGCCGACGCCGACAAGAACGACAAGTCAGTCAAGGACCTCGTGATGCTGCTCTTCGAGACCTCCCTGCTCACCTCCGGCTTCAGCCTGGAGGACCCCAACACCTTCGGCACCAGGATCCACCGCATGCTCAAGCTTGGCCTGAGCATTGACGAGGACGACGAGGCGCCAGAGAACGACACCGACATGCCACCCCTGGAGGACGACGCCGGCGAGAGCAAGATGGAGGAGGTCGACTAA

>CLONE_106

CCCTCGTCGAGCGCGCGTTTTCCAGGAGCTTCAGGTGCCGGCGGCGCCATGGCTTCGGAGACCGAGACCTTCGCCTTCCAGGCCGAGATCAACCAGCTGCTCTCGCTCATCATCAACACCTTCTACCTCCAACAAGGAGATCTTCCTCCGCGAGCTCATCTCCAACGCCTCCGATGGTTTGAGAGCCTGACTGACAAGAGCAAGCTGGATGCTCAGCCAGAGCTGTTCATCCACATAATCCCTGACAAGGCCACGAACACACTCACGCTTATTGACAGTGGTATTGGTATGACCAAGTCAGACCTTGTGAACAACCTTGGTACCATTGCAAGGTCTGGCACCAAGGATTTCATGG

>CLONE_107

CGAGAGGGCGAAGAGGACACTCTCCTCCACCGCCCAGACCACCATCGAGATCGATTCCCTCTTCGAGGGCATCGACTTCTACACTACCATCACCCGCGCCCGGTTCGAGGAGCTCAACATGGACCTCTTCCGCAAGTGCATGGAGCCCGTTGAGAAGTGCCTCCGGGACGCCAAGATGGACAAGAGCACCGTGCACGACGTTGTCCTCGTCGGAGGATCCACACGTATCCCCCGTGTGCAGCAGCTCCTCCAGGACTTCTTTAACGGGAAGGAGCTCTGCAAGAGCATCAACCCTGACGAGGCCGTCGCGTACGGAGCTGCCGTTCAGGCTGCCATCCTCACTGGCGAGGGCAACGAGAAGGTGCAGGACTTGCTCCTGCTTGACGTCACACCGCTC

>CLONE_108

ATGAACCCCACCAACACCGTTTTTGGTAAGCCCCGCCTGCCCCGGTCTCTTATCTTATACAGTAGAATCCATGCTCGTGCAGTGAGAGTACGTGCTCTGCCTTTTGCTCTGTTTTACGCTAATGAGCAGTCACTTACCACTCTGCTCTGCCATGCAGATGCCAAGCGGCTCATCGGGCGGCGCTTCTCGGACGCATCCGTGCAGTCGGACATGAAGATGTGGCCGTTCAAGGTGGTCCCCGGCGCCGGCGACAAACCGATGATCGTGGTCAGCTACAAGGGGGAGGAGAAGACCTTCTCCGCCGAGGAGATATCCTCCATGGTGCTCACCAAGATGAGGGAGATCGCCGAGGCCTTCCTCAGCACGACCATCAACAACGCC

>CLONE_109

AGAATTCCCAAGATCAGAAGCCTTGTCCTGGGATTGTGCAAGAAGGGAGACTCTTATGGAAGCATCGACGATCTTGAAGCCGCTGTTTCAGGTGCTGCACTGGAAGGAGCCATTGCTTCAGGAGTCACCGACCCTTCAGGGAGCCTGGATCTGCTGACGATTCAGGCGACCCCGATGAACCTCGGGATCCGTGCCGACGGGGACGGCTTTGCAGCCATCATTCCAAGGAACACCGCTGTCCCGGCTAGAAGGGACATGCTGTTTACAACAACGCAGGACAACCAGGCCGAGGCGCTGATCGCCGTCTACGAAGGCGAGGGGGAGCAGGCGGAAGAGAACCATCTCCTGGGGTACTTCAAGATCACCGGCATCCCG

>CLONE_110

CAGCTTCTGAAAAGAAAGATGAAGGAAATGCGTGGTTTAAGATGGGCAAGTACGCCAAGGCTTCCAAGAGATATGAGAAGGCTGCAAAGTACATCGAGTATGACAGCTCCTTCAGCGAGGACGAGAAGAAGCAATCCAAGGCAGTGAAGATCAGCATCAAGCTAAACAACGCAGCGTGCAAGCTGAAGCTAAAGGATTACAAAGAAGCAGAGAAGCTCTGCACCAAGGTTTTGGAACTGGAGAGCACAAACGTCAAGGCTCTGTACCGGAGGGCGCAGGCGTACACCGAGCTCGTGGATCTGGAACTGGCAGAACTAGATATCAAGAAGGCTCTGGAGATCGACCCGGACAACAGGGAGAAGCACCGT

>CLONE_111

ATGGCTGAGCAATTCTACACAGTGGCATCTGACAGTGAAACCACCGGGGACGACAAAGTGCAACAATCATTCCCTGATGTTGCTATCGGCATTGATATTGGCACTTCAAGATGCAGTGTTGCTATTTGGAATGGTCATCAAGTGGAGCTACTGAAAAACACTCGGAGCCAGAAAGGGATGAGGTCATATGTCATGTTCAAAGATGACACCCTTTCAGCGGGTGTTACTGGAGGAGCAACCCAGGAGCACCCACACGAGGAAAGAGATATCTTGTCAGGAAGTGCAATATTTAACATGAAGCGTTTAATCGGGAGAATGGACACAGA

>CLONE_112

CATCGCCGCCGGCAACTACCCGGAGTGGACCTTCTACATCCAGACCATCGACCCGGACTATGAGGAGCGGTTCGACTTCGACCCGCTGGACGTGACCAAGACGTGGCCCGAGGACGTGGTGCCGCTGCAGCCGGTGGGGCGGCTGGTGCTCAACCGCAACATCGACAACTTCTTCGCGGAGAACGAGCAGCTGGCCTTCTGCCCCGGGATCATCGTCCCCGGGGTGTACTACTCGGACGACAAGCTGCTGCAGACGAGGATCTTCTCCTACTCGGACACGCAGCGCCACCGCCTGGGGCCCAACTACCTGCTGCTGCCGGCCAATGCGCCCAAGTGCTCCCACCACAACAA

>CLONE_113

ATGGCCGCCGTGGAGGATGAGACGCTGAGGGCGCTCGAAGAGGAGGACGACGCCATGATGAACGAGCCGGACGGCGAGCCCATGGACGACGAGGAGGAGGCCGACTCCCCGGCGACGATGAAGGTCGGCGAGGAGAAGGAGATCGGGAAGCAGGGGCTGAAGAAGAAGCTCGTCAAGGAGGGCGAGGGCTGGGAACGGCCCGAGACCGGCGACGAGGTCGAAGTGCACTACACGGGCACTCTCCTCGACGGGACCAAGTTCGATTCGAGCCGGGACCGCGGGACTCCCTTCAAGTTCAAGCTCGAGCAGGGCCAGGTGATCAAGGGATGGGACCAGGGCATCAAGACCATGAAGAAGGGCGAGAACGCCGTCCTCACCATCC

>CLONE_114

ACAATGGCTCCACACTCATGGCTGGGGTCATCGCCAACAGGGAGGGGATCTCATGGGCGACCAAGGACAAGGTGCAGCAGGCCAACTACTTTGGGTCCCTCACCCAGGCCTCCACCATCAGGGTCGGGAGCTACAACGGAGAGGAGATCTATGCGCCCTTCAAGAGCCTCCTGCCCATGGTAAACCCAGATGACCTTGTGTTTGGGGGCTGGGACATTAGCAGCATGAACCTGGCTGATGCTATGACCAGGGCCAAGGTGCTGGACATTGACCTGCAGAAGCAGCTCAGGCCCTACATGGAGTCCATCGTGCCGCTCCCCGGCATCTATGACCCGGACTTCATCGCTGCCAACCAGGGCTCCCGGGCTAACAATG

> CLONE_115

CTCCAACGGCGCTAGCCTCGGCAGCGTCAGCAACGGTGGAAGGATCAGGTGCATGCAGGTGTGGCCCATCGAGGGCATCAAGAAAGTTCGAGCCCTGTCTTACCTGCCACCGCTCAGCACGGAGGCCCTCCTCAAGCAGGGGGACTACCTGATCCGCTCCAAGTGGGTGCCTTGCCTTGAGTTCAGCAAGGTCGGGTTCATCTTCCGTGAGCACAATGCGTCTACCCTGTCTTACTTGCCACCGCTCAGCACGGAGGCCCTCCTCGAGCAGGTCGACTACCTGATCCGCTCTAAGGTCGACTACCTGATCCGCTCCAAGTGGGTGCCCTGCCTTGAGTTCAGCAAGGTTGGCTTCGTCTTCCGTGAGCACAACA

>CLONE_116

GATCCCGAGGTCCCATCCTGCTGGAGGACTACCACCTGGTGGAGAAGATCGCCGACTTCGACCGCGAGCGCATCCCGGAGCGGGTGGTGCACGCCCGGGGCGCCAGCGCCAAGGGCTTCTTCGAGGTGACCCACGACGTGTCCCACCTGACCTGCGCCGACTTCCTCCGCGCCCCCGGCGTGCAGACCCCCGTCATCGTGCGCTTCTCCACCGTGATCCACGAGCGCGGCTCCCCCGAGACGCTCCGCGACCCGCGCGGCTTCGCCATCAAGTTCTACACCCGGGAGGGCAACTGGGACCTGGTCGGCAACAACTTCCCCGTCTTCTTCATCCGCGACGGCATGAAGTTCCCGGACATGGTGCACGCGCTCAAGCCCA

>CLONE_117

CCTCATCGCCGAGAAGAACTGCGCGCCCCTTATGCTCCGCCTCGCGTGGCACTCGGCCGGGACCTTCGACGTGGCCACCAAGACCGGCGGGCCCTTCGGCACCATGAAGTGCCCCGCGGAGCTCGCGCACGGCGCCAACGCCGGCCTCGACATCGCCGTCAGGCTGCTCGAGCCCATCAAGGAGCAGTTCCCCATCCTCTCCTACGCCGACTTCTACCAGCTCGCTGGAGTCGTCGCCGTCGAGGTCACCGGCGGGCCTGAGGTTCCCTTCCACCCGGGGAGACAGGACAAGCCCGAGCCTCCTCCAGAAGGCCGTCTTCCTGATGCTACCCAAGGCTCTGACCACCTCAGGCAGGTGTTTTCCACTCAGATGGGTTTGAGTGACCAGGACATTGT

> CLONE_118

CGGAGGAAGCCCCCGCGCCGGTGGAGGCCGAAGTCGAGGCGCCTGCCCCCGTCGAGGTTGAGGAGACCAAGGAGGAGGCGCCGGCAGCAGTTGAGGCAGAGGAGACGAAGGAGCCCGAGCCAGCAGCTGCGGCCGAACCGGAGGCCGAGGCGCCCGCCGTGGTCGAGACAGAGACCAAGGAGGCCGTGGCTGAGGCAGAGCCGGCGGCGGCCGAGGAGGTGAAGGAAGAGGCGGCGCCTGCTGCCGAGGCCGAGCCGGCCGCTCCCGCTGCTGCTGAGGAAGCACCGGTGGAGGCAGCCGCGGCCGCGGTGGCCAGCGTCAGCGTCTGGAGCTTTGTTGTGGTGTGTGTCACAGTGTCAGTTCCACCTATGATCGATCGGTATCCTATCCGGTTTGTATTCAGACCCGCGCAGTTCCGGTCG

>CLONE_119

CGTGCGAGCGTGCCAAGCGCACGCTGTCGTCGACGGCCCAAACCACCATCGAGATCGACTCGCTGTACGAGGGTGTGGACTTCTACACCACCATCACACGGGCTCGCTTTGAGGAGCTCAACATGGACCTCTTCCGCAAGTGCATGGAGCCGGTGGAGAAGTGCCTCCGCGACGCCAAGATGGACAAGAGCACGGTGCACGACGTGGTGCTGGTCGGTGGCTCCACCCGTATCCCCAAGGTGCAGCAGTTGCTCCAGGACTTCTTCAACGGCAAGGAGCTGTGCAAGAGCATCAACCCCGACGAGGCAGTGGCGTATGGCGCCTCCGTCCAGGCTGCCATCCTGAGCGGAGAGGGCAACGAGAAGGTGCAGGACCTGCTGCTGCTCGACGTGACGCC

>CLONE_120

CGCGCGAGCGCGACGTCGGCGTGCCCAGCTTCTTCTCAGATGTGTTCCGTGATCCGTTCAGCGCGCCGCAGAGCCTGGGCCGGCTGCTGAGCATGCTGGACGACGTGTCGGCGGCGTCCCCCGACGGAGCCGCGCGGGCCGCGCCGATGCGGCGCGGGTGGAACGCGAGGGAGGACGCGGACGCGCTGCGGCTGAGGGTGGACATGCCGGGGCTGGGCAAGGAGCACGTGAAGGTGTGGGCGGAGCAGAACAGCCTGGTGATCAAGGGCGAGGGCGAGAAGGAGTCGGAGCAGGAGGGCGCCGACGCGCCGAGGTACAGCGGCCGGCTCGAGCTCGCCGGGGACGTGTACCGGATGGACCAGATCAAGGCCGAGATGAAGAACGGCGTGC

> CLONE_121

CTAAGAACCAGGTCGCCATGAACCCCACCAACACCGTCTTCGATGCGAAGCGGCTGATTGGCAGGAGGTTCTCTGACCCCTCCGTACAGAGTGACATGAAGCTGTGGCCCTTCAAGGTCATCCCTGGCCCGGCTGACAAGCCTATGATCGTCGTCAACTACAAGGGCGAGGAGAAGCAGTTTGCTGCGGAGGAGATCTCCTCCATGGTGCTCATCAAGATGAGGGAGATAGCTGAGGCCTTCCTCGGCAACTCCATCAAGAACGCCGTGGTCACCGTCCCGGCCTACTTCAACGACTCCCAGCGCCAGGCCACCAAGGACGCTGGTGCCATCGCTGGGCTGAACGTGCTGCGCATAATCAACGAGCCCACTGCTGCTGCCATTGCCTATGGCCTTGACAAGAAGGCGACCAGCACCGGCGAGAAGA

> CLONE_122

CCTTCGAGGTTGACGCCAACGGTATCCTGAACGTGAAGGCTGAGGACAAGGGCACGGGCAAGTCTGAGAAGATCACCATCACCAACGAGAAGGGCCGTCTGAGCCAGGAGGAGATCGACCGGATGGTCAAGGAGGCGGAGGAGTTCGCCGAGGAGGACAAGAAGGTGAAGGAGCGCATCGACGCCCGGAACCAGCTGGAGACGTATGTCTACAACATGAAGAACACCGTCGGCGACAAGGACAAGCTGGCGGACAAGCTGGAGAGCGAGGAGAAGGAGAAGGTGGAGGAGGCCCTCAAGGAGGCGCTGGAGTGGCTGGACGAGAACCAGACGGCCGAGAAGGAGGACTACGAGGAGAAGCTCAAGGAGGTGGAGGCCGTGTGCAACCCCATCGTGTCGGCCGTGTACCAGAGGTCCGGCGGAGCTCCGGGCGGCGAGGGCGCCGACGGCGGCGTC

> CLONE_123

CGACGGCGACGCGGGCTGCTGCCTGGCGCCGACGCCGCTGGACCTGTCGGCGGCCGCGGCGGTGCCGCCGTTCCTGGCCAAGACGTTCGACATGGTGGAGGACCCGGCGACGGACGCGGTGGTGTCGTGGGGCGCGGCGCGGAACAGCTTCGTGGTGTGGGATCCCCACGCCTTCGCCGCCCGCCTCCTCCCGCTGCACTTCAAGCACGCCAACTTCTCCAGCTTCCTCCGGCAGCTCAACACCTACGGATTCCGTAAGAAGAAGCGGCGGCTGCTCGACGCCGTCCCGTCGCCGCCACCGGCGGAGGACGGGTTCACCTTCGAGGAGCTGGCACTGGCGGCCGGCGTCGTCGAGGAG

> CLONE_124

GGAGATCAACAAGGAAGAGTACGCAGCTTTTTACAAGAGCTTGACCAATGACTGGGAGGAGCATTTGGCTGTCAAGCACTTCTCTGTTGAGGGTCAGCTTGAGTTCAAGGCTGTCCTGTTTGTGCCCAAGAGGGCCCCCTTCGACCTCTTCGACAACAAGAAGAAGGCCAACAACATCAAGCTGTACGTGCGCCGTGTCTTCATCATGGACAACTGTGAGGAGTTGATCCCTGAGTACCTGAGCTTTGTCAAGGGTATTGTTGATTCAGAGGACCTTCCCCTGAACATCTCCCGTGAGACTCTCCAGCAGAACAAGATCCTCAAGGTCATCAGGAAGAACCTTGTCAAGAAGTGCATTGAGCTCTTCTTTGAGATTGCTGAGA

> CLONE_125

CCATTCAATGCCTCCTTCCCTCGCCCTTCCCTGTCCAAGTCCATCGGCAACGGCGTGCAGTTCCTCAACAGGCACCTGTCATCGAAGCTCTTCCATGACAAGGAGAGCATGTACCCCTTGCTCAACTTCCTCCGCGCACACAACTACAAGGGCATGACTATGATGCTGAACGACAGAATCCGCAGCCTCAGTGCTCTCCAGGGAGCTCTGAGGAAGGCCGAGGAGCATTTGTCTGGTCTCCCAGCAGACACTCCATACTCGGACTTCCACCACAGGTTCCAAGAACTTGGCCTGGAGAAGGGTTGGGGTGACTGCGCCAAGCGTGCGCAGGAGACCCTTCACCTGCTCCTCGACCTTCTCGAGGCTCCCGACCCGTCCACGCTCGAGA

> CLONE_126

GCTCTACAAGGGGACCACCAAGAAGATGAAGATCTCCAGGGAGATCGCCGATGCCAGCGGGAAGACGATCCCGGTGGAGGAGATCTTGACGATCGACGTGAAGCCGGGGTGGAAGAAGGGCACGAAGATCACCTTCCCGGAGAAGGGCAACGAGCAGCCCCACACGATTGCGGCGGACCTGGTCTTCATCATCGACGAGAAGCCGCACCCGGTGTACACCCGGGACGGCAACGACCTGGTGGCGACGCAGAAGATACCGCTGGCAGAGGCCCTGACGGGGTACACGGTGCACCTGACGACGCTGGACGGGCGCAGCCTGACGGTGCCCATCAGCTCGGTGATCCACCCGGGGTATGAGGAGGTGGTGCGCGGCGAGGGCATG

> CLONE_127

TCATGACCTCGAACCTTGGCGCGGAGCACCTGCTCGCCGGAATGGTGGGCAACTCGATGAAGGTTGCTCGCGATCTCGTCATGCAGGAGGTGAGGAGGCATTTCCGGCCGGAGCTGCTGAACCGTCTGGACGAGATCGTCATCTTCGACCCTCTGTCGCACGAGCAGCTGCGGAAGGTTGCCCGGCTTCAGATGAAAGATGTGGCAGTACGTCTTGCGGAGAGGGGCGTTGCCTTGGCCGTCACAGACGCTGCCCTGGACGTCATCCTCTCACTCTCTTATGATCCGGTGTACGGCGCCAGGCCAATCAGGAGATGGATCGAGAAGAGGATAGTGACGGAGCTTTCCAAGATGTTGATCCGCGAGGAGATCGACGAGAACTCCACGGTGTACA

> CLONE_128

GGAACTTGTTAACCTTGTGGTTGTCTGTGGTGACCATGGCAATCCATCAAAGGACAAGGAGGAGCAGGCTGAGTTCAAGAAGATGTTTGACCTTATTGAGCAGTACAACCTGAATGGCCATGTCCGCTGGATCTCAGCCCAGATGAACCGTGTCCGTAATGCTGAGCTCTACCGTTACATCTGTGACACCAAGGGTGCCTTTGTGCAGCCTGCTTTCTATGAGGCTTTTGGGCTTACTGTCATCGAGGCCATGACCTGCGGTCTTCCTACCTTTGCAACTGCATATGGTGGTCCTGCTGAGATCATCGTGAATGGTGTGTCCGGCTACCACATTGATCCCTACCAGGGTGACAAGGCCTCCACTCTG

> CLONE_129

TACATCGCCACCGGCATCCGCGTCGGCTTCAGCTCCGTCTGCAGCGCCCACGCCGACACCCACCCGCCGCCCTCCAAGCCCAAGGCGCCGCCGACCGCCGCCGTCCCCAGGGCGGCGCCGGTCTCCGCCGCGGCGCAGGAGGTGCCGTCGTCGCCGTTCCTGCCGTCGTCCGGCTGGGCCGTCGAGGACCTCCTGCAGTTCTCCGACTACGAGTCCAGCGACAAGAAGGGTTCTCCGTCTCCTCTCGGGTTCAAGGAGCTGGAGTGGTTCGCCGACATCGACCTGTTCCACGACGACCAGGCGCCGGCGCCAAAGTGGGGCGGAACCGCGGCGGAGGTGCCCGAGCTCTTCGCCTCGCCGCAGCCGGCCAGCAACGCCGGCTTCTACAAGACAGCCGGAGCGCGCCAGA

>CLONE_130

TGGGGCAGATGAAGAAGCTGTGCAACAACATCGTGAGCCTCATGTCCAAGTTCGCCTCGAGCCAGCAGCCGGACGGGGGCCCCGGGTCGCTGTCGTCCGTGGTGAACTGCTCGGGCGAGTCGGCGCTGGCCCCTCCGCCGCCGCTCCCCGCGGGGATACTGGACCTGATGCCATCGTGCTCGGCGCTGGCCACGGCCGCCGGCCTCGCCGTCGACGGCGGGCCGGAGACGGACGCGAGGCTGTTCGGCGTCTCCATAGGGCTCAAGCGAGCGCGAGACGAAGAAGAAGACGGCGAGGGCGAGGAGCTTCCTAACAGCAACGACGACGGCGCGGACGTGAAGCCGGAGGAGGCAGCGGAGCGGCGGCCCGAGGGCGGCGGCAGCGAGGAGCGGCAGTCGTGGCCGATATACCGGCCCAAGCCCGTGTACCGGGCCTGCAACGGGCAGGACGGGGCCG

> CLONE_131

TCAACCCCAGGCACCCCATCATCAAGGAGCTCCGCGACAAGGTCGCCCAGGACAGCGATGGCGAGGGCCTGAAGCAGACGGCGAGGCTGGTGTACCAGACGGCGCTGATGGAGAGCGGGTTCAACCTGCCGGACCCCAAGGACTTCGCCTCGAGCATCTACCGGTCGGTGCAGAAGAGCCTGGACCTGAGCCCTGACGCCGCCGTGGAGGAGGAAGAGGAGGTGGAGGAGCCCGAGGTGGAAGAGAAGGAGTCGGCCAAGGCGGAGGAGCCCGAGCACGAGCAGTACGACAAGGACGAGCTG

>CLONE_132

TGATTGGTGACGGAGTACAAATTCTGGGTGGTTTCCTTGGAACAAGGGCTGAGCCAATTGAATTCCCCGTTCGAACCAATCAAATACCAAGAGAAATCCCTTCAAAGAATTACTCATGGCTGCTCATATTTGGTGCTGGAACTCTACCTTTCGGAACACTCTTCATTGAGCTCTTCTTCATTCTCTCAAGCATCTGGCTCGGAAGGTTCTATTATGTGTTTGGGTTCCTCCTTGTTGTGCTCCTTCTGCTGGTTGTGGTATGCGCTGAGGTATCTGTTGTTCTTACGTACATGCACCTCTGTGCTGAGGACTGGAGGTGGTGGTGGAAAGCTTTCTTTGCCTCTGGAGCAGTGGCATTTTATGTGTTCCTCTACTCTATCAACTACTTGGTGTTTGATCTCAGAAGCTT

> CLONE_133

GCCAGGTGCCGCCGCCGGGGGCCGGCGGCGGCGCGGGCGGGCCGGGGGGCGCCACCTTCTTCTCCACCGGCGCCGACGGGCCCACCGCGTTCCGTTTCAACCCGCGCAACGCCGAGGACATCTTCGCCGAGTTCTTCGGCTCCTCCAGCCCCTTCGGCGGGATGGGGGGCATGGGGGGAGGAGGCCACGGCATGCCGTCCGGCGGCATCCGGTTCTCGCCGTCCATGTTCGGCGGCGGCGACCACCATACCTTCACCCAGACCTTCGGCGGCGGGGGCGGCCAAGGATACCCCGGGATGTTCGGCAGCGGCGGCGGCGGCGCGCCGGTCAAGGCGGCGCCCATCGAGAGGAAGCTGCCCTGCTCCCTCGAGGA

> CLONE_134

CAGAAGCTGACATCACGCGGTACTGGTGCCATGAATGTCAGCAGGCCGTCGAGGAGGCCATGGTGGAGGAGCTCAAGTGTCCATTGTGTGATGGTGGGTTCGTCGAAGAGATGACCGTTGAGCAGGTTGAGGCATTGACAGAGCAAGGGGCAACTCAGTGGGACCCTCTGGACAACCCTTTTGAGCAGGCAGGATCGCCGGGGGACAGCGATGATGAAGATAATAGCGATATAGGCCGTGAGTTTGAGGGTTTCATCAGAAGGCATCGACGGGCATCGGCGCTGCGCCGTGTGCTTGACAGCATCCATGATGACCTTAGAGATGACAGGGAAAGGGACAACTCCGTTCTGATCAGTGCTTTCAACCAGGCCCTCGCTCTACAAGGTGCAGCGCTTGACCCTGACGAGGACCGAGGTGACAATGGTAACTCAAATAATGATGATGGTTTGCTAGACGAGTATGTCCTC

>CLONE_135

CCTCCTCGCCGTCGCCCTCCGCCGCCTCGCCGCCGTCCCCTTCCTCTTCGCCCGCGGCGTTCCCGCTCTTCGACAAGCTGAGGCCGGCCGCCGTCGCGTCGCCCCTGCTGCAGGCGGCGCCCGTCTACATGGCCGTCGACCGGCCGGCCGCCGGGCCGGCGGACCCGAAGGCGTCCAAGTCGTCCGTCAACCGCTGCCACAACTGCCGGAAGCGCGTGGGCCTGACGGGGTTCCGCTGCCGGTGCGGCGAGATGTTCTGCGGCGCGCACCGGTACTCGGACCGGCACGACTGCAGCTACGACTACAAGTCGGCGGCCAGGGACGCCATCGCCCGGGAGAACCCCGTCGTGCGCGCCGCCAAGATC

>CLONE_136

ACGACGCTGGCGACGCCTACGGCCGCCACCCTCCCGCCGCCTACGGCGCTCCTCCCCCGGCCTACGGTGCCCCGCCTCCCGCCTACGGGGGCGGCCGCGAGGATGAGTACGGAGGCCGCGCCCCGGCATACGGTGCCCCGGCCCCGGCCTACGGTGGAGGCCGCGAGGACGACTACGGGCGCCACTCGCCCGCGCCTGCGGGCTACGGCGGTGGTGACTACGGGCGCCACGCCCCTGCTCCGGCCTACGGAGGCGGCCGCGACGAAGGCTACGGCGCACCCGCCCACGGCAACGTGGTGCACGTCTCCCACGAGTCCGGCGACGAGAGGCCGCAGTACGGGGGCTACGGCCACGAGACGCGCCCGCACCACGGCGGCGGCGGGATGG

> CLONE_137

AGGTCCTGTTCGAAAATTTCCGTTACTCGAGGGATACAAAGCTTAGTATAGTGGTTGTCCTTGTAGGTGTGGGAGTGTGTACAGTTTCTGATGTTAGTGTAAATGCGCAAGGATTGGTAGCTGCCGTAATAGCAGTTTGCGGCACTGCATTACAACAGCATTATGTCAATTACCTTCAACGGAAGTACTCTCTCAACTCACTCAAACTCTTGGGTCACACTGCACCAGCTCAAGCAGCTTCACTGTTGATATTAGGCCCATTCGTGGACTTCTGGCTGACCAGGAATAGAATCGACACTTTTCACTACACCAGCACAGTGACGTTCTTCATTGTGCTGTCATGCGTTATTTCAGTTGGGACCAATCTCAGCCAATTCATATGCAT

>CLONE_138

CGCCCCCGGCGACCAGGCAGCCGACGTACAGGATCCTCTGCAAGGCCGGCGAGGACAGCTTCAGCCTCGCCGCCAGGGACGGCAAGGTCTGCCTCGTCCGCACCGATCGCGACGACGACACGCAGCACTGGATCAAGGACATGAAGTACAGCACAAGGGTCAAGGATGAGGAAGGCTACCCTGCCATGGCACTCGTCAACAAGGCCAGCGGAGAGGCTCTCAAGCACTCCCTCGGCCAATCTCACCCTGTTCGTCTGACCAGGTACAATCCAAACACCCTGGACGAATCGGTCCTCTGGACCGAGAGCAGGGACGTCGGAGAAGGCTACCGCTGCATCAGGATGGTGAACAACATCTACTTGAACTTTGATGCACTCCATGGCGACAAGGACCATGGCGGTGTGCGCGATGGAACCACCCTCATTCTGTGGGAGTGGACTGAG

> CLONE_139

GCAAGAGTTGAGGGCACTTGCACAGAAGGCCCGCATGGAAAGGTCTGGTGCTCCACTTCCATCCACGGGTATGCCTGTCGGAGGTGGGAGGGAGAGAGAGCGGGAGAGGGTTGATGATGGGGATGCAGATATGGATTTGGAGCAGCCGCGTGAGCAGCGCAGGGAGACTAGAGAAGAGAGGGAGGCGAGGATTGAGCGTGACAGGATCCGTGAGGAGCGGAGGCGTGAGAGGGAGAGGGAGAGGAGGCTGGAGGCAAAGGAGGCTGCTGGAACGCATAAAAAGAGTAAGCTCACTAGAGACAGGGACCGTGACGTCGGTGAGAAGGTGGCCCTGGGTATGGCACACACTGGTGCGAAGACCGGGGAGGTCATGTATGACCAGAGGCTCTTTAACCAGGACAAGGGA

>CLONE_140

AGGCAAATCTAGGACTGAGTATTGAAGATATAATTCAGGAATGCAAGTTATTTTACTTTGCAGGTATGGAGACAACATCTGTCTTGCTCACATGGACACTAATTGTGCTAAGCATGCACCCAGAGTGGCAAGAGCAGGCAAGAGAGGAAGTGTTGCATCACTTTGGAAGAACCACACCAGATTTTGAGAACTTGGGTCGCCTTAAGATTGTAACAATGATTTTCTATGAGGTTCTTAGGTTGTACCCGCCAATAACCTTCCTTACGAGAAGAACTTACAAGGCAATGGAGCTTGGCGGCATCAAATATCCGGCAGGCGTGAA

>CLONE_141

GCAGCAGGACGGCGGCGGCAGCGGCAGCGGCAAGCGCGGCATCTCCCGGAGCGAGGCGCCGTCGTTCGCGTCCGCGTCCTCCTCCTCCACCTCGTCGTCCTCCGAGGACGCGCTGCCGCGGGCGTCCACGCCGCGGTCGGTGCTCCCCGCGGAGATCTCGCGGCGGGAGCTGGAGGCCGTGCTCCGGCGGCTGGGCCACGAGGAGCCGTCGGACGACGAGCTCGACGCCGTGGCGGCCATCGCGGCCGCCGGGGAGGCCGGCCCCGAGGACGAGCTGATGGAGGCGTTCAACGTGTTCGACGCCGACGGCGACGGCCGCATCACCGCCGAGGAGCTCCGCGGCGTCATGGTCGCCATCCTCGGCGGCGAGGCCGACGGGTGCAG

>CLONE_142

TCAAATTGCTCAACTAGGGGAGGAACTTAAATCTACTCTTGTAATTGTAATCTCTAAGAGTGGTGGTACACCTGAAACCCGGAATGGTCTACTAGAAGTACAAAAAGCCTTCAGAGATGCTGGACTGGATTTTTCAAAACAGGGTGTTGCAATCACTCAAGAAAATTCTCTATTAGATAACACTGCCAGAATAGAGGGATGGTTAGATCGATTTCCTATGTTTGACTGGGTTGGTGGTAGAACATCAGAATTGTCAGCGGTGGGGTTACTTCCAGCTGCATTACAGGGTATCGATGTCAAGGAAATGCTAGTTGGTGCAGCACTAATGGATGAGGAGACCAGGAACACCGTGGTTAAGGAAAATCCAGCAGCATTACTTGCGTT

>CLONE_143

GAGCTTGAACCTGGAGTCACATGTGGTGACTACTTGTTTGGGATGTTGCAGGGTACACGCTCTGCTCTTTATTCTAATGACCGAGAGTCCATCTCCGTTACTGTGGAAGAAGTAACTCCCAGAGCTGTTGGAGCACTGGTTGCACTCTATGAACGTGCAGTTGGAATTTATGCTTCTTTAGTAAACATCAATGCATATCATCAGCCTGGTGTCGAGGCAGGGAAAAAAGCAGCAGGAGAGGTATTGGCCCTTCAGAAAAGGGTGCTGACTGTTCTCAATGAGGCCAGCTGCAAAGACCCTGCTGAACCATTGACCCTGGAGCAAATCGCAGATCGTTGCCATTGCCCTGAAGATATTGAGATGATATACAAAATAATTCAGCACATGGCAGCCAACGATAGAGCACT

>CLONE_144

CCTTATGTTGCCCATTCTGTTTATCCACCATGACCCTGATATTTGGGGAAAAGACGCAAGCGAATTCAATCCACAGAGGTTTGCTGATGGCATCTCAAATGCAGCGAAACATCCGGCGGCATTCTTTCCTTTTGGAGGGGGTCCTCGGATATGCATCGGCCAGAACTTTGCGTTGCTGGAAGCAAAGATGGCTCTGAGCATCATCCTCCAGCGCTTCTCGTTCGAGCTCTCGCCGTCCTACATTCACGCACCGTACACCGTGATAACCCTGCAGCCGCAGCACGGTGCGCAAATTAGGCTGAAGAAGATATGA

>CLONE_145

AACTAAAGAAGATGGCCCTTAGAGTAATTGCCGAGAATCTGTCTGAAGATGAAATTGCAGGATTAAGAGAGATGTTCAAAATGCTCGATACTGACAATAGTGGCCAAATCACATTGGAGGAACTAAAAACTGGCTTGCAGAGAGTTGGTGCCAACTTAAAAGAGTCAGAAATCGCAACTCTAATGGAAGCGGCGGATATTGATAACAGTGGGTCAATTGATTATGGGGAATTCCTTGCGGCAACATTGCATCTGAACAAGGTCGAGAGAGAAGATAATTTGTTTGCAGCATTCTCATACTTCGATAAAGATGGCAGTGGTTACATTACTCAAGATGAACTGCAAAAAGCATGTGAAGAGTTTGGTATAGGAGATGCACATCTTGACGATATTAT

>CLONE_146

TTACGAAGGACGACATAAGGGAACTCATATACAGAGAAATTCTGGAATATCATCCAAACATGCTGAGGGAATTCCTTGAGGGGGCAGAGCCAACTAATTTCATGTACCCAAGTGCAGTAGATCATTTCAAAAAGCAATTCACATTCCTTGAAGAGCATTATGCAAAGGGATCAACAGCAGCGCCGCCTGAGAGGCAACATAATTCATTACCAAGGCCGAGTGTTATCTATTCGGATAATCGGCCACAGGGTTCAGATAGCCGACCACAGGGTGCTGCCAACATTACGGATGATCTTTCCAGGTGTATAATCA

>CLONE_147

TGGTCAGTGGAAGTACCGCCTAAGCGGTACTACTGCTCCCATGAGCGGCCTGAACCTGTGGAGGAACTTGCCGCTGCTGCGCTCCACCCTGTGCCAGGTGTCAGTCTTCTCCTTCTGCTCCTTGTTCCGCTCGCCGCTGATCTGGATGATGTTGTCGTTCTCCACCTCCACCTTCACCCCCTCCTTCTTCAGCCCCGGCACATCCGCCTTGAACACGTGCGCCTCGGGCGCTTCCTTTCAGTCGATGCACGCGCCCGTGAAGGCCGCCGTGTCAGAGGAGGTGCGCGGGACGAGGCTGCCGCTGCTGTTGCTGTCGGAGTCGAAAGGGAAGCCGTCGAAGGGGTCGAAGAA

>CLONE_148

ATCCTCCTCCGCCACGGGGACAGCGCGGTTGGGGAACGCTTCACCAGAGATCATGACAGGCCACTGAGCAAAGCTGGAAGAGCTGATGCAATAAGCGTTTCTGATAAATTCCATCAAATGGGATGGATACCTGAGCTTATCCTATGCAGTGATGCAACTCGTACAAAGGAAACTCTTCAGATCATGCAAGAGCATGTTCAAGGATTGTCTCAAGCACTTGTGCATTTCATCCCAAGTTTCTACTCGATTGCTGCAATGGATGGTCAAACTGCCGAGCACTTGCAAAAGGCAATTTGTGAATATTCAACTGATGAGATACTAACGGTGATGTGCATGGGACATAATAAAGGATGGGAAGAAGC

>CLONE_149

TTGCCCAATTTACTTGTAAATCTGAGCGCCATATTGGGACACAGTCAGGGGGCATGGATCAGGCTATATCTATCATGGCCAAACCTGGATTTGCTGAGTTGATAGATTTCAATCCAATTAAAGCAACCGATGTGCAACTACCTTCTGGTGGTACATTTGTGATTGCTCACTGTCTGGCAGAGTCCAAGAAAGCAGAGACAGCTGCTACAAACTACAACAACCGCGTTGTAGAGTGTCGCTTGGCAGCGATTGTTCTCGCCATCAAACTAGGGATGGATACAAAAAAGGCCGTCACGTCTGTTACAACCCTCTCTGATGTTGAGGGGCTATGCGTCTCCTTT

>CLONE_150

GAGATAATGCACAAAAGCCACGCAGAGATCCTGCTTCAGTTGGTGCAAACAGAGTTCCTCAAGGTGCTGCCGTTGCAAGGCCTGGTAAAGTGGTTGGTTCGGCACTTCGTTATGGTAACTGTTCAACATCTGGTACTGAGCAATATGAACAGCGAAGGGTCATCACAAGCCAAGGAATTGTTCCAAACGGCGTTCCTTCAGGCAGCTCATACCCTAGAAGAAATAACACCTGCAAGAGTGAAACAGGTGAAGCTGAAAGGATCGACGTGAGCCAAGCCGTGCCACCAAAGCCATATACCGGAAATAAACTACCTGCAACTGTGGATGGCCGCAATGGGCACTGG

>CLONE_151

TCTGATCCCGGAGTTGCTGTGAAGAAGCTATTGCATGAGGAGCCATATACTTTGGAAGAAATAGAGAAAATTACAGGTCAAAGCCTAGCAACTGTCTTCCAGAGCTCTCAAACTTCCTTGGATGTTTTGAGAGCTGCAAAGCATTTCAAGTTATTTCAGCGTGCCTCTCATGTGTACTCTGAAGCAAGGCGGGTGTATGCGTTTAGGTATACTGTATTATCCAAACTCAGCGATGAAGGTATGCTTAAGAAGCTCGGTGATCTAATGAACGATAGCCATCATAGCTGCAGTGTGCTATATGAATGCAGCTGTCCCGAGTTGGAAGAGCTTGTAAAAGTGTGCCGGGAC

>CLONE_152

GTTGATTGATTTTGGTCTCTCTGATTTCATCAGACCAGATGAAAGGCTCAATGATATTGTTGGAAGTGCATATTATGTTGCCCCAGAGGTTCTACACAGATCATACAGTATGGAAGCAGACATTTGGAGTATAGGTGTCATAACATACATTCTGCTCTGTGGTAGTCGGCCATTTTGGGCACGGACAGAATCTGGAATCTTCCGATCTGTATTGAGAGCTGATCCCAACCTTGATGATTCACCATGGCCTTCAGTGTCAGCTGAAGCTAAGGATTTTGTGAAAAGATTTCTTAACAAGGATTACCGCAAAAGAATGACAGCTGTCC

>CLONE_153

TTGATCTACTTGAACCTAGAGATGGAGTCGTGTCACTTGACAACTTTCGGACGGCTTTAACGCGGTATCTAACTGATGCAATGAAGGAATCAAGAATTCTTGAGTTTTTGCATGCGTTGGAACCACTTGCATACAGGAGTATGGATTTCGAAGAATTCTGTGCCGCGGCTATAAGCCCTTACCAGCTTGAGGCCCTGGACAGATGGGAGGAGATTGCTGGAACAGCTTTTCAGCACTTTGAACAGGAGGGCAACCGAGTCATATCAGTCGAGGAGTTGGCACAGGAACTAAATCTTGCTCCGACTCATTATTCCATCGTGCAAGATTGGATCAGAAAAACAGACGGCAAGC

>CLONE_154

TCAGCCTCTGCAAGAACATCAATTATCAACTGCTCAGCTCTGTTTATCTGATCAGGAGTGCCCGAAAGATCAACCGATCTTGTCTGTGAACCGGGTTGAACATCCATGTCCCTTGTTACTTGGATCTTCGCCCCTGACTGAGCTTGGAGATGCTTTATAGTTTCTCCAGCTTTTCCAATGATAACACCAACCCTTCCATTTGGGATATCAATCTTTTTTGTTGTACTGCCACTCTG

>CLONE_155

TATCAGAGGGGAGGAGCTGTACCGTCTTGCGAGGGACTACACTAGATATCTGGTTACTGGTAAGCGAACTGCCCGGCTGAAGCGCTCCATGCAACAATGGCGCAGTTTCTCGGAGAAATTCATGCAAAACGAGGGCTCTCAAGAAGAGCGGTACGAAAGGCCAGCTGCTACGGAACCCATGTGGTGGCAGCAGCCTCAAAAGTTTGTTCATCTTATGGAGGAGCTTTGCAGAGGGAACTGGCGTCCACATGCCCAGAAGTCTTAG

>CLONE_156

TACTTTCGGTCCAGGAGAGCAGCCTGAGCAAGCCCAATGGCAGCAACGGCTTCATTCGCGACCACAGCAGCGCCTACAATAGCCCGGAGTTTGCTTCGTCTTCTTCTTCATCGTCGTCCAAATTCCGGATGCCGACGATGATGTTCTCGTCGCAAAACGATCTGCTGCAGGAACAAACGCTGCACGCACGTCCTCCGGAGAAGAGGCAGCGTGTTCCTTCGGCGTACAACAGATTCATCAAGGAAGAGATACGAAGGATCAAAGCAAACAACCCCGACATTAGCCACAGGGAAGCCTTCAGCACTGCCGCAAAGAACTGGGCACATTACACAAACATCCATTTTGGTCTAAACCCCGAGCGCG

>CLONE_157

GTTGCCACAGAAGAGGAGCTGATCACTTCAGGGTATGATAACAAGGTCTTCAGAATTCCTGTTAATGGAGATCAGTGTGGAGACGCTGAGTCAGCTGATGTAGGGGGTCAGCCAAATGCTTTAAACATTGCACTTCAGCAACCTGAATTTGCACTGGTTACCACAGATTCTGCGATCGTATTGCTGAACAAGTCAAACGTCACTTCCACAACAAAAGTTAGTTACACTATCACTTCGTCTGCTGTTTCTCCTGATGGCACTGAAGCTATCGTTGGTGCTCAAGACGGGAAACTGCGGATCTATTCCATCAGTGGGGATACTGTTACAGAAGAAGCGGTACTTGAAAAACACCGGGGTCCTATTACTACCATACATTATTCGCCGGATGTTTCCA

>CLONE_158

CAGAAGCGGCTCCATGAACAACTCGAGGTTCAAAAGCAGTTGCAGCTGAGAATCGAAGCACAAGGGAAGTACTTGCAGATGATCATAGAGGAGCAGCAAAAGCTTGGTGGCTCACTTGAAGGTTCTGAGGAGAGGAAGCTTTCACATTCACCACCTACCTTAGATGACTACCCTGACAGCATACAGCCTTCTCCGAAGAAACCACGGTTGGATGATCTGTCAACAGATGCGGTCCGGGGTGTTACACAGCCAGGGTTTGAATCCCATCTTATTGGCCCATGGGATCAAGAACTCTGTCCGAAGACCAACATATGCGATCCTGCATTCCAAGTGGATGAGTTTAAGGCAAACCCTGGTTTGAGCAAGT

>CLONE_159

TTCCCTACGGCCACGTCCTCTCGGATTTCGCAAGCTTGATAAGCATATCTACCCACGTCTTGTTCTTGTTGCTGCTAGCCACCCAAGGCTTACTCCTGTATGCGCCTCAAGTGGAAAAGGGAACCCCGAGATTGACAATGATCCCTTTATGGATCATTTGAAGAAGGCCATGGCTGATGCAAAAAAGCCACGACCCATACAAGATGCGCTGAAAGAGAAATTCACCAAGCTGAGAGAACAAGCATCTGGTGGAGGTGGAGGGAATGGAAACAGGCGTGGAGGCAATGGTGGTTCCGGTGGCCCAGAAGATGAATCATTCAAGGAATCATTGGATGAAGTAGTCCAAGTTATCTTAGCAACTGTTGCTTTTATACTTGTGTACATCCATAT

>CLONE_160

AGTTGGTTCAACAAAAGAAGAGAGACAGAGCTTTGATTGCACTGAAGAAGAAGAAATCCCAGGAAGAGCTCCTGAAACAAGTTGATACATGGCAAATGAATGTCGAACAGCAGCTGGCAGATATTGAACTAGCAAGCAAGCAAAAAGCTGTATTGGAAAGCCTAAAGACTGGAAATGCTGCCCTTAAGTCTATACAGAGTGAGATTAACATCGACGATGTCCAAAAGTTAATGGATGACACAGCTGAGGCCAAGGCTTATCAGGATGAAATAAATGCCGCTCTGGGTGAACAACTATCTGCTGAGGATGAGGAGGCTGTCATGGCTGAATTCGACAACTTGG

>CLONE_161

TGTATTGTTAGGGTTGCAACACAGCTCTGTTGATCAGAACAGGGTGTACATGGAGTATGAAAGGTCAACCTGTGCACTGTTCAGGCCTGGATCTGCGCTTCCATTAGTCCAGTTCTGGTAGAATGTATCATACCCACCAGGGGCCATGTAGCTATTGGCACCTCCATTCCAATATTCAGGCATCTGATTTGGTCTATTGCTTACGGACGGAAAAGTGCAGCAGCATCTCATGGGGTTTTGATCCCCACCCATGGACTGGGGCAAGCCACCAGGGTATGGAGGTAACTGACAGTGATTTG

>CLONE_162

GCATTGTGACATGTGGTGAAGATTTTCTGGCAAACTACTATGAAGGACCACCATTCAAATTCAAACATTCCATAAGGGATCACACTAACTTTGTTAACTGTATCCGGTATTCACCTGATGGAACCAAGTTTATCACTGTGAGTTCAGATAAGAAGGGTTTAATATATGATGGCAAAACTGGAGATAAGATTGGAGAGCTATCCAGTGAAGGCAGTCACACAGGCAGCATATATGCTGTTAGCTGGAGTCCTGACAGTAAACAAGTTCTAACAGTTTCTGCTGATAAAACTGCAAAAGTATGGGATATCAGTGAGGATGGAAGTGGAACGTTGAACAGAACTTTGGTTTGTACTGGTATTGGTGGTGTCGATGACATGCTTGTGGGCTGCCTC

>CLONE_163

GCTGCTGCGCTCCACCCTGTGCCAGGTGTCAGTCTTCTCCTTCTGCTCCTTGTTCCGCTCGCCGCTGATCTGGATGATGTTGTCGTTCTCCACCTCCACCTTCACCCCCTCCTTCTTCAGCCCCGGCACATCCGCCTTGAACACGTGCGCCTCGGGCGCTTCCTTTCAGTCGATGCACGCGCCCGTGAAGGCCGCCGTGTCAGAGGAGGTGCGCGGGACGAGGCTGCCGCTGCTGTTGCTGTCGGAGTCGAAAGGGAAGCCGTCGAAGGGGTCGAAGAAGTCAAGGGAGAAGGGTTCAAACATGTTGCTGCGGCGGATCGGCGACATTGTCGGTCATATCGGTGAGGATTTGAGATGCTGGTGGAAGGAATCGACGAT

>CLONE_164

CACTGAGCAAAAAGGAAATCAAGTGGTCTGAGTACAAGAAGGTCCCGATATTGACGGTAGATGGTGAACATCTGGTTGATTCTACAGACATAATCAATATATTACAGCACAGGATCAGCCCTGATGATGAAGTTACTAACGAAGAGGAAACAAAATGGCGCAAGTGGGTTGATGAGCACCTTGTGCATGTATTGTCGCCAAATATATACCGGACGACTTCGGAGGCTCTAGAATCTTTTGACTACATTGCAAAGCATGGTAACTTCAGTTACACCGAGCGGTTTGCTGTGAAGTATGCTGGTGCTGCAGCAATGTACTTTGTGGCCAAGAAGCTTAAGAAGAAATACAACATTACTGATGAGCGCGCCTCGTTGT

>CLONE_165

GTATCGCCTCTTTCCACAAGAGATGAGACGGCTGGTGGTTCAACTGTGGGAACTGGCACTGAACCATCCAAGGCGGCTGAACAACCTGCTGCCAGCAGCAGTCAGGCAGCTAAGGAGAAAACTGGTGCTGATCCTGTCCTGGAAGAAGCTCCTTCTGCAACAGATCCAGATGCCTGGTCAGAGGCCCAGGTGCTGGCCGTTGTTCAAGCTTTGAAGGCGTTTCCCAAGGATGCAAACCAAAGATGGGAGCGAGTAGCTGCTGCTGTCCCTGGTAAAACAGTGGTGCAGTGCAAGAAAAAGGTTACCTCAATGAGGACGAACTTCCGGACCAAGAAGGCGGAGT

>CLONE_166

ATGATGCTGCCAACACGTGGACGGAAGCACTGAATGGAAGAAATTTTCTCGGTGGCTCCAAGCCTAACTTGGCAGATCTTGCAGCATTTGGCGTTCTGAGACCTATCAGATACCTGCAATCTGGAAAAGACATGGTTGAGCATACCCAAATCAGCGAGTGGTACCAGCGGATGGAAGATGCGGTCGGAGAACCATCAAGAATCCCGGAGGGCCAGTACCAGGAG

>CLONE_167

CCGGATTCGAGACCCCGGCCTCCACCTTCATCGCTGGAGCGAAGGTTAGAGTTGAGTGCAAGTCGAGATCTACTGGGGCCCAGACATGCAGCTTCGAGGGTCACACCGACCACACCGGTACCTACAACATTCCTGTGGCCGACGAGCATGAGCACGAGCTCTGTGAGTCTGTCCTCGTCAGCAGCCCGGACACGGCGTGTGCCAAGACAGTTGCTGGGCGGGAGAGGGCCCCTGTCTTCCTCAACAACAACAACGGCGTCGCGTCCAACGTCCGTTTGGCGAATGCTCTGGGGTTTGAGAAGGACACCCCTCTGGCTGCATGCGCGCAGATCCTCAAGATGTACGAGGAGGTCGATGAT

>CLONE_168

AGACAAAGGTGAATAAATATGCATTCTCAGGTGGCCAGGACTCTGTAGAACTTCACAGAAAACTTGGAGCTAACCTTGAGGTTGATGTCTCAATTAAATACCTGAACTTCTTCCTTGAAGACGATGATGAGCTCGAGCGCATAAAGAAGGCGTACAAGGAAGGAAGGATGCTGACGGGTGAAGTGAAGCAGCTTCTGGTTACGGTTCTTTCTGAGATGGTTGAAAGGCACAAAAGAGCTAGAGCTCGAGTTACCGAGGAGATGGTCGACGCCTTCATGGCTGTGAGGCCTCTTCCCAACATGTTTGG

>CLONE_169

GAGATCCCCTGAATTGGTCAATCAGCATTAGTTTGTTTGGTCGTTTGATCATGGACCCCTTTCACGGCATTGTGAAAGAGGAGGAATTTGACTTCGCCGGAGGTGCTGCTGATGGATACTCGCCGTCTTCGTGGGGCTCATCCCCGTCTTCCTGGGGCTCTTCCCCATCTTCGTGGGCCGGCGACGGCGCCTTGGCGGAGCTGCCGCGGCCGATGGACGGCCTCGGCGAGGCCTGCCCTACCCCGTTCCTGAACAAGACGTACGAGGTGGTGGACGACCACAGCACGGACACCATCGTGTCATGGGGCGTCGCCGGGAACACCTT

>CLONE_170

CGTGGTGTGGGACGCCCACGCCTTCTCCATGGTGCTCCTCCCCCGCTACTTCAAGCACAGCAACTTCTCCAGCTTCGTCCGCCAGCTCAACACCTATGGGTTCAGGAAGGTTGACCCGGACAGGTGGGAGTTCGCGGCGGAGGGGTTCCTGCGGGGCCAGAAGGAGCTGCTGAAGACGATCAGGCGGCGCCGGCCTCAGTCGTCGCCGTCTGGCACGCCGGCGCTGCAGCAGCAGCAGCAGGGCCAACAGCAGGAGGCGTGCCTGGAGGTGGGGCATTTCGGGCCCGAGGGCGAG

>CLONE_171

ATGCGGGCAGCCAGTTGGAATTATTGGAAACAATGGCATTTTATTTACCGAGTCGGCACTAAAGGGTACCCACTTCATTGAGTTGTGTGCTCAACGCAATATTCCTTTGATATTCCTTCAAAATATTTCTGGATTCATGGTTGGGTCGAAATCTGAAGCAAGTGGAATTGCGAAAGCTGGGGCAAAAATGGTTATGGCAGTTTCCTGTTCAAAGGTTCCTAAAATTACCATAATTGTCGGTGGAAGTTTTGGTGCTGGGAATTATGGAATGTGTGGACGTGCATACAGCCCGAATTTTTTGTTCATGTGGCCAACTGCTAGGATATC

>CLONE_172

CTGCAGCCCAAGGTGCAGCACCCGCCGGCCACCTTCTTCCGCTGCTACATGGACGCCGTTCGCGTCTCGCTGCTCATCATCGGGCCATACCAGCTCATCTCGTATCCTGCCGCCAAGATAATGGACATACGGACGGGACTTCCATTGCCGTCAATGGGGGAGATAATGGCGCAACTGACAGTATACTTCTTGGTGGAAGACTATCTGAACTACTGGCTCCATCGGCTGTTGCACACAAAATGGGGTTACGAAAAGATCCACCATGTTCACCATGAGTTCACGGCGCCTATGGCGTATGCCGCATGGTATGGACACTGGGCTGAGATGCTCATCCTTGCCGTACCCTCCTTGGCTGGCCCTGCTCTCGTCCCATGCCATGTTACCACGCTGTGGAT

>CLONE_173

ATTTGGCCGATGCCCTTGGGATTGTGGACAAGTTCTGGGTTGTAGGCTATTCTGGAGGTGGCATGCATGCTTGGAGTGCTCTGCGCTACATTCCTGACCGGGTGGCAGGTGCAGCAATGTTCGCCCCTATGGCAAATCCATATGACTCCAAGATGACCAAGGATGAGAAACGTAAAATATGGGACAGATGGTCAACTAAACGAAAACTAATGCACATTTTAGCTCGGAGGTTCCCATCACTATTACGCCTCTTCTATCACCGAAGCTTCCTTTCTGGAAAGCAAGGACAGCCTGAGAGTTGGTTATCATTGTCAACGGGAAAGAAGGATAAAACTTTACTGGAAGCTCCTACATTCAACACATTCTGGGAAAAGGATGTTGCAGAGTCTG

>CLONE_174

TGTTATGGGTGGCATTCAGGCAGCTGGTGTTCTCGCCCAAATAGAGAAGAACAACAGGAAAAGGCAAGGAGTGGAGTGGACCAAGGATGATGAAGAGGCCTTCAAAGCCAAAGTCGTCGAGGCATATGACAAGGAAGGAAGCCCGTATTACTCGACCGCTAGGCTTTGGGACGACGGGATCATAGATCCCGCAGATACCAGACGGGTTCTAAGCCTTTGCCTCTCTGCTTCGGCCAAGCCGGTTCCAGAAGACACGAAATATGGCGTGTTTCGA

>CLONE_175

TCAGATATTCAAATGCAGAAGAAAGAGGATCAGGGAGTATTTGAATTTATGAAGTCTCTGTTCAGTCAGGCTGAACGAGAGTGGGTGGGATTTCTGGGCCCAATCCACATCTGGCAGGGAATGGATGACCGGGTGGTGCCCCCATCGGCGACCGAGTTTGCCAGGAGGATGGTTCCAGGAGCCACTGTCCACAAGCTTCTCGACGAAGGCCACTTCTCATACTTCTGTTTCTGCGACGAGTGCCACCGGCAGATATTCTCCACCCTGTTTGGCACCCCGCAGGGCCCTCTCAATCCGGCACCGGAATCCAGTGAAGTGGCCCCGGAACCGGCGGAAGAAACAAGCCCTGCATACGAAGAAGTTGCAGAGCAGGAGC

>CLONE_176

AGTGAGCTGGAGTCTACCTCAAACATGCAAGTTGATGAGGACTCTAAATCAGATGATGAGATGGCTGATCTTGAAATGTTGGATCCCTCGGACTGCTTCATGTGTGATCTCAAGCATGACAACATAGAGGATTGCATGATCCATATGCACAAAAAGCATGGTTTCTTCATACCTGACAGTGAATACTTGAAAGATCCCAATGGCCTTCTTATATATGTCGGACTGAAGGTGAAGCGTGACTTTATGTGCCTCTACTGCAATGACAGATGCCAGCCTTTTCAAAGTCTTGAGGCTGTCAGGAAGCATATGGATGCAAAAGGTCACTGCAAATTGCGTTATGGAGATGGTGGGGAGGATGAAGATGCTGACCTTGAGGATTTCTATGATTACAGCAGCAGCTATGTGGATGTG

>CLONE_177

ATGGAGTTCAATGAGCCGGACCGCAGGAACAGAGGCGGCCAAGGCTGGAGAGCCATCATGTGGGATACGACGGTGATTTCAGATGGTTGGAGCCAGCGCTTCAGAGTTGATGTTGATAACATTTCGGTGGACAGAAGTTATTCTGGTCTACTGCCTCAGCTGTGGAATGGCGAGACCAGAGAACTAGAACTGAAGAGACTGATTTTGTATACCCCCACGCTGAGCGTGCACGACGACAATCTTCTTTACGTGATGGCTAAGATGAACAGTGAAGACGATAAGGCCTGGGTCATCACCGTCGACATGAAACGCGAGGCCGTGGAAGCAATCGCCCCGTATTCTACCAGAGGGCGCAAACTCACTTCATGGCATAGTCCATGCACCTTTCC

>CLONE_178

GAAGTACAAGGACGAGGTGCTGGACCCGCGGAAGAAGCACACCGAGAAGGAGAAGCTCGGCCGGAATGTGCCGCAGGTGTCGTCGGCGCTCTTCTTCAGCTACAACATGGCGCTCGGGCCGCCCTACCGGGTCATCGTCGACACCAACTTCATCAATTTCTCCATCCAGAACAAGTTGGATTTGGAGAAAGGAATGATGGACTGCCTTTATGCAAAATGTACCCCCTGCATCACTGATTGTGTTATGGCCGAGCTTGAAAAGTTAGGACAAAAATATCGTGTGGCTTTGAGAATTGCTAAGGATCCTAGGTTCCAGAGGCTGGCGTGCACACACAAAGGAACTTATGCTGATGACTGTATCGTTGAGAGAATTACTCAGCACAAATG

>CLONE_179

GGTAAAATATGCTTTCTCTACTGGTGAAGGCGAAGCTGCTCCCAAAGCTGTGGATTTATGCTCACGAGCTTTTGAAGACTTCGATGTGCTGCTACGCGAGTTATTGCATGATCCAGCGTCATCTACCGAAGGCATGAGTTGCAAAATCAGTGTTGCCCTTCGAGCTTTAGACAGTATCTTGGACATTGTGCCACCAACCTTGAGGGCGCTTGCGGATGCCTGTCATCAAAAAGGAGGGAAAACAATATTTGATCTGGGTGAGGAGCCTGGTGATCCAAAGGACTTGAAACTACAGTGCTCTCCTAAAGCTAACAACCTTACATGGTGCAAGCAGCCAGTCGTCTATTCAAAGAACAGATCTGACGAGTTGCAGCAGTGGGGATCCAACCT

>CLONE_180

CTCAAGGCCAAGGGCCTTGACGCCATGCTTGAGCTGATGGAGGATGAGACGATGACGTCGTTCTCCCTCTCTTCCTCGTCGTCAGACGACGACACCGGCCGGCCGCACCTGAGGCGCCCGTCGTCGGGGAGCTTTGGGCGCCGGTCGACCGAGGAGCCCGTGGTGTGCAGCCCCGCGAGCTCGCTGGTGGCCGTCATGGTGCAGGCCCTCGCCCACCGCGTGAGCTACCTGTGGGTTCTCGACGAGGAGGACGACTGCCGTCTCGCCGGGATCGTCACCTTCGCCGACATCCTCAGGGTGTTTCGTGAACAGCTGCA

>CLONE_181

GATTAACCGTTTCCAGATAGGAGGGAGGACGGTATTTGATCTTGTTGAGCAGGCAAGAGATACAGAGGACTGGAAAGTGGAGAGCTCTTGTGATGCTAACAACCTTTCTGGCGGCAAGGACAAGGGCGGATCTTACGAGGTGCAGCAGGGGAGATCCAAGCCCATTAACCGCCGGCAAAGGAAGCGGAACCAGGCACGGGGCAGGGAAGCTGGCCGTGATGAACGATTGGTGGTTCCATATTGCCGCCAACTGGAGCAGTATGTGTTGATTTTCTTGTGCCTGC

>CLONE_182

AGGTTGAATTCAGAAGGTATTTTGAGCAATTCGGTATGATAACAGATGTGGTTGTCATGTACGACCACAACACACAGAGGCCCAGGGGCTTTGGCTTCATCACCTATGATTCAGAAGATGCAGTGGATAAGGCGCTGCACAAGAACTTCCACGAGCTTAATGGCAAGATGGTTGAAGTCAAGAGAGCTGTCCCAAAGGAGCAGTCGCCTGGACCTGTCGCACGCTCACCTGCTGGAGGGCAGAACCTTGCTATCAGCAGGGTTCACAACTTCTTGAATGGCTTCAACCAGGGATATAGCCCAAACCCGATAGGAGGTTACGGCATGAGAGTGGATGGAAGGTTTGGGCTGCTTTCAGGTGCACGAAATGGGTTTTCTTCATTTGGCCCCAGTTATGGAATGGGC

>CLONE_183

GCTGAAGCAGCGCAGAGCTTGCATCAGGAAAACATGATGCTCAAGGAGCAGATGACGGCCGTCCTGTCGCAGAACGCGGTCTTGAAGCGCGCGGTGGCGATCCAGCACGAGCGGCAGAAGGAGTTTGATGAGCGGAGCCACGAGGTGCAGGGCCTGAAGCAGCTCGTCCTGCAGTACCAGGAGCAGCTGAGGACTCTCGAGATCAACAACTACGCGCTGCAGATGCATCTGAAGCAGGCCCAGCAGAGCAGCTCCATGCCCGGGCGCTACAACCCGGACGTCTTCTAA

>CLONE_184

GCAAATGGGTTCATACTACAATGGTGGTTCAAACAGACTAGGCAGCCCTATTGGGTATGTTGGTCTGAATGACGATTCAGGATCAATATTGAGTTCAATGGGAAGGAATGTTTGGGGTAATGGAAATGTCAACTACCAGAACAGCCCTACAAACATGAGTTCTTTTGTACCATCTGGAAGTGGGAGTCAAGTTGGTATTACTGGCGACGGTATAAATTGGGGAGGTCCTACTTCTGCCCATGGGATGGGAAGCATTTCAAGCCTTGGGTCTAACATTGGCCGTGGGGCTGGAGATAACTTTGGCTTGCCGTCTGGTGGCTATGGAAGGAGCAACCCAACTGGCACCATTGGTGAACCTTTTTCTGCGTCAGCCAATGCATATGAAATGAACAACATAGATACATATGGCAACAACTCTATTTATGGTGACTCAACCTGGAG

>CLONR_185

TCTTCTCAGGGAATCATATTGGTTTGGAGTGAATCAAAGAAAAGATTATCTAGCTTCACAGTCAATGGAGTCCCTATTGCTACCTCAGTTCTATCACCTTTTTCTGGGGGAGTTAGTTGCATTGAGATTTCTATGGATGGTCATTTTGCCCTGATTGGAACTTGTTCGTCCAGCAATTACAAGTGTGAGGACAATACTGAGGTAGCAGATCATGAGCCCAACAAATCAAGTCGCAAGGTTGATATATCTGAGC

>CLONE_186

GTTCAGATAAGGGCAATAAACATGGCAAACGGTAAAAGCTTGCCTGAAGTGCCGACCTTGATGTTTGAATTCATAGGGACAGAAGCATATGCACTTGAACAAACACTGTTGGTTCAAAAGATTGCTGCAGAGCACCATGGTTCTGATTTTATTTTTGTGGAGGAGCCAAATGCTAAAGAGGAACTGTGGAAGATCAGGAAGGAGGCACTTTGGGCTGGTTTTGCCATGAAACCTGATCATGAAGCTATGATAACGGACGTTTGTGTTCCATTGTCTAGACTTGCAGAATGCATATCTGTATCTAAGCAACTACTTGATGCGTCACCATTGACTTGTTTGGTTATCGCCCATGCCGGTGACGGAAATTTCCACACAATTATCCTATTTGATCCAAGCCAGGAGGAAGAGCGAAGGGAA

>CLONE_187

GCTCCAGTTAGCAAAGGTGCTTATTCGCAGGTTGGATACTCATATAAAGGGGATGGAAATGAACATTCTGATGTGCCCAGTGATGATGAAGATGAAGAGGAAGGAGAAGAGGATGGCAAGGACTTCAGTAGTGATGATAGCAGTGATGAGCAAATGGAAAACTTAGCAAAGGAATTTGGCATAAAAAGATATAACTGGCTTGTTTACATGGACAAAAAGGTCAAAGAAGAGGAGAAGCGGCAGAAAGAAATTATTAAAGGCGACCCATCCATTAAAAAAATGAGTCGGAGAGATAGAAGGAAGGCTTCTCAATCTGAGAG

>CLONE_188

AAACTGAAATCAGGCAATCTGTTAACGTGCCTTCGATCTGCTTTGTTGATCTGCATAAACTTAAGGTGTTTCATACACTGGAGCTTGGAAAGGGCCAGGACATCACAGCAATTGCATTAAACAAAGAGAACACTAATCTTCTAGTTTCTACTGCCGATAAGGGCCTAATGGTTTTCACTGATCCTGCTTTGAGCCTGAAGGTAGTGGATCAGATGCTCCGCCTTGGCTGGGAAGGTGACGGACTTCTTCAGTC

>CLONE_189

TCCAGACGTCATACCCGTGGTACACATGCTGATAGTAATTACCGAAGCAAGGCTAAGCCTCCAAGAGTTGAATACATCACTGAATTTGGAGGTTCAGAAGACGCCAGTGATCTAAAAGTTACAGGGATCTCTCCGCCTTCGTCTCCAATACGAGCTGACATACCTAACCGGTCATCAGGTGTCCATATTCTGGAGGCACTTCACAGTGATCCTGCATCTTCTTTATCCATGGAACAGGAAAAAAGTGCTAAAATTTTGAAACCACCTGCCAGCACATCATCAGCACTAGCGAAACTAAAGGGTGCTTCTGGAGGACTTGGTAAAAC

>CLONE_190

TCCGCTACTTCCTCAACTCCATTTCCCTCTTCTACCATGTCGCCGCCCGCTCCGTCTCCTCCCTTGGCCTCGTGCGCCCCGACTTCCTGTCGGTGCGGCCCGACGAGGCAGCCCTGTCCGCCGTCCCCCTCATCCGCCGGGCCATCGCCGCAGAGACCGCGGTCGCCGTGGTCAGCACCGACGGCCACCTCGTCGGCGAGATCTCCACCGCGCACCTCGCTGCCTGCGACGAGACGGCAGCCGCGGCCATCGCCACGCTCTCGGCGGCCGACCTCATGGCATACATCGACTACTTCGGCTCGCCGCCAGAGCACATCCTGCGCGCCATCAAGACCGGG

>CLONE_191

TGGGATCCACCGCCGTCGTGGCAGTCGTCAGCCCGACCCAGCTCGTCGTGGGCAACGCCGGCGACTCCCGTGCCGTCCTTTCCCGCGCCGGCGTCCCCGTTGCACTCTCCGTCGACCACAAGCCTGACCGGCCGGACGAGCTGGAGCGCATCCAGGCGGCGGGCGGGCGCGTCATCTACTGGGACGGCGCCCGGGTGCTCGGCGTCCTCGCCATGTCCCGAGCCATAGGGGATGGCTACCTGAAGCCGTTCGTGACGGCGGAGCCGGAGGTGACCGTGACGGAGCGCAGCGACGCCGACGAGTGCCTGATCCTGGCCAGCGACGGGCTGTGGGACGTGGTGACCAACGAGATGGCGTGCGACGTG

>CLONE_192

GGCCTGGAAAAGACCCTGTGCTTGGGGTTGACAGTGTTGGTACAGTATCCGCAGCTCTTGTGACTTATGCTTCTATACAATTGTTGAAGCCAGACCTTATCATCAACGCTGGTACAGCTGGTGGTTTTAAGGCCAGAGGAGCAGGTATTGGGGATGTCTTCTTAGCTTCAGATGTTGCTTTCCATGACAGGAGAATACCCATTCCTGTTTTTGACAGTTATGGAATTGGAGCACGAAAAACATTTGAAACCCCAAATATAGTGAAGGAACTCAATTTGAAGGTTGGGAAACTGTCAACTGGTGATTCTCTGGATATGTCCCCCCATGATGAGACAGCAATACTGAGCAATGAAGCTACA

>CLONE_193

ATTTGGATGATGCAATAAAGAGTCTGAAGGAGCTGCACCTGATGGAGTCAAATCAGGCTAACCTGTCGGCCACTGGTTCCGCATTTGAAAACGGGCCGACTGCAGTTCAGCCGTCTGTTGAAGGCATTGTTACCAGCGGCGGTGTGGACACAGCTACTGAACACCAACCTGCTGCAGATGGCCAGCAGCCAAGTAATAGTGGCCCTGAATGGGTTGATCTTTTTGTGAGGGAGATGTCAAATGCTTCTGACATGGATGACGCGCGGGCCCGTGCGTCAAGAGCTCTTGAAGCCTTGACGAAGTCCATCCTGGAGGGTGCAGGA

>CLONE_194

ACCAAAAGCTTGCTTGATAGCAACTTGGAGGATGGAAGATACATATCTGCCCAGGGTAAGAAGGTGGTGGTCATTGGTGGTGGAGACACAGGCACAGATTGCATTGGTACGTCTGTTAGGCATGGTTGCAGCAGCATTGTAAATCTGGAGCTTCTCACCAAGCCACCAAGCAAGAGAGCTTCTGACAACCCCTGGCCCCAGTGGCCTAGAGTCTTCCGAGTGGACTATGGGCACCAGGAAGCATCTACCAAGTTTGGAAATGATCCAAGAACTTACGAAGTCTTAACCAAGCGTTTCATTGGTGATGAAGATGGAAAATTGAAGGCCCTTGAGGTGGTGCGCGTGAAGTGGGAGAAAGT

>CLONE_195

CTGGTTTGCTGCGCGTTTGGTTGAGAGCCTCAACATACATAGCGGATTTAAGTTGCCATTCAATGCTGAGAAGTATATACCGTTCTATGGAGGGGCGGAGCACCATGACTACCATCACTACATAGGAGGACAGAGCAAGAGCAACTTCGCTCCTGTTTTCACCTACTGTGATTATATTTATGGAACGGACAAGGGCTACAAATATCACAAGGCAACTCTGGCAAAGCTGAAGGAGTTGGCAGGCAGCGACGTTCAGAAAGGAGCCGACAATGGATTCAACGGTGGAAAGCAGGAC

>CLONE_196

TTGAGGTATCAAAGGATATCAAGTATGCTGACAATCAACCAATAGTTCCTTGGGGACCTAGACCCTCCAAATCAGCAGTACAGGATGTGCGCATAAATATGGCAATATCAGCAGCAATTGTTGTTTGCATTGCTATCATCGGTAATGCAGACTGGAAGCCTTTGCAGTTCTTGTGTTTTGCTTTCTTCTACAGAATACTTCAGAAGCTGAGGGTTACTGAGCCACCAATAACTCCAATATATAATGAGTATGGTGAGGTTGAGGGAAGAGGGGTACGAATGGCAAAACGTGTATTCCACGCTTTGGGTTTGATATTTGGATGTGTATTCGCTGC

>CLONE_197

ATTAAGCAATACCTTCGTGCCACTCCTCTTAAACGTTTGGCGCTAAAGGCATTATCCAAGGCTTTAAAGGATGACGAACTCTTATATCTTAGGCTACAGTTTGATCTACTTGAACCTAGAGATGGAGTCGTGTCACTTGACAACTTTCGGACGGCTTTAACGCGGTATCTAACTGATGCAATGAAGGAATCAAGAATTCTTGAGTTTTTGCATGCGTTGGAACCACTTGCATACAGGAGTATGGATTTCGAAGAATTCTGTGCCGCGGCTATAAGCCCTTACCAGCTTGAGGCCCTGGACAGATGGGAGGAGATTGCTGGAACAGCTTTTCAGCACTTTGAACAGGAGGGCAACCGAGTCATATCAGTCGAGGAGTTGGCACAGGAACTAAATCTTGCTCCGACTCATTATTCCATCGTGCAAGATTGGATCAGAAAAACAGACGGCAAGC

>CLONE_198

GAAGCACTTCAGACGTCAGCGACGGAGCGGCGCGCCCCGAATTGGACTGGAACGCCACTAGGCTAGGTCTGCTTTCGGCCGCCCACGCAACGTGCAGGTGTGCTCAGGGCGATGGGCCCAGACCCCTGCGCGCTTAGGTTTAGACCGGCGTGCTGGCCTCTCTGTTGTGCCTAGGTGGGGCTGCAACGTGTTGATCTTCCGCGGCCGGACCTCGGCCGCGACCACGCTAATCACT

>CLONE_199

AACCATAAACGATGCCGACCAGGGATCGGCGAATGTTGCTTATAGGACTCCGCCGGCACCTTATGAGAAATCAAAGTCTTTGGGTTCCGGGGGGAGTATGGTCGCAAGGCTGAAACTTAAAGGAATTGACGGAAGGGCACCACCAGGCGTGGAGCCTGCGGCTTAATTTGACTCAACACGGGGAAACTTACCAGGTCCAGACATAGCAAGGATTGACAGACTGAGAGCTCTTTCTTGATTCTATGGGTGGTGGTGCATGGCCGTTCTTAGTTGGTGGAGCGATTTGTCTGGTTAATTCCGTTAACGAACGAGACCTCAGCCTGCTAACTAACCTGCCCGGGCGGCCGCTCGA

>CLONE_200

ATTCATTCCCATTTCTTGCATGGAACCTATGCATGCTCCCATTTACAAAGATTTGATTTTTCAGTATATTTATATGCACTGGAGCATGCGCATGTAACTCAAATTTGAATTATGCACATAAATGCATTGAAAACTCGAATAATGCATAAAAATATCCAAACGAGCCCCCATAAATCACAAAAATTCAAACAACACTCCTGTTGTTTGATGTTGACACGAAAAAAAAATTTGAAAGCAATAAGAGGTAATGGATATTGTTTCGTTCCCAAAGGTGGGACGTTCCCTACCGAACCTGCCCGGGCGGCCGCTCG

>CLONE_201

TTGCACGTGCATTGGATTAAGCTGGAATCTACAAGCATTCAACTCAAGAGAGAAGACAAGTAATATGGGCTCTAAGTTAAATAATCATTCATGCATATATGAGCCACTAAGCATTTTCAATATGGTCTTCTCGACCCCCAAAGAAAGGAAAAGAAATAAAAACTATTTACACGGGAAAGCTCCCAACAAGCAAAAGAAGAACGGGAAATATTTTTGAGTTTTTCCTTTTTAATTAGTACCTGCCCGGGCGGCCGCTCGA

>CLONE_202

CAACCATAAACGATGCCGACCAGGGATCGGCGGATGTTGCTTATAGGACTCCGCCGGCACCTTATGAGAAATCAAAGTCTTTGGGTTCCGGGGGGAGTATGGTCGCAAGGCTGAAACTTAAAGGAATTGACGGAAGGGCACCACCAGGCGTGGAGCCTGCGGCTTAATTTGACTCAACACGGGGAAACTTACCAGGTCCAGACATAGCAAGGATTGACAGACTGAGAGCTCTTTCTTGATTCTATGGGTGGTGGTGCATGGCCGTTCTTAGTTGGTGGAGCGATTTGTCTGGTTAATTCCGTTAACGAACGAGACCTCAGCCTGCTAACTAACCTGCCCGGGCGGCCGCTCGA

>CLONE_203

GTTGAAAATAGCCCGTGCTACCGTCTCCCATCGGATAGATCCAGTGACTATACGCTCCCTGGCATCCGTCGGAGTGAGTAGCGACCACATACGGATCAACGCAGTGGCTCGGAAGATAACCTGCAAAAAATGAATATCTGTTGTTCTGTTAAAGACCAAATCATTTCTGCAGTTCCAGACTGCCCATAATAAAGCACATACTCCTACACGAATGTGTCTCGCTGTTTCGGAATCTATCCCGTCAAGCCACGTCCCAAATAACGTGCTGATAGTATTCGAAGGAGTAATATTAAAGGCTATGTGAACCGTCCACCATTTTTTTTGCCAACGAGCAATCAAGAAAGAGGTGTTTGATGGATTCATCCCGATCACAAAAACTACATCTTGTAGATCCTGTCCAGTTGCGTTTTGCCAAGTTGTCCTTAATTAAAATGACTTGTTTATGTACCTCGGCCGCGACCACGCT

>CLONE_204

GTTTAATGGTAAATCAGCAAAGAAGTCACCGACCTGGTTGATTTTTCTTGTTTTTATCCGTTATGGCTTTTGATGGTAGAACCCAATCCGTTTTTTGGATAGAATGGAAAAAAGGTAGTAACCACTCTCTCTATATAAATCTACATCATACAATAATTAACATGCTTGTATTTCACCTTACAAAAGAAATAAAGAATTGTCTTCAACATAACTGCTACCCGCAAAAATAAGAGACATGACACCCGCAAAATAAACGACATGTTGAAGAGGAAGGTTGTAGTTACATATGATGTCTTGTAGTACCTCGGCCGCGACCACGCT

>CLONE_205

ACGACTACATCAACCGCGTTGTGCTAACACTTCTGCTTTCAGTCTACGAGGGTATGTGGACAACACTCTCCCCTCTCATTGCTATGCATCACAATGATCTTGCATGTGCGTAGGATTTTTTTTGAAATTACTACGTTCCCCAACACATATTACATCCACTAGGCATCAAATTTCACCACCAGTGCAAATATAGGGAACAAGGCAACATATTACATACACTGGCCATCTAAATTGGCCACCAGTGCAAATAAATGCGGTAACACAACAAGTCCATAACTGAAACAACTTAAGAAGAGCTCAAGAAATGTTATCCTGGGTATCCACCATGCTGGCAATAAGCTTAGCAAGCTTATTAGCTTTGTCTTGTTTGGCGTTAAAATCCTCCAGCGCTTGTTGTTGCACCAGAAAGTATGCATCTAAATGCTCTAGGGACTTTTGCAGTCCTTCGTCTTCTTGTCGCAGCACAGCTAATCGATGTCTTTCAGCTTGTAGCTGAGACTCAAGAAACCGAACTGATTCAGACAGTGAGTTTGAATAGCTTGTGCCAGTGGTAGTGGCCAATAACTCGAACACTAAACCAGGACAGGACTTTGGGGTTGTGTCACTGTCTTCAAGATAGTTTTCCTTACCTGTTTTATCAGCTTTCTTGGAGACCAACAAGGATGTCTCACTATCCTGAACCTTA
